# AIDE_long_—acute illness and depression in elderly: sustained improvement after group psychotherapy in geriatric patients, a follow-up of longterm effects in a randomized controlled trial

**DOI:** 10.1186/s12877-026-06983-0

**Published:** 2026-02-07

**Authors:** Jana Hummel, Cecilia Weisbrod, Leila Boesch, Katharina Himpler, Ilona Dutzi, Benito Baldauf, Peter Oster, Daniel Kopf

**Affiliations:** 1 University of Applied Sciences Bremerhaven, An Der Karlstadt 8, Bremerhaven, 27568 Germany; 2 Geriatric and Gerontopsychotherapeutic Practice, Rheingoldstr. 41a, Mannheim, 68199 Germany; 3 Geriatric Center Bethanien, Heidelberg, Germany; 4 Department of Geriatrics, RKH Kliniken Ludwigsburg-Bietigheim, Bietigheim-Bissingen, Germany

**Keywords:** Geriatric depression, Cognitive behavioural therapy, functionaless

## Abstract

**Objectives:**

Comorbid depression is highly prevalent in very old adults hospitalized for acute medical illness. We have previously shown that immediate intervention with cognitive behavioral therapy (CBT) effectively reduces symptoms of depression and anxiety. The present study examines the long-term course and the effect of delayed intervention.

**Design:**

Randomized, controlled cross-over trial of group CBT.

**Setting and participants:**

We recruited in-patients of a geriatric university department ≥ 65 years with depression (Hospital Anxiety and Depression Scale HADS ≥ 7). Intervention took place after hospital discharge in a day care setting.

**Methods:**

Patients were randomized to an immediate active intervention group (II) or a waiting list control group with delayed intervention (DI). II patients were invited immediately after discharge to 10 to 15 weekly behavioral group therapy sessions. After 4 months (T1), DI patients switched to active intervention, while II were followed under control conditions. Final evaluation took place after 12 months (T2). Primary endpoint was improvement in HADS.

**Results:**

56 patients (82.0 ± 6.2 years, HADS 18.8 ± 7.0) were randomized to II, 99 patients (81.9 ± 5.9 years, HADS 18.1 ± 8.3) to DI. II patients improved significantly at T1 (HADS 10.4 ± 5.2). Improvement was sustained under control conditions at T2 (11.9 ± 7.8). DI patients did not improve on waiting list (T1 22.9 ± 8.3), but after initiation of active treatment (T2 16.0 ± 8.5) (ANOVA: *F =* 3.75, *p* = 0.026). Concomitantly, functional parameters such as Barthel Index and Timed-Up-and-Go differed significantly between groups with better courses in DI patients. Among II patients, 4 (7.1%) deceased and 2 (3.6%) were newly admitted to a nursing home, among DI, 15 (15.2%) and 10 (10.1%) respectively.

**Conclusions and implications:**

Cognitive behavioral group therapy yields sustained improvement of depressive symptoms in very old geriatric patients, if administered in a multimodal approach immediately following hospitalization for acute medical illness. Concomitant with improvement of depressive symptoms, patients benefit in terms of functional status and medical outcome.

**Trial registration:**

www.germanctr.de; DRKS 00004728; February 12, 2013.

**Supplementary Information:**

The online version contains supplementary material available at 10.1186/s12877-026-06983-0.

## Introduction

Depression is a frequent comorbid disorder in geriatric in-patients suffering from acute somatic diseases or deterioration of chronic diseases, such as heart failure, COPD, stroke, falls, fractures, pneumonia, or other typical geriatric disorders [1–4]. A recent survey found a prevalence of 27% for depressive disorders in hospitalized geriatric patients [5]. Comorbid depression is associated with delayed recovery, frailty, functional decline, increased nursing home admission and mortality, which is true both for major depression and subthreshold depression [6–9].

Pharmacological treatment for late life depression is effective but limited by increased vulnerability and drug side-effects such as hyponatremia in the context of the syndrome of inadequate antiduretic hormone (SIADH) and anticholinergic properties of antidepressant drugs [10, 11]. In the last two decades, several psychotherapeutic approaches, mostly based on cognitive behavioral psychotherapy, have been adapted and proven effective for application in late life [12, 13]. However, patients at the more vulnerable age groups beyond 80 were rarely included. Most studies were designed for physically fit ambulatory patients who can attend psychotherapy sessions on their own. Thus, it has been noted that the influence of physical diseases, frailty and cognitive disorders on the efficacy and feasibility of psychotherapy has not been assessed in high quality studies [11]. We have recently reported first results of a randomized controlled cross-over trial of cognitive behavioral group therapy in geriatric patients who have been recruited during a hospital stay for major medical illness or trauma [14]. We found that even in this setting of very frail and highly vulnerable older patients group therapy is feasible and highly effective, both in symptomatic improvement and in functional outcome.

In the present manuscript we present data of an extension of this study including up to 12 months after randomization. The study was continued in a cross-over design, which provided active treatment to subjects that had previously been assigned to a waiting list. This study extension was designed to answer the following questions: (1) Will favourable treatment effects, measured by anxiety and depression symptoms and secondary physical functional, cognition and psychosocial parameters, be sustained after the end of active intervention? (2) Will subjects who have previously received treatment as usual now benefit from delayed active intervention?

## Methods

**Fig. 1:**
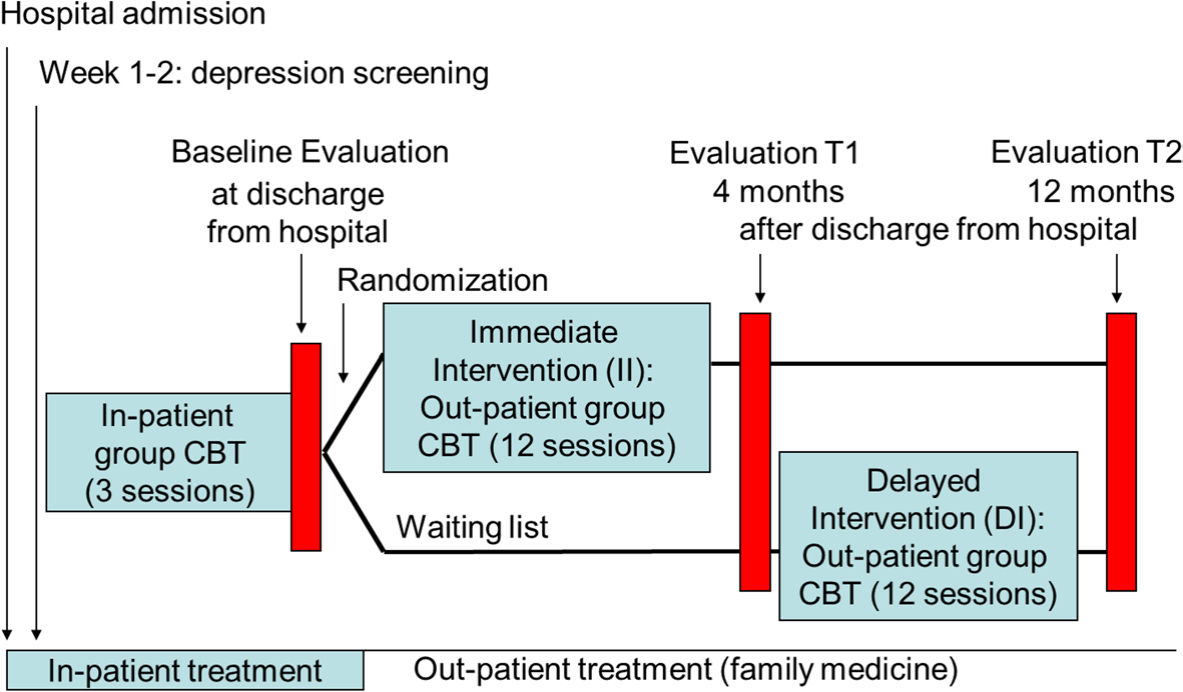
Study protocol: CBT cognitive behavioral therapy

The study design has previously been described in detail [14]. In brief, we recruited participants from patients treated in a geriatric hospital for medical or neurological conditions or fall-associated trauma. Subjects were eligible if they fulfilled the criteria for a minor or major depressive episode as detailed by Diagnostic and Statistical Manual of Mental Disorders, 4th Edition [15]. We performed a randomized controlled cross-over trial comparing patients receiving cognitive behavioral group therapy (CBT) versus waiting list, with cross-over from waiting list to active treatment after 4 months, while initial active therapy participants switched to no active therapy (Fig. 1).

### Participants and recruitment setting

Recruitment took place in a 170-bed geriatric university hospital in Heidelberg, Germany, in the years 2011 and 2012. Patients admitted for an acute physical illness were eligible for the study if they were 65 years of age or older and had a score > 7 in the Hospital Anxiety and Depression Scale [16]. Exclusion criteria were dementia (Mini-Mental State Examination) [17], delirium (Confusion Assessment Method, CAM) [18], severe medical illness with a life expectancy less than one year, and communicative or physical impairment that would impede participation in a group setting.

### Study design

The study was designed as a single center randomized, controlled cross-over trial. Randomization was preceded by evaluation of the in- and exclusion parameters, an in-hospital run-in phase, during which eligible patients were invited to attend two or three weekly CBT sessions, and informed consent (Fig. 1).

Randomization was to either an immediate intervention group (II) with active psychotherapy or a delayed intervention group (DI) on waiting list and subsequent delayed intervention. To account for potential attrition in the waiting list delayed intervention group (DI), randomization was performed in an unequal 1:2 fashion by lot with 60 lots II, 120 lots DI in a closed container. Lots were taken out blindly by the study head. After randomization, patients received a comprehensive assessment of depression, physical health, cognition, mobility, and functional status (= baseline, T0). This assessment took place prior to discharge from hospital. Subjects in II were invited to continue group psychotherapy as outpatients for a minimum of 10 and a maximum of 15 sessions. After four months of active treatment, primary outcome assessment was performed (T1). Thereafter, DI patients were invited to an active treatment course, while active treatment in the II patients was finished. 12 months after randomization, the final assessment was performed (T2).

Patients who fulfilled the inclusion criteria but did not consent to randomization or psychotherapy were asked to be included in an observational group (OG), who did not receive any active psychotherapy. This group was followed to assess the natural course of the disease.

### Intervention

The active intervention consisted of a manualized CBT program of 15 sessions [19]. The manual was adapted based on a well-established standardized manual for elderly, home-dwelling patients [20]. The first and the last sessions were individual, the others in open groups. The 90-min group sessions were conducted by experienced psychotherapists with qualifications in gerontology.

The CBT was embedded in a multimodal approach and provided in the setting of the hospital’s day clinic. This setting included once a week pick-up of patients at their homes by common transportation in a minivan, the group psychotherapy session, common lunch, an additional group treatment session of physical therapy or ergotherapy, and transportation back home.

The DI patients received treatment as usual from their general practitioner for 4 months without contact to and influence from the research team.

Agents and doses of psychotropic and antidepressant drugs given by external doctors were evaluated at baseline and after 4 and 12 months and included in the statistical analysis.

Patients who participated in less than 10 CBT sessions or who did not complete assessments at every time up to T2, were treated as not fulfilling the protocol and included in the intention-to-treat analysis. The study design was approved by the ethics committee of the medical department of Ruprecht-Karls-University Heidelberg, Germany (S-025/2010).

### Instruments and outcome measures

As assessment instrument for the primary endpoint we applied the Hospital Anxiety and Depression Scale (HADS) [16]. We chose this scale, because it had been designed for the use in the hospital setting. Its design also accounts for the observation that in older patients there is considerable overlap between depressive and anxiety symptoms. Additional assessments were performed using the Hamilton depression scale (HAMD) [21], Mini-Mental State Examination (MMSE) [17], Barthel Index [22], Instrumental Activities of Daily Living [23], Social Situation Scale (SoS) [24], Pearlin sense-of-mastery scale [25], Groningen Frailty Indicator [26], Cumulative Illness Rating Scale (CIRS) [27], Karnofsky Performance Status Index [28], Tinetti Test [29], Timed-Up-and-Go Test, frequency-of-going-out per week, resource utilization, and (psychotropic) medication after 4 and 12 months. This assessment at T0 took between 60 and 90 min. The assessment was performed as a standardized evaluation conducted by an independent clinician (medical doctor with geriatric and psychotherapeutic qualifications).

This assessment was repeated after 4 months (T1, primary outcome), and after 12 months (T2). The results at T1 have been reported previously [14]. This publication focusses on results obtained after 12 months (T2).

The primary endpoint of the study was improvement of depressive symptoms as assessed by the HADS total score after 4 months [14]. In addition, a clinician-rating scale (HAMD) was applied.

Secondary endpoint measures were depressive symptoms after 12 months [16, 21] functional [22, 23], cognitive [17], and psychosocial parameters [24, 25], physical health status [26–28], physical performance [29], resource utilization, and (psychotropic) medication after 4 and 12 months.

Patients’ physicians and in-home family caregivers were interviewed separately about the patient health status and the time required for the care. In addition, we assessed caregivers’ burden of care and depressive symptoms using the Patient’s Health Questionnaire [30, 31].

Follow-up examinations T1 and T2 in the OG consisted of telephone interviews of patients and caregivers rather than in-person-interviews, comprising HADS, HAMD and Barthel-Index [16, 21, 22].

### Statistical analysis

Before recruitment, a sample size calculation suggested a significant difference of the HADS total scores with a sample size of 64 patients (32 per group) at T1 (estimated baseline median HADS total score 18þ−7, relevant difference after 4 months, 5 points achieved by 50% in the II and 0% in the DI, powered at 80%).

Analysis was performed by both an intent-to-treat and a per-protocol analysis using the statistical program SPSS20 (SPSS Inc, Chicago, IL). For per-protocol analysis, only patients, who completed the entire study, were evaluated. For intention-to-treat analysis, missing values of dropouts were treated in the “last observation carried forward” manner. Baseline parameters of study groups were compared by one-way Analysis of Variance (ANOVA). To analyze the 12-month course of continuous variables, a two-way Analysis of Variance (ANOVA) was applied with group assignment and time course as independent variables and the studied end-point as dependent variables. ANOVA-results are reported as tests for “time”, denoting significant changes in the entire study population over time, and as “interaction time*group”, denoting differences in trajectories between groups. To test for group differences at different time points, post-hoc t-tests, were performed. Dichotomous variables such as survival or nursing home admission were analyzed using the Chi Square test.

## Results

In total, 155 patients were randomized. Patients entered the study at a mean age of 81.8 ± 6.4 years (range 64–96 years) with no significant differences between groups. 56 patients were assigned to immediate intervention (II) and 99 patients to the waiting list delayed intervention group (DI). Forty-four patients in II and 30 patients in DI completed the whole course of the study and were evaluated in a per-protocol analysis. Thus, drop-out rate was 21% in II. In contrast, in DI drop-out rate amounted to 70%. 91 patients agreed to be followed in the observation group without intervention.

There were no meaningful group differences in any parameter at baseline (Table 1) except for the proportion of patients with recurrent episodes of depression in history compared to first episode patients: Among II patients, almost two thirds suffered from recurrent episodes, whereas in DI half of patients were suffering from their first episode of depression.

**Table 1:** Baseline characteristics of immediate and delayed intervention groups and observational group. Numbers indicate means ± SD (number of available data sets) or numbers/total and percentage. Statistics: One way ANOVA, X^2^, or Student´s t-test, where appropriate; *) significant

	**Immediate intervention (*n =* 56)**	**Delayed intervention (*n =* 99)**	**Observational group (*n =* 91)**	**Statistical evaluation**
Demographic data				
Age [years]	82.0 ± 6.2 (56)	81.9 ± 5.9 (99)	81.5 ± 7.1	df 2; *F =* 0.132; *p* = 0.877
Female gender (n/total; %)	44/56; 78.6%	80/99; 80.8%	61/91; 67.0%%	X^2^ = 5.991/*p* = 0.072
Living alone (n/total; %)	37/56; 66.1%	57/99; 57.6%		X^2^ = 3.841/*p* = 0.298
Depression				
HADS—total score	18.8 ± 7.0 (56)	18.1 ± 8.3 (99)	16.6 ± 6.2	df 2; *F =* 8.817/*p* = 0.165
HADS—severity of depression	11.6 ± 5.0 (56)	10.6 ± 4.9 (99)	10.1 ± 4.1	df 2; *F =* 1.747/*p* = 0.176
HAMD—total score	17.1 ± 5.4 (56)	16.8 ± 5.6 (99)	15.6 ± 5.6	df 2; *F =* 1.591/*p* = 0.206
Recurrent depressive episodes (n/total; %)	37/56; 66.1%	49/99; 49.5%	n.d	df 1; *F =* 3.98/*p* = 0.461
Antidepressant drug treatment (n/total; %)	45/56; 80.4%	70/99; 70.7%	n.d	X^2^ = 1.740/*p* = 0.259
Health status				
CIRS	15.8 ± 3.9 (56)	16.5 ± 3.7 (99)	n.d	df 1; *F =* 1.120/*p* = 0.29
Karnofsky Index	51.45 ± 10.6 (56)	54.25 ± 12.2 (99)	n.d	df 1; *F =* 2.084/*p* = 0.15
Functional status				
Barthel	76.96 ± 16.1 (56)	73.33 ± 21.1 (99)	73.19 ± 20.24	df 2; *F =* 0.761*p* = 0.47
IADL	4.50 ± 2.4 (56)	5.03 ± 2.3 (99)	n.d	df 1; *F =* 1.917/*p* = 0.168
Cognitive Function				
MMSE	26.07 ± 2.9 (56)	25.56 ± 2.8 (99)	n.d	df 1; *F =* 1.167/*p* = 0.282
Physical performance				
Timed Up and Go Test [s]	20.13 ± 7.1 (47)	20.07 ± 9.5 (82)	n.d.0	df 1; *F =* 0.001/*p* = 0.973
5 Chair Rise [s]	18.69 ± 7.66 (55)	24.18 ± 21.44 (99)	n.d	df 1; *F =* 2.164/*p* = 0.146
Psychological parameters				
Pearlin Mastery Scale	12.64 ± 3.0 (56)	12.90 ± 3.3 (99)	n.d	df 1; *F =* 0.232/*p* = 0.63
Social situation and activities				
SoS total score	17.23 ± 2.9 (56)	18.02 ± 2.9 (99)	n.d	df 1; *F =* 0.678/*p* = 0.413
Resource utilization				
Hospital days during last 30 days	1.85 ± 2.4 (47)	1.33 ± 1.8 (90)	n.d	*p* = 0.185
Care level at nursing Insurance	0.52 ± 0.7 (50)	0.34 ± 0.6 (87)	n.d	*p* = 0.130
Nursing home at T1 (n/total; %)	5/56; 8.9%	13/99; 13.1%	n.d	*p* = 0.43
Caregiver interview				
Caregiver burden	24.33 ± 14.3 (15)	13.71 ± 10.3 (38)	n.d.*	*p* = 0.015*
Caregiver depression PHQ-D-9	6.79 ± 7.3 (14)	2.58 ± 2.1 (38)	n.d	*p* = 0.051
General practitioner (GP) interview				
Number of contacts with GP/3 months	4.27 ± 3.2 (30)	6.92 ± 8.7 (25)	n.d	*p* = 0.157
Severity of depression: assessment by GP (VAS 1–10)	6.8 ± 1.7 (30)	5.54 ± 2.7 (26)	n.d	*p* = 0.048*

### Assessment of depressive symptoms

**Fig. 2:**
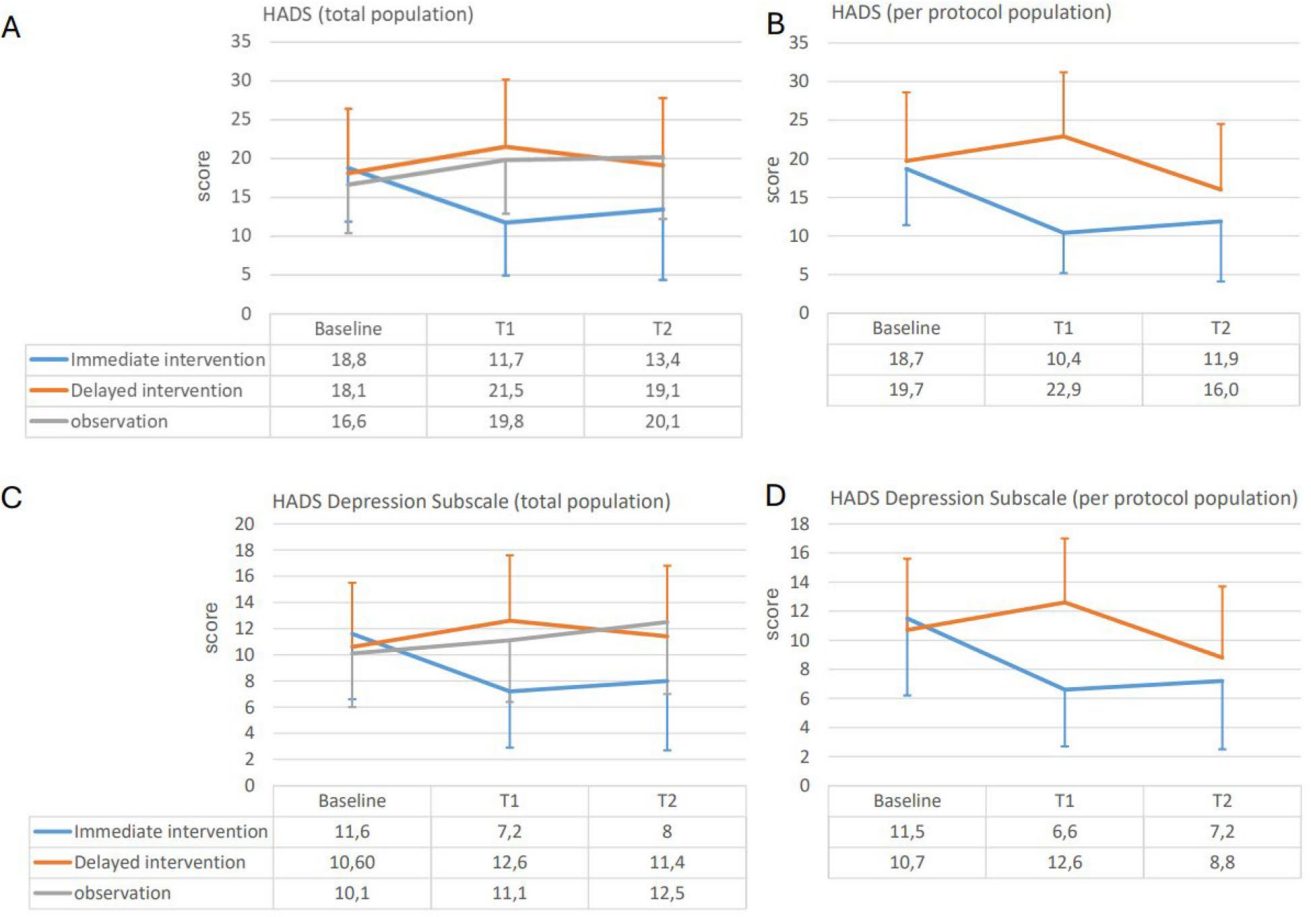
Depressive symptom scores: Hospital anxiety and depression scale (HADS) and Hamilton depression scale (HAMD): Time course from randomization (Baseline) to cross-over at 4 months (T1) and final evaluation (T2). Panel **A** HADS Intention to treat population (Last observation carried forward; ANOVA: time df 2; *F =* 0.104; *p* = 0.902; time*group interaction df 4; *F =* 23.6: *p* < 0.001). Post-hoc: immediate intervention vs. delayed intervention *p* < 0.001; immediate intervention vs. observation: *p* < 0.001; delayed intervention vs. observation: n.s., *p* = 0,891. Panel **B** HADS Per protocol population (ANOVA: time df 2; *F =* 2.888; *p* = 0.058; time*group interaction df 2; *F =* 23.5: *p* < 0.001). Panel **C** HAMD Intention to treat population (Last observation carried forward; ANOVA: time df 2; *F =* 2.888; *p* = 0.058; time*group interaction df 4; *F =* 23.49: *p* < 0.001). Post-hoc: immediate intervention vs. delayed intervention *p* = 0.001; immediate intervention vs. observation: *p* = 0.001; delayed intervention vs. observation: n.s., *p* = 0,999. Panel **D** HAMD Per protocol population (ANOVA: df 2; time *F =* 11.30; *p* < 0.001; time*group interaction df 2; *F =* 24.80: *p* < 0.001)

At baseline, subjects had depressive symptom scores corresponding to moderate depression. Depressive symptoms improved significantly and clinically relevant using HADS (Fig. 2) and HAMD (Table 2) in both groups. The same was true for HADS anxiety subscale.

**Table 2:** Additional outcome parameters: assessments of depression, anxiety, cognition and functional parameters in the per protocol population. Statistical evaluation with Two-way ANOVA: Time denotes changes in the entire study population over time, interaction Time*Group denotes differences in trajectories between groups. Numbers in brackets denote n

	**Immediate intervention**	**Delayed intervention**	**ANOVA**
Hamilton Depression Scale			
Baseline	16.5 ± 5.1 (44)	17.3 ± 6.5 (30)	Time: df 2; *F =* 11.3; *p* < 0.001 Time*Group: df 4; *F =* 24.8; *p* < 0.001
T1	9.4 ± 4.8 (44)	22.0 ± 7.0 (30)	
T2	9.5 ± 6.3 (44)	12.4 ± 7.0 (30)	
HADS Anxiety Subscale			
Baseline	7,2 ± 4.1 (44)	9.07 ± 5.3 (30)	Time: df 2; *F =* 2.08; *p* = 0.132 Time*Group: df 4; *F =* 5.5; *p* < 0.001
T1	3.8 ± 2.9 (44)	10.2 ± 4.6 (30)	
T2	4.7 ± 3.9 (44)	7.1 ± 4.8 (30)	
Body mass index [kg/m^2^]			
Baseline	28.0 ± 7.0 (45)	27.2 ± 5.5 (31)	Time: df 2; *F =* 0.007; *p* = 0.993 Time*Group: df 4; *F =* 0.587 *p* = 0.559
T1	27.8 ± 6.6 (45)	27.5 ± 5.9 (31)	
T2	27.8 ± 6.7 (45)	6.1 ± 7.2 (31)	
Barthel Index			
Baseline	76.5 ± 14.0 (44)	82.3 ± 13.1 (31)	Time: df 2; *F =* 3.075; *p* = 0.051 Time*Group: df 4; *F =* 5.568, *p* < 0.001
T1	84.0 ± 16.7 (44)	74.7 ± 19.7 (31)	
T2	79.6 ± 21.9 (44)	79.8 ± 19.2 (31)	
SF12 Total Score			
Baseline	24.2 ± 5.8 (45)	25.7 ± 6.9 (31)	Time: df 2; *F =* 14.7; *p* < 0.001 Time*Group: df 2; *F =* 35.4, *p* < 0.001
T1	32.6 ± 5.4 (45)	22.1 ± 6.1 (31)	
T2	30.8 ± 7.1 (45)	28.5 ± 7.2 (31)	
SF 12 Physical Score			
Baseline	9.8 ± 2.9 (45)	9.7 ± 3.1 (31)	Time: df 2; *F =* 5.6; *p* = 0.05 Time*Group: df 2; *F =* 12.3; *p* < 0.001
T1	12.3 ± 3.2 (45)	9.0 ± 2.6 (31)	
T2	11.3 ± 3.2 (45)	10.7 ± 3.1 (31)	
SF 12 Mental Score			
Baseline	14.4 ± 4.1 (45)	16.3 ± 4.7 (31)	Time: df 2; *F =* 14.3; *p* < 0.001 Time*Group: df 2; *F =* 34.5; *p* < 0.001
T1	20.2 ± 3.2 (45)	13.7 ± 4.2 (31)	
T2	19.5 ± 4.8 (45)	17.8 ± 4.7 (31)	
Mini Mental State Examination			
Baseline	26.5 ± 2.8 (41)	26.5 ± 2.3 (28)	Time: df 2; *F =* 9.0; *p* < 0.0001 Time*Group: df 2; *F =* 1.05; *p* = 0.356
T1	27.8 ± 2.4 (41)	26.9 ± 2.9 (28)	
T2	28.1 ± 2.9 (41)	27.5 ± 2.8 (28)	
Number of Falls			
Baseline	2.1 ± 3.3 (43)	1.8 ± 2.5 (30)	Time: df 2; *F =* 3.1; *p* < 0.055 Time*Group: df 2; *F =* 0.84, *p* = 0.434
T1	1.0 ± 2.5 (43)	1.5 ± 3.0 (30)	
T2	1.3 ± 1.9 (43)	1.5 ± 2.2 (30)	
Pearlin sense of mastery			
Baseline	12.8 ± 3.2 (45)	13.2 ± 3.3 (31)	Time: df 2; *F =* 0.543; *p* = 0.583 Time*Group: df 2; *F =* 7.4, *p* = 0.001
T1	14.4 ± 3.5 (45)	11.6 ± 3.2 (31)	
T2	13.9 ± 3.7 (45)	12.8 ± 3.3 (31)	
IADL			
Baseline	4.9 ± 2.3 (45)	5.6 ± 2.2 (31)	Time: df 2; *F =* 3.0 *p* = 0.056 Time*Group: df 2; *F =* 14.5, *p* < 0.001
T1	5.4 ± 2.5 (45)	3.9 ± 2.4 (31)	
T2	4.8 ± 2.7 (45)	5.0 ± 2.3 (31)	
Social Support (SoS)			
Baseline	17.4 ± 2.8 (45)	18.0 ± 3.3 (31)	Time: df 2; *F =* 10.3 *p* < 0.001 Time*Group: df 2; *F =* 22.2, *p* < 0.001
T1	19.9 ± 2.2 (45)	16.7 ± 3.2 (31)	
T2	19.7 ± 2.6 (45)	18.4 ± 2.8 (31)	

However, patients in II, who had received active treatment immediately after randomization, improved early and were much better after 4 months, while improvement in patients in DI was delayed and did not start prior to T1, when they started active treatment. Recovery in II was sustained after 1 year (T2), even though II patients did not receive any active treatment for the last 8 months of the study period.

Patients in the observation group, who did not receive any study treatment, got worse.

Results did not differ in a relevant fashion between intention-to-treat analysis and per-protocol analysis.

### Assessment of functional status, mobility and social situation

**Fig. 3:**
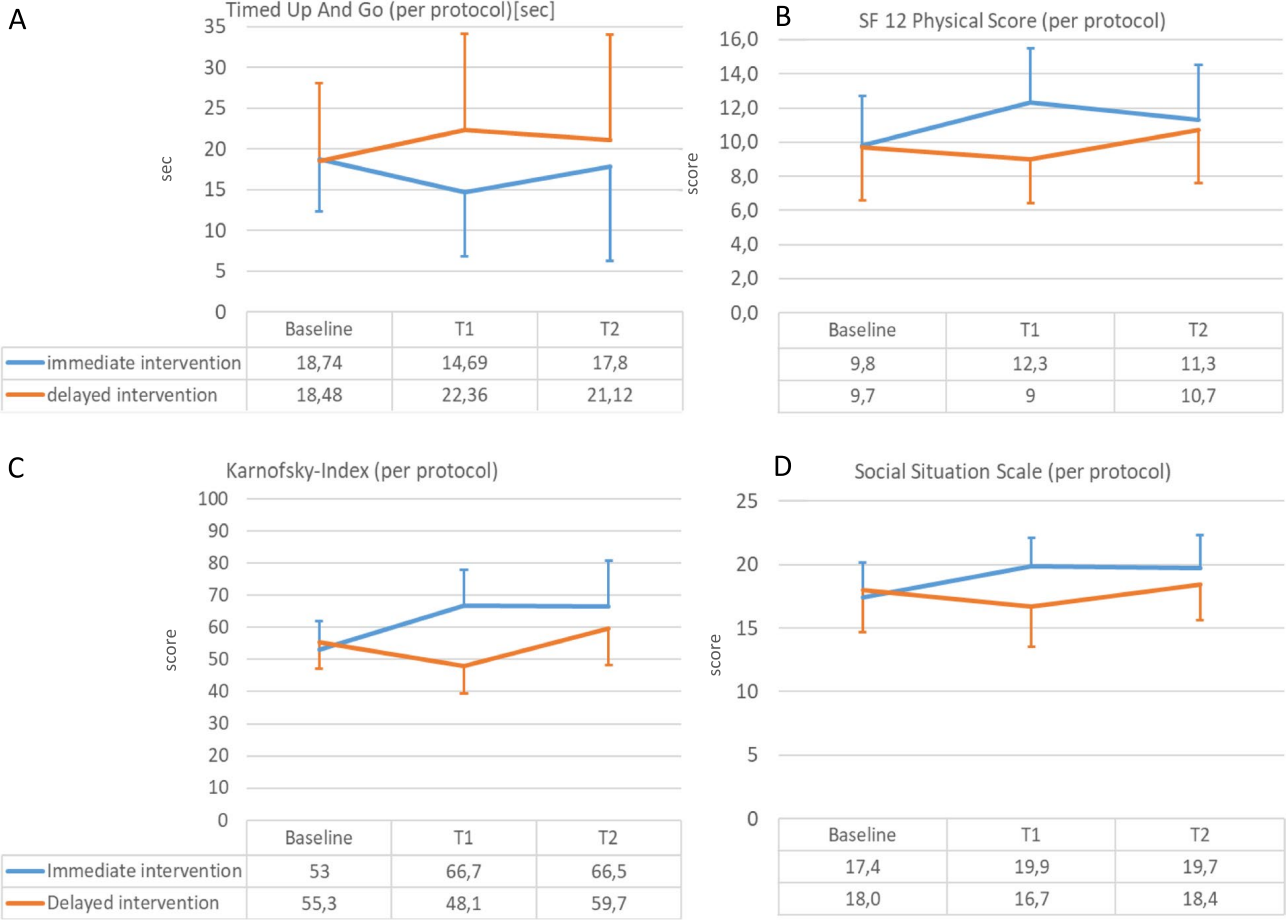
Functional parameters and social Situation: Time course from randomization (Baseline) to cross-over at 4 months (T1) and final evaluation (T2). Panel **A** Timed up and go test: Per protocol population (ANOVA: time df 2; *F =* 0.207; *p* = 0.744; time*group interaction df 2; *F =* 6.948; *p* = 0.002). Panel **B** SF 12 Questionnaire, Physical Score (ANOVA: Time: df 2; *F =* 5.6; *p* = 0.05. Time*Group: df 2; *F =* 12.3, *p* < 0.001). Panel **C** Karnovsky Index, per protocol population (ANOVA: time df 2; *F =* 10.167; *p* < 0.001; time*group interaction df 2; *F =* 32.065: *p* < 0.001). Panel **D** Social Situation Scale, per protocol population (ANOVA: time df 2; *F =* 10.3; *p* < 0.001; time*group interaction df 2; *F =* 22.2: *p* < 0.001)

Corresponding to depression scores, the trajectories of functional parameters (both, Barthel, Tab. 2, and Karnofsky Indices, Fig. 3) differed between groups. The same was for instrumental activities of daily living (Tab. 2). While in II subjects, indices remained stable throughout the whole observation period and even tended to improve during their active treatment phase between baseline and T1, they deteriorated in DI subjects between baseline and T1. They could not regain full functional competence even in the active treatment period between T1 and T2. In OG patients, only Barthel-Index was assessed. Those patients exhibited a decline in functional capacity during the study course similar to the DI (data not shown).

Mobility, as assessed by Timed-Up-and-Go (Fig. 3) improved modestly, but significantly parallel with improvement of depressive symptoms (Fig. 3). In contrast, five chair rise test and grip strength did not show any group differences (data not shown).

Changes in symptom scores such as the SF-12 [32] and various additional assessment parameters such as social situation were corresponding to changes in depression scores and significantly differed between groups (Table 2, Fig. 3).

Cognition, as assessed by Mini Mental State Examination, did not change in a clinically relevant fashion in any group.

### Nursing home admission, survival

Two patients in the immediate intervention group were newly admitted to nursing home, compared to 10 DI patients (3.6 versus 10.1%).

Among II patients, 4 patients deceased (7.1%), while mortality rate amounted to 15.2% (15 patients) in DI. Neither trend was statistically significant by itself, but when analyzed as a combined endpoint (non-prespecified analysis), this difference reached statistical significance (X^2^ = 4.72; *p* = 0.0297).

## Discussion

In this paper, we present evidence for the effectiveness of cognitive behavioral group psychotherapy applied in a day clinic setting to a very old and particularly vulnerable patient group.

The new results after cross-over intervention add to the information obtained from our previous data in various respects: (a) effects of psychotherapy are sustained for more than half a year after commencement of the active intervention; (b) later intervention four months after diagnosis of depression is beneficial as well, albeit it appears to be somewhat less effective; and finally (c) attrition on waiting list is substantial, mainly due to physical decline, death or loss of independent living. In addition, we present evidence that recovery from depression also resulted in stabilization or improvement of functional outcome.

While in recent years several trials with old patients have been published, our data are unique in various respects:

First, the study population is among the oldest populations ever studied in a psychotherapy group for depression. Second, the setting of a geriatric hospital as place for recruitment of depressed patients has not been investigated before. Third, this study has a special focus on evaluating the effects of depression treatment on physical and functional outcome parameters and provided evidence of improvement far beyond psychological outcome parameters.

In a recent meta-analysis of group psychotherapy studies for geriatric depression, only one out of 13 studies reported results of patients of a similar age [33]. Another meta-analysis of cognitive behavioral therapy included mostly studies in subjects in their seventh or eighth decade of life, with the highest mean age of 77,5 years [13]. Another study in subjects around the age of our study was conducted in nursing homes with nurse led interventions [34]. It was confounded by high attrition rates. Thus, no changes in depressive symptom scores could be reported.

Attrition was also a problem in our study, but we were able to compensate by a high number of included subjects. In addition, we closely monitored attrition, which tended to be considerably higher in the waiting list group prior to cross-over to active treatment. It is well conceivable that early intervention helped to avoid attrition. This was also the reason why we recruited more patients for the waiting list group than for immediate treatment. We also controlled for potential distortion of results by performing analyses of primary endpoints both in per protocol analysis and intention to treat analysis. Since both types of evaluations yielded similar results, we are confident that our findings are not confounded.

Our study recruited patients in a setting of a geriatric hospital. Hospital admission is a severe event in the life of older persons and triggers worries about health and independence. Almost 40% of older, hospitalized patients develop persistent cognitive-affective depressive symptoms, which predict increased morbidity and mortality [35]. In addition, multimorbidity is a typical feature of patients in geriatric departments. According to a recent meta-analysis multimorbidity has been associated with a considerably increased risk of depressive symptoms in old age [9]. Most studies in this meta-analysis were conducted in a community or primary care setting. In the setting of a geriatric hospital, patients differ from those in primary care in that they suffer from acute conditions such as fractures, heart failure or stroke. These conditions convey a high risk of subsequent manifestation of depression [4, 36–38]. Therefore, our study was conducted in a population that is highly vulnerable for incident depression and subsequent increased morbidity and mortality burden. In addition, recruiting depressive patients for a psychotherapy study in the setting of a geriatric hospital is likely to identify a high number of acute depressive episodes with a less chronic course. Therefore, it may also represent a study population, in which acute intervention is more promising than in settings where chronic depression is more prevalent. In line with these considerations is our finding that in intention-to-treat analysis of delayed treatment did not have as favorable results as immediate treatment.

In our population, we found a considerable overlap between depressive and anxiety symptoms. This is why we chose the Hospital Anxiety and Depression Scale for evaluation of the primary endpoint. Nevertheless, separate subscale analyses for depression and anxiety and analysis of depressive symptoms with the more widely used Hamilton Depression Scale yielded similar results, indicating that both, depressive symptoms and anxiety drove the outcome, not only concerns linked to hospital admission.

The overlap between depression, dementia, functional decline and mortality has been well recognized [6, 35, 39]. Even in younger subjects, major depression increases mortality not only because of higher suicide rates, but also secondary to increased mortality rates from somatic illness [40]. However, there is only sparse evidence that effective treatment of depression can alleviate its increased somatic morbidity and mortality burden. Collaborative care approaches appear to be promising both in reducing depressive symptoms and cardiac outcomes [41]. In a recent study with primary care patients 60 years or older with moderate physical comorbidity, mortality could be reduced by collaborative care for depression [42]. For many older people, functional independence is a more important goal than an extended life span. Despite the well-established association of depression with functional disability and its impact on quality of life in older persons, the effect of antidepressant treatment on functional outcome is rather unknown. Results from our study strongly suggest that CBT is not only effective in reducing depressive symptoms, but also helps to maintain functional performance in a highly vulnerable population of very old multimorbid patients. Various well-established assessment instruments for physical performance and functional independence have been consistently improved in the intervention group compared to waiting list. After cross-over, patients that had been on waiting list also benefitted from the group therapy intervention.

This study was not powered to pick up mortality differences between groups nor were mortality analyses part of the pre-specified outcome variables. Nonetheless, we observed consistent trends in improvement of nursing home admission and mortality rates. Our explorative observation of a significant reduction of the combined endpoint of nursing home admission and death may encourage further studies to investigate these clinically meaningful endpoints.

This study has several limitations: First, the control condition is a waiting list condition rather than active treatment. Adding to that limitation, the active intervention was delivered in the context of a day clinic setting, where patients were picked up at home, had lunch together and received an additional session of physical therapy or ergotherapy. As loneliness is a common complaint in frail older patients [43], our study cannot finally resolve the question if the specific CBT intervention or non-specific effects associated with the study setting are responsible for the beneficial treatment effects [44]. But the beneficial and persistent effects of an immediate psychotherapeutic intervention compared to a delayed intervention remains untouched by this limitation. As treatment options are currently limited for vulnerable home-living older adults [45], a day-clinic based approach which includes transportation may fill in a gap. Thus, our study stresses the importance of a multimodal approach including CBT in treatment of late life depression in frail patients [46].

Another limitation is the fact that inclusion was not limited to major depression, but also to less severe depressions that may be more responsive to non-specific interventions. On the other hand, geriatric depression is characterized by a more heterogeneous presentation with less severe affective symptoms and a higher prevalence of less characteristic depressive and somatic symptoms [47]. The prognostic relevance of subsyndromal depression in late life has recently been underlined [48]. Our data obtained in the observational group show that even milder forms of depression in late life are highly persistent in the absence of an intervention. We report even higher rates of persistent disease than a previous report on the natural course of depression in old age [49]. In contrast to the population based study by Luppa and colleagues, our study subjects were recruited in the context of acute physical impairment and impaired functional status, which appear to be risk factors for an unfavorable course of depression. In fact, in the study of Luppa, a substantial part of the population with low functional status was lost to follow-up.

Most of our patients did not experience complete remission, but continued to suffer from residual symptoms. In the time since we have designed the study and developed the psychotherapy manual in 2009, considerable progress in psychotherapy methods has been reported by various authors. Our promising results encourage future research with improved methods. Additional issues are open for further research: Can recovery from depression in old age improve independent living, survival and cognition? What is the specific effect of CBT, what part of the positive effects are attributable to social encounter and physical activity in the once weekly day-care setting? How to improve the diagnostic rate of depression and the availability and accessibility of psychotherapy in health care systems? These questions need to be addressed in future studies with in larger sample sizes.

## Conclusions and implications

Cognitive behavioral group therapy, embedded in a multimodal approach, is highly effective in reducing depressive symptoms even in vulnerable geriatric patients with acute medical illness. Intervention does not only effectively target affective symptoms of depression, but also helps to stabilize or improve physical function and functional independence. This improvement is sustained even upon completion of active psychotherapy. In the context of hospitalization for physical illness, early intervention is crucial. Diagnosis and treatment initiation should be initiated during acute hospitalization and extended into post-discharge ambulatory care.

## Supplementary Information

Supplementary Material 1.

Supplementary Material 2.

Supplementary Material 3.

## Data Availability

The participants gave informed consent for anonymized data sharing with low risk of identification. Raw data can be accessed in supplementary materials.
