# Supplementary material for: AIDElong—acute illness and depression in elderly: sustained improvement after group psychotherapy in geriatric patients, a follow-up of longterm effects in a randomized controlled trial
Source: BMC Geriatr. 2026 Feb 7;26:329. doi: 10.1186/s12877-026-06983-0 (PMC12983662; doi:10.1186/s12877-026-06983-0)
Supplement: Supplementary file 1 — Supplementary Material 1. [file 12877_2026_6983_MOESM1_ESM.pdf]

Code	Gruppe	Verlauf	Grund dropout	dropSitzungzahl	dropVerlauf	Gestorben zu T2
S01	10	abgeschlossen				
S02	10	abgeschlossen				
S04	10	abgeschlossen				
S05	10	abgeschlossen				
S06	10	abgeschlossen				
S07	10	abgeschlossen				
S08	10	abgeschlossen				
S09	10	abgeschlossen				
S10	10	abgeschlossen				
S13	10	abgeschlossen				
S15	10	abgeschlossen				
S16	10	abgeschlossen				
S17	10	abgeschlossen				
S19	10	abgeschlossen				
S20	10	abgeschlossen				
S21	10	abgeschlossen				
S22	10	abgeschlossen				
S23	10	abgeschlossen				
S24	10	abgeschlossen				
S25	10	abgeschlossen				
S27	10	abgeschlossen				
S28	10	abgeschlossen				
S29	10	abgeschlossen				
S30	10	abgeschlossen				
S31	10	Ther beendet				
S32	10	abgeschlossen				
S33	10	Ther beendet				
S34	10	Ther beendet				
S35	10	Ther beendet				
S37	10	Ther beendet				
S38	10	Ther beendet				
S39	10	Ther beendet				
S40	10	Ther beendet				
S41	10	Ther beendet				
S42	10	Ther beendet				
S45	10	Ther beendet				
S46	10	Ther beendet				
S47	10	Ther beendet				
S49	10	Ther beendet				
S50	10	Ther beendet				
S51	10	Ther beendet				
S53	10	Ther beendet				
S54	10	Ther beendet				
S55	10	Ther beendet				
S56	10	Ther beendet				
S03	11	dropout nach	Ther beendet		gestorben nach T2 nach Ther	

S12	11	dropout nach	Ther beendet		gestorben vor T3nach therap
S36	11	Ther beendet			
S11	12	dropout	Partnerschaft	4	abgeschlossen
S18	12	dropout	Transport	1	abgeschlossen
S26	12	dropout	Transport	7	
S43	12	dropout	zu anstrengen	6	
S44	12	dropout	möchte nicht	2	
S48	12	dropout	Antrieb	7	
S14	14	dropout	AZ	3	gestorben nach E
S52	14	dropout	gestorben vor	3	x
K04	20	abgeschlossen			
K13	20	abgeschlossen			
K15	20	abgeschlossen			
K16	20	abgeschlossen			
K21	20	abgeschlossen			
K24	20	abgeschlossen			
K25	20	abgeschlossen			
K29	20	abgeschlossen			
K33	20	abgeschlossen			
K36	20	abgeschlossen			
K37	20	abgeschlossen			
K49	20	dropout	Schwäche	3	
K51	20	Ther beendet			
K53	20	Ther beendet			
K55	20	Ther beendet			
K58	20	abgeschlossen			
K60	20	Ther beendet			
K67	20	Ther beendet			
K68	20	abgeschlossen			
K73	20	Ther beendet			
K75	20	Ther beendet			
K78	20	Ther beendet			
K79	20	Ther beendet			
K82	20	Ther beendet			
K88	20	Ther beendet			
K89	20				
K90	20	Ther beendet			
K97	20	Ther beendet			
K38	20	Ther beendet	zu anstrengen	4	
K70	20	?			
K86	20	Ther			
K07	21	dropout	Ther beendet	15	gestorben vor t3
K02	22	dropout	keine Gruppe	0	abgeschlossen
K05	22	dropout	keine Gruppe	0	abgeschlossen
K10	22	dropout	kranke Leute i	5	abgeschlossen
K11	22	dropout	Tk zu anstreng	1	abgeschlossen
K14	22	dropout	Antrieb	0	abgeschlossen
K17	22	dropout	Transport	1	abgeschlossen
K20	22	dropout	möchte nur zu	0	abgeschlossen
K22	22	dropout	fühlt sich gut	1	abgeschlossen

K23	22	dropout	keine Gruppe	0
K26	22	dropout	möchte keiner	0
K27	22	dropout	AZ, veriwrrt	1
K28	22	dropout	möchte nur st	1
K30	22	dropout	möchte neu b	0
K31	22	dropout	geht besser	0
K32	22	dropout	Antrieb	2 abgeschlossen
K34	22	dropout	Antrieb	6 abgeschlossen
K35	22	dropout	Antrieb, Fußul	2
K39	22	dropout	Kognition, zu v	1 abgeschlossen
K40	22	dropout	Familie dageg	2
K42	22	dropout	nicht nötig	1
K44	22	dropout	besucht Tk im	2
K45	22	dropout	Antrieb	1
K46	22	dropout	Antrieb, habe	0 abgeschlossen
K47	22	dropout		1
K48	22	dropout	Transport	2
K50	22	dropout	Antrieb	4
K52	22	dropout	Erschöpfung	0
K54	22	dropout	möchte nicht	0
K56	22	dropout	möchte nicht	0
K57	22	dropout	AZ	1
K61	22	dropout	nichts mehr zu	1
K63	22	dropout	keine Zeit	0
K65	22	dropout	Morgentief	1
K66	22	dropout	soz Rückzug	1
K74	22	dropout	fühlt sich bess	2
K76	22	dropout	möchte krank	3
K77	22	dropout	Belastbarkeit,	1
K80	22	dropout	antrieb abbau	
K81	22	dropout	zu anstrengen	2
K83	22	dropout	geht besser	0
K84	22	dropout	AZ	
K87	22	dropout	möchte nur st	2
K91	22	dropout		
K92	22	dropout	Antrieb	1
K93	22	dropout	inkontinenz	
K95	22	dropout		
K98	22	dropout	apoplex,hypak	3
K99	22	dropout	hypakusis, angebote im heim gut	
K94	23	?		
K41	23	dropout	Antrieb	2
K62	23	?		
K03	23	dropout	Transport	0 gestorben vor t3
K08	23	dropout	AZ, Antrieb	0 gestorben vor t3
K09	23	dropout	keine Hoffnun	0 gestorben vor t3
K12	23	dropout	Transport	0 abgeschlossen
K59	23	dropout	gestorben nac	0 x
K64	23	dropout	AZ	4 gestorben vor t3
K01	24	dropout	gestorben vor	0 x

K06	24	dropout	gestorben vor	0	x	
K18	24	dropout	gestorben vor	0	x	
K19	24	dropout	gestorben nac	0	x	
K43	24	dropout	gestorebn vor	1	x	
K69	24	dropout	gestorben vor	1	x	
K71	24	dropout	gestorben vor	2	x	
K72	24	dropout	gestorben vor	1	x	
K85	24	dropout	gestorben vor	1	x	
K96	24	dropout	gestorben			

B01		fertig				0
B05		fertig				0
B06		fertig				0
B10		fertig				0
B14		fertig				0
B16		fertig				0
B17		fertig				0
B18		fertig				0
B20		fertig				0
B23		fertig				0
B24		fertig				0
B25		fertig				0
B28		fertig				0
B30		fertig				0
B31		fertig				0
B33		fertig				0
B35		fertig				0
B38		fertig				0
						0
						0
						0
B04		(fertig)				0
B09		(fertig)				0
B13		(fertig)				0
B19		(fertig)				0
B22		(fertig)				0
B29		(fertig)				0
B32		(fertig)				0
B34		(fertig)				0
B36 / S20		(fertig)	AZ	1		0
B37		(fertig)				0
B26		fertigdropout	gestorben			0
B40						0

				0
				1
				0
				0
				0
				0
	dropout	gestorben	0	1
				1
				0
				0
				0
				0
				0
				0
				0
				0
				0
				0
				0
				0
				0
				0
				0
				0
				0
				0
				0
				0
				0
				0
				0
				0
				0
				0
				0
				0
				0
				0
				0
				0
				0
				0
				1
				0
				0
B07	dropout	gestorben	0	1
B08	dropout	gestorben	0	1
B11	dropout	gestorben	0	1
B15	dropout	gestorben	0	1
B21	dropout	gestorben	0	1

B	fühlt sich zu al	2	1
			1
			0
			0
			0
			0
			0
			0

Gestorben zu T3	Gestorben zw 1.und 2. J	gestorben gesamt	Gesamtzahl Sitzungen	Geschlecht	Alter	BMI1
	0	0	16	0	85	21
	0	0	14	1	79	26
	0	0	15	0	76	26
	0	0	16	1	88	29
			14	1	71	27
	0	0	17	1	76	32
	0	0	12	0	83	26
	0	0	13	1	79	21
	0	0	17	1	86	32
	0	0	16	1	78	27
	0	0	16	1	90	26
	1	1	17	1	71	15
	0	0	16	1	81	48
			16	1	80	31
	0	0	15	1	83	23
	0	0	16	1	84	34
	0		15	1	72	33
			11	1	67	27
	0	0	15	1	81	27
	0	0	15	1	85	39
	0	0	6	1	87	20
			17	1	79	23
	0		17	0	85	33
			17	0	89	23
			17	1	70	26
	0		17	1	82	22
	0	0	2	1	75	41
	0	0	15	1	87	25
	0		15	0	87	27
			17	0	94	28
	0		17	1	72	35
			17	0	83	23
	0	0	13	1	79	30
	0	0	15	1	83	39
	0	0	15	1	83	25
			16	1	80	26
			8	1	96	16
			15	1	82	20
			11	1	84	47
			17	1	83	30
	0	0	15	0	81	26
			16	1	76	23
	0	0	15	1	73	32
	0	0	10	1	83	29
	0	0	13	1	88	22
:apie	0	1	10	1	85	32

ɔie	0	1	16	0	79	28
	1	1	10	1	84	28
	0	0	5	1	82	27
			3	1	83	17
	0	0	7	1	85	29
			7	1	77	36
			3	0	95	26
	0	0	7	1	85	26
	0	1	3	0	85	21
	0	1	6	1	93	25
			16	1	82	27
	0	0	17	1	82	20
	0	0	16	1	85	30
	0	0	17	1	82	35
	0		15	1	76	25
			10	1	84	28
			18	1	82	20
	0		15	0	76	35
	0	0	16	1	86	29
	0	0	16	0	84	26
	0	0	16	0	78	25
			10	1	75	20
	0		17	1	85	38
			14	1	83	28
	0	0	14	1	77	32
	1	1	10	1	78	32
			14	1	78	30
	1	1	16	1	86	27
	0	0	15	1	80	26
	1	1	11	1	89	16
			6	0	78	37
	0	0	15	0	91	24
	0	0	16	1	82	32
			7	0	83	31
			17	1	84	26
			7	1	78	23
			10	1	79	25
			12	0	69	28
			6	1	79	19
	0		12	1	86	30
	0	0	12	1	73	20
	0	1	16	1	84	20
	0	0	0	1	92	20
	0	0	3	1	88	16
	0	0	5	1	79	24
			1	1	90	18
			0	1	71	32
	0	0	2	1	85	25
			2	1	85	19
			1	1	76	25

0	0	1	0	87	21
0	0	2	1	80	26
0	0	1	1	91	21
0	0	2	1	89	20
0	0	0	1	83	19
0	0	4	1	82	28
0		2	1	86	30
		7	1	81	30
0	0	2	1	88	23
		1	1	83	29
0	0	2	1	86	28
0	0	1	1	66	28
		2	1	75	24
		1	0	80	25
1	0	0	1	83	24
		0	1	85	27
0		2	1	76	40
		6	1	83	33
0		1	1	71	22
0	0	0	1	89	24
		1	1	89	24
1	0	2	1	85	23
		1	0	85	29
		1	1	77	18
		1	1	86	24
		1	1	87	20
		2	1	83	36
		3	1	86	30
		1	1	69	22
		1	0	89	20
0	0	2	1	81	19
		0	1	90	19
1	1	2	0	81	24
		2	1	92	30
		4	1	76	30
		1	1	84	36
		2	1	84	24
		1	1	76	27
		7	0	83	27
		6	1	90	26
0	1	3	0	64	22
0	1	2	1	81	17
1	1	1	1	80	23
0	1	0	0	87	20
0	1	2	1	83	20
0	1	6	1	80	20
0	1	5	1	72	33
0	1	0	1	90	24
0	1	7	0	78	25
0	1	0	1	80	39

0	1	2	1	87	18
0	1	0	0	77	20
0	1	7	0	88	24
0	1	1	0	80	30
0	1	1	1	91	24
0	1	2	1	71	23
0	1	1	1	83	31
0	1	1	1	82	28
0	1	3	1	81	34

				Geschlecht	Alter bei erstkontakt
0	0	0		1	85
				0	84
0				1	86
0				0	86
0				1	81
0				1	80
0				0	83
1				1	79
0	0	0		1	83
0				1	87
				1	67
0				1	86
1				1	87
0	1	1		1	75
0				1	80
0				1	82
0				1	78
0				1	88
0				1	97
0	0	0		1	93
0	0	0		0	81
1				0	90
0				1	84
0				1	81
				0	75
				1	83
0				1	80
1				0	86
0				1	82
1				1	85
0				1	85
1				0	88
0				1	78

			1	84
			1	89
0			1	81
0			1	72
0			1	88
0	0	0	1	75
			1	93
			1	78
0			1	74
0			0	89
0	0	0	1	75
			1	79
0			0	88
0			1	84
0			1	69
0			0	68
0			1	95
0			1	82
0	0	0	1	81
0			0	86
1			0	68
0			1	71
0	0	0	1	71
0	0	0	0	84
0			0	87
0	0	0	0	88
0	0	0	0	71
0			0	84
0			0	69
0			1	87
0			1	96
1			0	80
0			1	95
1			1	87
0			1	77
			1	88
0			1	88
0	0	0	1	82
1			0	73
0			1	82
0			1	74
0	0	0	0	73
			0	71
0			0	77
			1	77
			0	75
			0	81
			0	79
			1	84
			0	81

				1	90
				0	82
1				1	88
0				1	80
0				1	76
0	0	0		1	76
0	0	0		0	89
0	0	0		1	61

BMI2	BMI2LOCF	BMI3	BMI3	Wohnsituation1	Wohnsituation2	Wohnsituation2LOCF
23	23	19,9	19,9	1	1	1
23	23	22,7	22,7	1	1	1
27	27	26,4	26,4	1	1	1
31	31	29,6	29,6	1	1	1
28	28	27,9	27,9	4	4	4
31	31	32,1	32,1	2	2	2
24	24	22,2	22,2	2	2	2
22	22	21,6	21,6	1	1	1
30	30	29,8	29,8	4	4	4
28	28	29,3	29,3	2	2	2
25	25	26,1	26,1	2	2	2
16	16	14,1	14,1	2	2	2
44	44	43,1	43,1	2	2	2
29	29	30,8	30,8	1	4	4
20	20	18,75	18,75	1	1	1
34	34	32,5	32,5	2	2	2
33	33	34	34	2	2	2
33	33	28,7	28,7	1	1	1
27	27	26,7	26,7	1	1	1
40	40	35,7	35,7	1	1	1
21	21	24,4	24,4	2	2	2
23	23	23,1	23,1	1	1	1
30	30	35	35	1	1	1
23	23	23,2	23,2	1	1	1
28	28	25	25	2	2	2
23	23	25,3	25,3	1	1	1
40	40	36,1	36,1	4	4	4
23	23	24,2	24,2	1	1	1
28	28	27,8	27,8	1	1	1
29	29	28,5	28,5	4	4	4
34	34	33,1	33,1	2	2	2
22	22	21	21	1	1	1
29	29	30,4	30,4	5	5	5
40	40	39,5	39,5	1	1	1
25	25	27,7	27,7	1	1	1
27	27	29	29	2	2	2
16	16	16	16	1	1	1
20	20	20,7	20,7	1	1	1
43	43	46,7	46,7	2	2	2
33	33	34,6	34,6	1	1	1
25	25	25,9	25,9	1	1	1
22	22	22,9	22,9	1	1	1
31	31	30,5	30,5	1	1	1
28	28	30,8	30,8	1	1	1
20	20	19,8	19,8	2	2	2
31	31		31	3	3	3

31	31		31	2	2	2
28	28		28	1	4	4
27	27		27	2	2	2
18	18	17,5	17,5	2	2	2
30	30	28,3	28,3	4	4	4
35	35	35,2	35,2	2	2	2
26	26	25,4	25,4	1	1	1
25	25	23,1	23,1	1	4	4
	21		21	1		1
	25		25	2		2
27	27	24,7	24,7	1	1	1
19	19	17,1	17,1	1	1	1
29	29	30,5	30,5	1	1	1
35	35	34,7	34,7	1	1	1
24	24	23,8	23,8	1	4	4
27	27	29,3	29,3	1	1	1
18	18	20	20	1	1	1
35	35	39,9	39,9	1	1	1
29	29	30,8	30,8	2	4	4
29	29	24,4	24,4	1	1	1
24	24	25,1	25,1	1	1	1
20	20	21,3	21,3	2	2	2
38	38	36,9	36,9	1	1	1
28	28	27,6	27,6	1	1	1
36	36	32,3	32,3	1	1	1
38	38	31,5	31,5	1	1	1
29	29	29,9	29,9	2	2	2
28	28	35,7	35,7	1	1	1
27	27	26,8	26,8	1	1	1
16	16	16,8	16,8	4	4	4
36	36	37,6	37,6	1	1	1
28	28	25,7	25,7	1	1	1
28	28	33,3	33,3	1	1	1
29	29	29,4	29,4	2	4	4
25	25	20,5	20,5	2	4	4
22	22	22,3	22,3	1	1	1
26	26	26,1	26,1	2	2	2
32	32	23,2	23,2	4	4	4
20	20	20,8	20,8	1	4	4
30	30	30,3	30,3	1	1	1
20	20	20	20	2	2	2
21	21		21	4	4	4
21	21	20,7	20,7	1	1	1
17	17	17,8	17,8	1	1	1
22	22	21,5	21,5	1	1	1
16	16	14	14	1	4	4
31	31	43,1	43,1	4	4	4
28	28	32,4	32,4	1	1	1
23	23	20,9	20,9	1	1	1
35	35	33,3	33,3	2	2	2

21	21	21,1	21,1	2	2	2
25	25	26	26	1	1	1
19	19	17,9	17,9	2	2	2
18	18	18,3	18,3	1	4	4
21	21	24,2	24,2	2	2	2
29	29	32,3	32,3	1	4	4
30	30	32,1	32,1	1	1	1
30	30	28,1	28,1	1	0	0
24	24	22,9	22,9	1	1	1
31	31	30,9	30,9	2	2	2
28	28	27,3	27,3	2	2	2
29	29	29,7	29,7	1	1	1
24	24	23,8	23,8	4	4	4
20	20	24,4	24,4	1	1	1
25	25	26,7	26,7	1	1	1
25	25	24,5	24,5	4	4	4
38	38	39,5	39,5	1	1	1
32	32	32,4	32,4	2	2	2
22	22	23	23	1	1	1
24	24	23,2	23,2	2	2	2
23	23	28,1	28,1	1	1	1
18	18	17,4	17,4	2	2	2
26	26	29,9	29,9	1	4	4
22	22	21,6	21,6	1	1	1
23	23	20,4	20,4	4	4	4
24	24	23,6	23,6	4	4	4
37	37	36,9	36,9	1	1	1
30	30	29,4	29,4	1	4	4
21	21	24	24	4	4	4
19	19	18,4	18,4	1	1	1
21	21	19,9	19,9	1	1	1
18	18	17,7	17,7	1	4	4
23	23	22,6	22,6	2	2	2
26	26	23,1	23,1	2	2	2
29	29	30,1	30,1	2	2	2
34	34	33,4	33,4	1	1	1
24	24	24	24	1	4	4
26	26	25,6	25,6	4	4	4
28	28	26,5	26,5	1	1	1
25	25	24,5	24,5	2	4	4
21	21		21	1	1	1
15	15		15	1	1	1
24	24		24	1	1	1
21	21		21	4	4	4
19	19		19	1	4	4
19	19		19	2	2	2
34	34		34	1	1	1
22	22		22	1	1	1
25	25		25	1	1	1
	39		39	1		1

18	18	2	2
20	20	1	1
24	24	4	4
30	30	2	2
24	24	2	2
23	23	1	1
31	31	1	1
28	28	4	4
34	34	2	2

Wohnsituation3	Wohnsituation3LOCF	Pflegeheim1	HADS 1	HADS 2	HADS 2LOCF	HADS 3
1	1	0	24	14	14	12
1	1	0	20	11	11	14
1	1	0	24	9	9	4
1	1	0	17	6	6	17
4	4	1	20	9	9	10
2	2	0	23	16	16	16
2	2	0	14	13	13	14
1	1	0	22	16	16	19
4	4	1	12	8	8	7
2	2	0	9	4	4	4
2	2	0	19	6	6	4
2	2	0	12	9	9	31
2	2	0	14	15	15	18
1	1	0	22	18	18	12
1	1	0	23	5	5	25
2	2	0	20	20	20	19
2	2	0	9	2	2	7
1	1	0	33	4	4	3
1	1	0	10	9	9	9
1	1	0	24	18	18	26
2	2	0	9	3	3	1
1	1	0	13	7	7	5
1	1	0	8	6	6	5
1	1	0	19	10	10	4
2	2	0	24	12	12	9
1	1	0	27	2	2	4
4	4	1	29	9	9	5
1	1	0	8	5	5	3
1	1	0	28	11	11	26
4	4	1	23	16	16	10
2	2	0	29	1	1	
1	1	0	21	10	10	12
5	5	0	22	5	5	9
1	1	0	16	15	15	17
1	1	0	23	7	7	14
2	2	0	6	4	4	5
1	1	0	21	17	17	19
1	1	0	21	17	17	19
2	2	0	8	5	5	7
1	1	0	33	18	18	30
1	1	0	8	10	10	8
1	1	0	13	12	12	2
1	1	0	28	20	20	19
1	1	0	27	9	9	10
2	2	0	15	16	16	11
	3	0	23	29	29	

	2	0	18	6	6	
	4	0	20	2	2	
2	2	0	14	25	25	26
2	2	0	21	19	19	18
4	4	1	30	25	25	38
2	2	0	19	10	10	14
1	1	0	13	22	22	25
4	4	0	8	21	21	29
	1	0	22		22	
	2	0	15		15	
4	4	0	9	18	18	17
1	1	0	23	31	31	17
1	1	0	9	9	9	7
1	1	0	17	24	24	22
1	1	0	17	13	13	17
1	1	0	26	35	35	30
1	1	0	26	16	16	14
1	1	0	10	7	7	4
4	4	0	30	29	29	7
1	1	0	12	22	22	16
1	1	0	25	29	29	21
2	2	0	21	28	28	17
1	1	0	19	31	31	27
1	1	0	18	33	33	15
1	1	0	13	16	16	
1	1	0	12	16	16	15
2	2	0	10	17	17	12
2	2	0	11	18	18	2
1	1	0	32	32	32	30
4	4	1	24	37	37	8
1	1	0	23	32	32	19
1	1	0	8	12	12	4
1	1	0	15	22	22	23
4	4	0	10	12	12	7
4	4	0	8	19	19	6
1	1	0	21	24	24	14
4	4	0	25	27	27	17
4	4	1	21	18	18	21
4	4	0	38	17	17	30
1	1	0	36	32	32	32
2	2	0	35	26	26	8
	4	1	9	18	18	
1	1	0	19	14	14	18
1	1	0	37	33	33	26
1	1	0	29	32	32	36
1	1	0	12	12	12	34
4	4	1	11	10	10	14
1	1	0	11	17	17	18
1	1	0	13	13	13	18
2	2	0	26	13	13	20

2	2	0	25	28	28	24
1	1	0	17	23	23	18
4	4	0	22	34	34	33
4	4	0	21	23	23	24
2	2	0	8	15	15	14
1	1	0	15	13	13	11
1	1	0	23	34	34	33
1	1	0	27	30	30	37
1	1	0	20	18	18	21
4	4	0	28	16	16	15
4	4	0	10	16	16	11
1	1	0	8	8	8	8
4	4	1	11	11	11	8
1	1	0	15	23	23	28
1	1	0	25	31	31	17
4	4	1	18	27	27	22
1	1	0	11	14	14	9
2	2	0	18	31	31	35
1	1	0	15	19	19	25
2	2	0	17	27	27	18
4	4	0	15	19	19	12
4	4	0	25	39	39	26
4	4	0	19	21	21	14
1	1	0	34	15	15	14
4	4	1	9	19	19	19
4	4	1	14	16	16	17
1	1	0	13	9	9	12
4	4	0	9	35	35	37
4	4	1	8	21	21	16
1	1	0	25	31	31	37
1	1	0	19	28	28	20
4	4	0	8	11	11	18
2	2	0	24	37	37	17
2	2	0	15	18	18	24
1	1	0	14	25	25	21
1	1	0	22	30	30	15
4	4	0	25	8	8	9
4	4	1	9	18	18	21
1	1	0	12	16	16	25
4	4	0	13	18	18	22
	1	0	5	15	15	
	1	0	33	38	38	
	1	0	11	24	24	
	4	1	9	13	13	
	4	0	19	24	24	
	2	0	9	11	11	
	1	0	8	13	13	
	1	0	21	33	33	
	1	0	11	10	10	
	1	0	17		17	

2	0	17	17
1	0	25	25
4	1	33	33
2	0	8	8
2	0	20	20
1	0	34	34
1	0	33	33
4	1	11	11
2	0	10	10

HADS 1 gesamt je 0-3 Punkte wie an Rand	HADS 2 gesamt je 0-3 Punkte wie an Rand	HADS 2 gesamt je 0-3 Punkte wie an Rand	HADS 3 gesamt je 0-3 Punkte wie an Rand
16	8	8	12
10	23	23	
15	25	25	23
9	14	14	26
23	29	29	31
9	21	21	27
14	21	21	22
19	13	13	
10	10	10	15
15	21	21	30
20	26	26	
17	21	21	21
13	18	18	
20	19	19	25
26	22	22	32
12	14	14	11
10	22	22	21
12	18	18	13
10	12	12	12
23	14	14	40
13	22	22	25
18	31	31	
16	19	19	21
9	24	24	21
25	33	33	
18	23	23	
8	8	8	11
29	38	38	
8	15	15	18
29	37	37	
16	14	14	21
15	39	39	
12	16	16	14

		16	16	16	
		21		21	
		16	19	19	19
		16	14	14	13
		16	16	16	14
		23	25	25	36
		21		21	
		13		13	
		9	10	10	12
		14	14	14	22
		19	19	19	13
		11	30	30	
		15	15	15	13
		14	17	17	15
		21	22	22	22
		17	19	19	23
		25	25	25	18
		12	15	15	13
		17	19	19	13
		23	22	22	15
		10	15	15	
		29	19	19	15
		10	9	9	4
		11	14	14	13
		26	34	34	34
		23	26	26	28
		31	19	19	31
		22	30	30	28
		15	30	30	21
		9	14	14	15
		15	19	19	25
2	2	33	27	27	
		8	10	10	10
		17	21	21	
		11	15	15	35
		14	24	24	
		10	25	25	26
		10	13	13	11
		13	11	11	
		10	12	12	30
		28	18	18	18
		15	18	18	24
		14		14	
		13	15	15	15
		23	24	24	
		13		13	
		15		15	
		21		21	
		23		23	
		11		11	

14		14	
22		22	
21	28	28	
13	19	19	19
10	27	27	27
21	21	21	14
33	35	35	33
16	17	17	15

HADS 3LOCF	HADS1-Depr	HADS2-Depr	HADS2-DeprLOCF	HADS3-Depr	HADS3-DeprLOCF	HADS1-Angst
12	15	9	9	8	8	9
14	10	5	5	6	6	10
4	15	5	5	3	3	9
17	6	1	1	9	9	11
10	17	7	7	9	9	3
16	12	9	9	8	8	11
14	6	10	10	10	10	8
19	13	11	11	12	12	9
7	8	7	7	5	5	4
4	6	1	1	2	2	3
4	12	2	2	2	2	7
31	8	6	6	20	20	4
18	6	5	5	7	7	8
12	18	17	17	10	10	4
25	17	2	2	14	14	6
19	17	12	12	14	14	3
7	2	1	1	2	2	7
3	21	4	4	3	3	12
9	5	6	6	4	4	5
26	12	11	11	15	15	12
1	4	1	1	1	1	5
5	4	3	3	2	2	9
5	5	5	5	3	3	3
4	16	8	8	4	4	3
9	13	7	7	6	6	11
4	20	1	1	1	1	7
5	14	1	1	1	1	15
3	5	5	5	3	3	3
26	16	8	8	12	12	12
10	20	14	14	10	10	3
1	15	0	0		0	14
12	12	9	9	8	8	9
9	11	3	3	6	6	11
17	12	11	11	8	8	4
14	18	7	7	10	10	5
5	4	4	4	3	3	2
19	16	14	14	14	14	5
19	7	9	9	10	10	14
7	6	4	4	3	3	2
30	14	9	9	16	16	19
8	8	7	7	7	7	0
2	8	6	6	1	1	5
19	18	9	9	12	12	10
10	16	5	5	8	8	11
11	12	9	9	6	6	3
29	13	14	14		14	10

6	11	5	5		5	7
2	9	2	2		2	11
26	6	10	10	12	12	8
18	17	13	13	13	13	4
38	19	12	12	21	21	11
14	11	4	4	3	3	8
25	13	16	16	16	16	0
29	6	13	13	17	17	2
22	12		12		12	10
15	12		12		12	3
17	3	11	11	7	7	6
17	13	20	20	10	10	10
7	5	5	5	5	5	4
22	11	17	17	14	14	6
17	10	7	7	6	6	7
30	9	14	14	14	14	17
14	8	4	4	7	7	17
4	7	4	4	1	1	3
7	16	17	17	6	6	14
16	9	9	9	7	7	3
21	15	17	17	8	8	10
17	10	15	15	9	9	11
27	15	18	18	14	14	4
15	9	16	16	8	8	9
16	4	11	11		11	9
15	6	8	8	6	6	6
12	5	11	11	6	6	5
2	4	12	12	2	2	7
30	16	16	16	15	15	16
8	15	19	19	5	5	9
19	15	17	17	14	14	8
4	3	4	4	0	0	5
23	7	14	14	14	14	8
7	9	8	8	7	7	1
6	3	8	8	3	3	5
14	15	13	13	13	13	6
17	15	20	20	14	14	10
21	15	13	13	11	11	6
30	17	11	11	14	14	21
32	19	16	16	20	20	17
8	16	15	15	5	5	19
18	8	12	12		12	1
18	11	8	8	6	6	8
26	19	18	18	19	19	8
36	17	16	16	20	20	12
34	7	8	8	21	21	5
14	6	8	8	8	8	5
18	7	9	9	10	10	4
18	7	7	7	11	11	6
20	18	13	13	16	16	8

24	14	16	16	18	18	11
18	14	20	20	9	9	3
33	10	17	17	20	20	12
24	13	18	18	19	19	8
14	5	6	6	10	10	3
11	10	7	7	7	7	5
33	15	20	20	20	20	8
37	12	17	17	21	21	15
21	12	11	11	13	13	8
15	18	13	13	12	12	10
11	2	9	9	7	7	8
8	1	1	1	1	1	7
8	5	6	6	4	4	6
28	10	13	13	17	17	5
17	15	17	17	14	14	10
22	9	14	14	15	15	9
9	5	9	9	6	6	6
35	10	18	18	20	20	8
25	8	11	11	11	11	7
18	13	18	18	14	14	4
12	6	15	15	8	8	9
26	16	21	21	16	16	9
14	14	14	14	12	12	5
14	17	11	11	6	6	17
19	6	14	14	14	14	3
17	13	13	13	13	13	1
12	5	2	2	4	4	8
37	3	19	19	20	20	6
16	4	13	13	10	10	4
37	16	19	19	21	21	9
20	12	16	16	13	13	7
18	7	7	7	13	13	1
17	16	20	20	10	10	8
24	12	15	15	18	18	3
21	10	14	14	12	12	4
15	14	18	18	9	9	8
9	20	4	4	5	5	5
21	9	7	7	10	10	0
25	10	9	9	15	15	2
22	9	11	11	13	13	5
15	5	12	12		12	0
38	21	21	21		21	12
24	7	15	15		15	4
13	8	8	8		8	1
24	15	17	17		17	4
11	5	9	9		9	4
13	6	9	9		9	2
33	15	21	21		21	6
10	5	5	5		5	6
17	10		10		10	7

17	9		9		9	8
25	13		13		13	12
33	20		20		20	13
8	8		8		8	0
20	9		9		9	11
34	17		17		17	17
33	18		18		18	15
11	6		6		6	5
10	7		7		7	3

HADS 3 gesamt je 0-3 Punkte wie an Rand	HADS1-Depr Summe Frage 2,4,6,8,10,12,	HADS2-Depr Summe Frage 2,4,6,8,10,12,	HADS2-Depr Summe Frage 2,4,6,8,10,12,	HADS3-Depr Summe Frage 2,4,6,8,10,12,	HADS3-Depr Summe Frage 2,4,6,8,10,12,	HADS1-Angst Summe Frage 1,3,5,7,9,11,1
12	13	8	8	10	10	3
23	9	15	15		15	1
23	8	15	15	16	16	7
26	6	8	8	15	15	3
31	13	13	13	15	15	10
27	5	15	15	16	16	4
22	10	14	14	16	16	4
13	10	8	8		8	9
15	6	4	4	8	8	4
30	12	12	12	16	16	3
26	18	21	21		21	2
21	11	16	16	13	13	6
18	5	13	13		13	8
25	12	14	14	16	16	8
32	15	12	12	20	20	11
11	5	6	6	6	6	7
21	9	9	9	11	11	1
13	5	8	8	7	7	7
12	10	9	9	9	9	0
40	9	5	5	21	21	14
25	12	15	15	18	18	1
31	10	17	17		17	8
21	8	9	9	10	10	8
21	7	15	15	11	11	2
33	15	19	19		19	10
23	15	16	16		16	3
11	0	0	0	4	4	8
38	16	19	19		19	13
18	6	10	10	11	11	2
37	14	20	20		20	15
21	7	8	8	14	14	9
39	8	21	21		21	7
14	6	12	12	8	8	6

16	12	13	13		13	4
21	15		15		15	6
19	11	12	12	12	12	5
13	9	6	6	7	7	7
14	12	13	13	9	9	4
36	17	17	17	21	21	6
21	13		13		13	8
13	6		6		6	7
12	7	4	4	5	5	2
22	6	6	6	13	13	8
13	7	7	7	6	6	12
30	11	19	19		19	0
13	11	11	11	10	10	4
15	10	11	11	10	10	4
22	12	13	13	13	13	9
23	10	10	10	13	13	7
18	14	12	12	8	8	11
13	8	9	9	8	8	4
13	10	11	11	7	7	7
15	16	15	15	11	11	7
15	8	9	9		9	2
15	18	11	11	9	9	11
4	5	4	4	1	1	5
13	5	8	8	8	8	6
34	15	21	21	21	21	11
28	15	19	19	21	21	8
31	18	13	13	18	18	13
28	16	19	19	17	17	6
21	7	18	18	12	12	8
15	6	10	10	11	11	3
25	11	13	13	17	17	4
27	18	17	17		17	15
10	7	8	8	8	8	1
21	11	13	13		13	6
35	4	6	6	20	20	7
24	11	16	16		16	3
26	6	17	17	18	18	4
11	5	6	6	5	5	5
11	8	6	6		6	5
30	7	7	7	18	18	3
18	13	7	7	7	7	15
24	8	13	13	15	15	7
14	10		10		10	4
15	9	8	8	8	8	5
24	13	15	15		15	10
13	5		5		5	8
15	8		8		8	7
21	17		17		17	4
23	13		13		13	10
11	6		6		6	5

14	7		7		7	7
22	18		18		18	4
28	5	12	12		12	16
19	11	14	14	5	5	2
27	10	16	16	17	17	0
14	13	13	13	8	8	8
33	17	19	19	19	19	16
15	6	8	8	7	7	10

HADS2-Angst	HADS2-AngstLOCF	HADS3-Angst	HADS3-AngstLOCF	HAMD 1-Gesamtscore	HAMD 2-gesamt	HAMD 2-gesamtLOCF
5	5	4	4	18	9	9
6	6	8	8	23	21	21
4	4	1	1	26	6	6
5	5	8	8	18	4	4
2	2	1	1	20	7	7
7	7	8	8	27	14	14
3	3	4	4	10	11	11
5	5	7	7	24	13	13
1	1	2	2	16	15	15
3	3	2	2	11	5	5
4	4	2	2	23	7	7
3	3	11	11	18	6	6
10	10	11	11	16	11	11
1	1	2	2	17	8	8
3	3	11	11	13	3	3
8	8	5	5	16	16	16
1	1	5	5	22	4	4
0	0	0	0	14	6	6
3	3	5	5	8	7	7
7	7	11	11	15	14	14
2	2	0	0	13	2	2
4	4	3	3	23	5	5
1	1	2	2	8	6	6
2	2	0	0	15	5	5
5	5	3	3	15	10	10
1	1	3	3	20	6	6
8	8	4	4	18	6	6
0	0	0	0	9	5	5
3	3	14	14	15	13	13
2	2	0	0	17	16	16
1	1		1	31	4	4
1	1	4	4	15	12	12
2	2	3	3	21	11	11
4	4	9	9	17	11	11
0	0	4	4	19	5	5
0	0	2	2	5	6	6
3	3	5	5	20	18	18
8	8	9	9	24	13	13
1	1	4	4	13	5	5
9	9	14	14	19	12	12
3	3	1	1	8	2	2
6	6	1	1	10	10	10
11	11	7	7	15	18	18
4	4	2	2	19	11	11
7	7	5	5	14	17	17
15	15		15	21	32	32

1	1		1	20	8	8
0	0		0	22	4	4
15	15	14	14	12	27	27
6	6	5	5	15	14	14
13	13	17	17	24	21	21
6	6	9	9	13	11	11
6	6	9	9	13	16	16
8	8	12	12	13	22	22
	10		10	20		20
	3		3	24		24
7	7	10	10	15	20	20
11	11	7	7	14	35	35
4	4	2	2	9	10	10
7	7	8	8	15	19	19
6	6	11	11	13	23	23
21	21	16	16	23	31	31
12	12	7	7	29	22	22
3	3	3	3	15	6	6
12	12	1	1	27	23	23
13	13	9	9	13	22	22
12	12	13	13	25	24	24
13	13	8	8	18	26	26
13	13	13	13	15	24	24
17	17	7	7	19	30	30
5	5		5	17	13	13
8	8	9	9	12	23	23
6	6	6	6	7	17	17
6	6	0	0	21	17	17
16	16	15	15	18	29	29
18	18	3	3	22	30	30
15	15	5	5	17	35	35
8	8	4	4	9	8	8
8	8	9	9	13	21	21
4	4	0	0	9	13	13
11	11	3	3	7	19	19
11	11	1	1	18	18	18
7	7	3	3	23	20	20
5	5	10	10	15	21	21
6	6	16	16	33	25	25
16	16	12	12	22	26	26
11	11	3	3	22	22	22
6	6		6	14	26	26
6	6	12	12	18	15	15
15	15	7	7	14	25	25
6	6	16	16	17	29	29
4	4	13	13	22	16	16
2	2	6	6	8	12	12
8	8	8	8	17	23	23
6	6	7	7	23	22	22
0	0	4	4	13	10	10

12	12	6	6	21	27	27
3	3	9	9	18	22	22
17	17	13	13	19	29	29
5	5	5	5	31	20	20
9	9	4	4	8	11	11
6	6	4	4	17	18	18
14	14	13	13	22	27	27
13	13	16	16	24	34	34
7	7	8	8	14	15	15
3	3	3	3	19	13	13
7	7	4	4	9	20	20
7	7	7	7	16	11	11
5	5	4	4	12	13	13
10	10	11	11	13	26	26
14	14	3	3	15	26	26
13	13	7	7	15	22	22
5	5		5	10	17	17
13	13	15	15	16	29	29
8	8	14	14	10	16	16
9	9	4	4	16	21	21
4	4	4	4	15	21	21
18	18	10	10	18	30	30
7	7	2	2	22	20	20
4	4	8	8	28	14	14
5	5	5	5	13	18	18
3	3	4	4	17	17	17
7	7	8	8	14	8	8
16	16	17	17	13	35	35
8	8	6	6	16	29	29
12	12	16	16	21	26	26
12	12	7	7	11	27	27
4	4	5	5	12	12	12
17	17	7	7	18	29	29
3	3	6	6	16	20	20
11	11	9	9	17	24	24
12	12	6	6	22	27	27
4	4	4	4	20	11	11
11	11	11	11	13	16	16
7	7	10	10	11	21	21
7	7	9	9	10	17	17
3	3		3	21	19	19
17	17		17	25	37	37
9	9		9	10	28	28
5	5		5	20	19	19
7	7		7	21	23	23
2	2		2	10	28	28
4	4		4	9	19	19
12	12		12	17	31	31
5	5		5	8	18	18
	7		7	17		17

8	8	13	13
12	12	20	20
13	13	27	27
0	0	12	12
11	11	14	14
17	17	27	27
15	15	22	22
5	5	20	20
3	3	17	17

HADS2-Angst Summe Frage[illegible] 1,3,5,7,9,11,1[illegible]	HADS2-Angst Summe Fragen 1,3,5,7,9,11,13	HAMD 1- Ges[illegible]	HAMD 2-gesa[illegible]	HAMD 2-gesa[illegible]
0	0	15	15	15
8	8	8	23	23
10	10	16	23	23
6	6	12	13	13
16	16	13	27	27
6	6	8	13	13
7	7	7	16	16
5	5	26	16	16
6	6	10	5	5
9	9	18	21	21
5	5	10	10	10
5	5	10	20	20
5	5	17	24	24
5	5	17	26	26
10	10	13	18	18
8	8	19	13	13
13	13	17	27	27
10	10	9	23	23
3	3	16	19	19
9	9	20	16	16
7	7	19	25	25
14	14	16	24	24
10	10	17	20	20
9	9	16	19	19
14	14	24	25	25
7	7	24	18	18
8	8	14	18	18
19	19	28	40	40
5	5	20	14	14
17	17	29	33	33
7	7	8	10	10
18	18	17	36	36
4	4	8	14	14

3	3			13	16	16
	6			10		10
7	7			20	21	21
8	8			13	11	11
3	3			10	10	10
8	8			9	17	17
	8			23		23
	7			8		8
6	6			13	17	17
8	8			11	11	11
12	12			29	31	31
11	11			7	24	24
4	4			10	14	14
6	6			12	16	16
9	9			18	22	22
9	9			13	16	16
13	13			17	19	19
6	6			9	13	13
8	8			18	18	18
7	7			12	19	19
6	6			13	21	21
8	8			15	12	12
5	5			14	13	13
6	6			15	21	21
13	13			26	41	41
5	5			18	23	23
6	6			22	15	15
11	11			22	29	29
12	12			11	23	23
4	4			12	19	19
6	6			15	20	20
10	10	10	10	24	27	27
2	2			10	13	13
8	8			19	18	18
9	9			15	16	16
8	8			14	19	19
8	8			9	21	21
7	7			18	16	16
5	5			15	15	15
6	6			8	11	11
11	11			21	21	21
5	5			13	12	12
	4			12		12
7	7			10	11	11
9	9			22	16	16
	8			13		13
	7			12		12
	4			25		25
	10			18		18
	5			20		20

	7	14		14
	4	25		25
16	16	22	26	26
5	5	14	19	19
11	11	17	27	27
8	8	18	23	23
16	16	25	27	27
9	9	8	15	15

HAMD 3-gesamt	HAMD 3-gesamtLOCF	MMSE 1	MMSE 2	MMSE 3	SF-12-1-gesamt	SF-12-2-gesamt
10	10	26	28	26	26	38
10	10	26	28	30	20	27
3	3	25	26	26	20	33
12	12	29	30	30	20	30
4	4	30	29	30	26	34
7	7	28	30	30	16	23
9	9	26	25	25	32	29
10	10	27	29	30	23	32
5	5	24	29	29	25	28
11	11	30	30	30	34	34
5	5	29	30	30	23	36
23	23	28	28		23	33
12	12	23	28	29	35	29
5	5	24	24	23	29	30
27	27	24	27		28	37
17	17	18	23	17	20	26
11	11	26	27	30	30	42
4	4	25	27	24	17	32
7	7	26	24	30	26	30
26	26	22	20		21	20
3	3	29	30	30	20	34
4	4	30	30	30	20	41
8	8	29	27	30	30	39
8	8	27	30	30	25	34
12	12	27	29	30	23	34
1	1	27	30	29	26	42
4	4	28	27	29	23	39
7	7	29	30	24	43	40
15	15	28	28	30	25	31
7	7	19	24	21	20	33
	4	28	29		21	39
4	4	28	29	30	30	35
9	9	24	28	26	14	36
9	9	29	30	30	16	29
6	6	26	22	30	23	24
3	3	27	30	29	22	32
14	14	29	30	29	24	38
12	12	24	26	27	23	31
5	5	28	30	29	35	31
24	24	27	28	29	22	24
5	5	30	29	30	27	38
1	1	29	29	30	26	29
16	16	24	29	26	16	23
12	12	26	28	26	17	38
10	10	21	21	30	22	28
	32	28	25		15	12

	8	30	30		26	33
	4	21	25		22	34
28	28	23	21		35	24
10	10	22	26	20	23	31
30	30	23	25	20	21	17
14	14	26	28	28	25	28
17	17	28	27	27	27	19
25	25	25	20	14	20	20
	20	22			22	
	24	23			23	
17	17	24	29	28	31	24
21	21	29	27	28	24	22
5	5	26	30	30	32	35
15	15	27	29	30	18	23
11	11	29	30	30	19	21
30	30	28	30		22	8
14	14	30	28	30	23	31
2	2	26	30	30	34	32
5	5	24	27	29	31	26
10	10	23	29	29	30	18
13	13	28	29	20	33	25
10	10	28	27	30	26	20
23	23	30	30	29	23	20
15	15	26	22	27	23	20
	13	22	26		37	34
13	13	26	27	28	30	21
11	11	27	22	25	25	22
2	2	23	21	25	27	24
23	23	24	23	23	15	18
10	10	24	24	25	25	17
11	11	29	27	25	18	14
3	3	29	30	29	40	30
18	18	25	23	21	33	24
2	2	24	25	30	33	22
3	3	23	22	25	28	28
8	8	29	28	27	26	18
12	12	29	29	29	27	14
17	17	24	30	30	14	19
17	17	28	26	29	13	21
18	18	27	30	30	17	17
14	14	28	30		20	16
	26	26	28		30	22
17	17	24	21	19	38	32
23	23	22	22	10	19	16
38	38	21	27	27	16	17
32	32	25	27		28	28
18	18	22	26	21	30	29
26	26	23	21	24	33	29
25	25	23	25	24	26	29
20	20	28	30	30	22	30

27	27	27			18	19
10	10			30	23	15
31	31	23	14	15	24	21
21	21	26	28	27	20	25
12	12	19	30	26	22	25
18	18	28	26	29	24	25
31	31	28	30	27	17	16
37	37	27	26	23	27	14
18	18	29	29	28	26	22
15	15	26	19	12	21	32
17	17	26	28	28	22	20
13	13	30	30	30	33	31
8	8	25	24	26	27	32
25	25	23	23	22	22	22
15	15	21	25	21	34	16
19	19	28	25	25	38	17
	17	24	27		33	32
35	35	27	26	23	15	15
23	23	26	27	27	21	21
21	21	21	27	23	28	23
12	12	29	30		29	19
31	31	24	18	23	14	23
12	12	26	25	30	14	23
15	15	25	29	29	25	30
18	18	23	26	19	23	22
19	19	27	24	25	24	28
14	14	27	27	25	14	30
31	31	24	22	23	38	16
21	21	21	15	23	31	24
34	34	28	27	27	25	17
24	24	23	25	24	24	23
16	16	21	22	24	31	23
16	16	28	22	22	23	17
27	27	28	27	27	25	18
23	23	21	20	18	31	25
13	13	21	19	24	20	16
14	14	28	24	23	32	29
18	18	27	28	27	32	29
24	24	28	28	26	20	21
22	22	29	28	27	12	18
	19	30	30		33	24
	37	22	9		15	16
	28	26	25		29	18
	19	21	17		33	33
	23	23	22		26	20
	28	25	29		32	25
	19	24	25		30	28
	31	24	3		26	14
	18	24	22		19	30
	17	29			25	

13	27	17
20	21	15
27	28	22
12	26	35
14	21	28
27	30	16
22	27	17
20	24	27
17	28	30

nt

	15		
	23		
	23		
	13		
	27		
	13		
	16		
	16		
	5		
	21		
	10		
	20		
	24		
26	26	x	
18	18		29
13	13		28
27	27		24
23	23		27
	19		
	16		
	25		
24	24	x	
20	20	x	
19	19	x	
25	25		11
18	18	x	
18	18	x	
40	40		
14	14	x	
33	33	x	
10	10	x	
36	36		0
	14		

		16		
		10		
		21		
		11		
		10		
		17		
		23		
		8		
		17		
		11		
		31		
		24		
		14		
		16		
		22		
		16		
		19		
		13		
		18		
		19		
		21		
		12		
		13		
		21		
		41		
		23		
		15		
		29		
		23		
		19		
		20		
	27	27		25
		13		
		18		
		16		
		19		
		21		
		16		
		15		
		11		
		21		
		12		
		12		
		11		
		16		
x		13	x	
x		12	x	
x		25	x	
x		18	x	
x		20	x	

14
25
26
19
27
23
27
15

SF-12-3-gesamt	SF-12-1-phys	SF-12-2-phys	SF-12-3-phys	SF12-1-psych	SF12-2-psych	SF12-3-psych
34	9	16	15	17	22	19
30	8	9	13	12	18	17
42	8	14	17	12	19	25
23	9	10	9	11	20	14
29	9	11	10	16	23	19
24	7	8	8	9	15	16
33	14	12	13	18	17	20
24	10	13	10	13	19	14
34	7	9	8	18	19	26
38	9	10	12	25	24	26
44	9	15	16	14	21	28
23	9	14	10	14	19	13
30	16	13	12	19	16	18
33	15	11	11	14	19	22
24	13	16	11	15	21	13
21	9	9	7	11	17	14
27	10	17	9	20	25	18
33	10	12	8	7	20	25
30	7	9	10	19	21	20
20	7	7	6	14	13	14
37	7	13	12	13	21	25
40	9	17	17	11	24	23
40	13	16	15	17	23	25
41	13	18	17	12	16	24
33	10	14	14	13	20	19
46	15	18	18	11	24	28
34	9	16	11	14	24	23
28	18	15	11	25	25	17
29	7	9	8	18	22	21
26	9	14	11	11	19	15
28	11	15	14	10	24	14
43	14	12	17	16	23	26
28	6	13	9	8	23	19
22	6	10	9	10	19	13
21	7	7	7	16	17	14
33	6	9	10	16	23	23
28	12	17	12	12	21	16
30	9	11	12	14	20	18
34	12	7	8	23	24	26
19	8	10	8	14	14	11
34	11	14	12	16	24	22
41	13	12	15	13	17	26
23	7	7	8	9	16	15
27	6	14	10	11	24	17
25	7	12	8	15	16	17
	9	6		6	6	

	10	10		16	23	
	7	10		15	24	
19	14	10	8	21	14	11
32	12	11	12	11	20	20
14	10	7	6	11	10	8
27	7	7	9	18	21	18
17	7	6	6	20	13	11
20	6	6	6	14	14	14
	10			12		
	8			15		
23	8	9	8	23	15	15
28	11	12	11	13	10	17
34	13	11	12	19	24	22
26	13	10	7	15	13	19
31	8	7	13	11	14	18
16	8	16	6	14	10	10
34	7	10	13	16	21	21
45	11	13	18	23	19	27
33	10	11	12	21	15	21
30	11	7	8	19	11	22
41	14	15	18	19	10	23
34	13	10	13	13	10	21
22	11	8	10	12	12	12
26	6	7	9	17	13	17
24	15	12	11	22	22	13
31	10	7	13	20	14	18
26	10	7	9	15	15	17
34	7	9	10	20	15	24
19	6	6	8	9	12	11
37	10	6	15	15	11	22
25	6	6	7	12	8	18
43	18	10	16	22	20	27
23	13	8	9	20	16	14
28	12	9	10	21	13	18
26	7	10	9	21	18	17
26	9	8	12	17	10	14
22	8	6	8	19	8	14
22	6	6	8	8	13	14
19	6	7	8	7	14	11
20	7	9	9	10	8	11
34	8	6	11	12	10	23
	12	9		18	13	
27	16	12	11	22	20	16
20	11	7	8	8	9	12
14	6	6	6	10	11	8
15	13	13	8	15	15	7
25	12	11	7	18	18	18
23	13	11	8	20	18	15
25	10	11	12	16	18	13
20	8	9	7	14	21	13

21	8	10	11	10	9	10
23	9	6	8	14	9	15
18	10	8	8	14	13	10
24	10	11	11	10	14	13
23	7	8	10	15	17	13
28	11	9	10	13	16	18
17	7	6	6	10	10	11
14	11	6	6	16	8	8
22	8	7	7	18	15	15
33	13	12	12	19	20	21
27	7	6	9	15	14	18
30	12	12	12	21	19	18
37	12	10	14	15	22	23
19	7	6	9	15	16	10
26	14	8	11	20	8	15
21	18	6	7	20	11	14
30	13	12	13	20	20	17
12	6	6	6	9	9	6
20	11	10	7	10	11	13
25	10	12	9	18	11	16
27	9	6	8	20	13	19
19	6	7	8	8	16	11
32	6	13	12	8	20	20
25	12	8	8	13	22	17
23	11	9	10	12	13	13
24	11	12	11	13	16	13
28	6	10	9	8	20	19
20	15	8	7	23	8	13
27	11	9	10	20	15	17
14	9	6	6	16	11	8
15	7	7	6	17	16	9
17	11	9	7	20	14	10
26	8	8	11	15	9	15
16	8	6	6	17	12	10
29	14	13	14	17	12	15
25	8	6	8	12	10	17
32	13	8	11	19	21	21
26	11	11	11	21	18	15
16	6	6	6	14	15	10
16	6	6	6	6	12	10
	13	10		20	14	
	6	9		9	7	
	10	7		19	11	
	14	16		19	17	
	9	6		17	14	
	8	7		24	18	
	8	8		22	20	
	13	6		13	8	
	10	12		9	18	
	10			15		

13	14
6	9
10	12
12	23
12	16
7	9
8	9
9	18
12	18

8 9

Pflegestufe1	Pflegestufe2	Zahl Sturz1	Zahl Sturz2	Zahl Sturz3	Pearlin1	Pearlin2
1	1	2	0	3	9	13
1	1	3	2	1	11	14
0	0	0	0	0	10	12
0	0	0	0	0	13	18
3	3	4	0	0	10	13
2	2	1	0	1	10	10
0	0	0	0	1	12	14
0	0	1	0	2	15	12
1	1	2	1		20	17
1	1	6	6	5	20	19
0	0	2	0	1	18	19
		1	0	0	13	14
0	0	1	0	1	14	16
1	1	1	0	0	11	10
0	0	0	0	4	15	19
1	1	0	0	0	8	10
0	0	0	0	0	14	17
1	1	1	1	1	13	15
0	0	0	0	0	13	14
1	1	2	3	4	10	10
0	0	0	2	0	13	18
0	0	6	0	0	13	19
		2	0	0	14	17
0	0	1	1	0	10	12
0	1	3	0	1	13	17
0	0	1	1	0	14	20
1	1	1	0	1	11	15
0	0	1	0	3	20	20
1	1	1	1	0	10	13
		1	0	2	15	10
0	0	6	3	4	10	19
0	0	0	1	1	20	13
0	1	5	2	1	12	17
0	0	2	1	3	8	11
1	1	1	0	0	10	8
1	1	0	0		15	15
0	0	0	0	0	10	10
0	0	3	1	8	9	10
1	1	1	1	0	15	19
0	0	2	1	0	11	10
0	1	1	1	2	14	18
0	0	2	0	0	10	10
1	1	21	15	6	13	17
1	1	1	0	0	15	15
0	0	2	0	1	10	10
		0	0		11	11

1	1	3	0		15	16
0	1	1	0		10	14
0	0	1	0	0	14	14
2	2	1	0	0	11	13
1	2	2	0	1	10	12
1	1	0	0	3	13	19
1	1	2	0	1	10	15
0	0	3	1	0	15	16
		0			10	
		2			15	
0	0	4	1	2	14	14
0	0	0	0	0	10	10
1	1	0	0	0	13	15
0	0	2	2	3	11	14
0	0	4	5	3	7	13
0	1	1	0	3	13	9
1	1	0	0	0	13	16
0	0	0	1	0	18	14
1	2	1	0	0	11	12
1	1	2	1	3	15	12
0	0	0	1	1	11	12
0	0	1	0		14	13
1	1	1	0	0	17	6
0	0	3	0	0	14	12
0	0	0	0	0	14	16
0	1	0	0	1	13	11
2	2	3	0	3	13	10
0	1	1	4	8	17	10
0	0	0	1	1	7	13
0	1	1	1	0	14	7
1	1	0	0	0	13	5
0	0	0	0	0	20	18
0	0	4	5	8	18	13
0	1	2	15	3	15	12
1	1	6	2	1	13	14
0	0	3	0	0	19	11
0	0	12	5	2	15	5
1	1	0	0	0	10	13
0	0	0	0	0	8	12
0	1	1	0	2	10	11
0	0	3	0	1	10	6
0	1	3	0		13	10
0	0	0	0	0	18	15
0	1	1	1	1	14	11
0	1	3	3	3	11	10
1	1	1	1	1	12	13
0	0	1	0	1	10	10
2	2	0	0	3	13	14
0	2	0	0	1	10	13
0	0	0	0	1	12	14

0	0	2	1	0	8	8
0	1	1	0	1	12	14
0	2	1	7	3	10	13
0	1	1	0	0	12	14
0	1	1	0	1	15	15
0	2	0	0	0	14	14
0	1	1	0	0	10	5
0	1	0	0	0	14	10
1	1	5	0	1	13	11
0	1	2	0	1	15	15
1	1	0	0	0	15	16
0	0	0	0	0	16	15
0	1	4	2	1	14	14
1	2	25	20	22 (?)	11	8
2	2	0	0	2	15	5
		1	0	0	14	8
0	2	2	0	0	13	12
0	0	0	0		13	7
0	1	1	0	0	14	13
0	0	1	1	3	13	14
0	0	2	3		17	10
2	2	1	1	0	6	16
0	1	6	4	4	14	10
0	0	0	4	0	6	18
0	1	2	2	0	14	10
1	1	1	2	1	12	15
0	1	0	0	2	16	17
0	1	1	2	2	17	8
0	2	0	0	0	13	7
1	1	1	0	0	15	12
1	1	5	0	0	14	15
0	1	2	3	3	19	15
1	3	2	3	0	12	7
1	1	0	0	0	14	11
0	2	4	1	0	15	10
0	0	2	0	0	6	8
0	1	3	0	0	14	15
0	0	4	0	6	15	13
0	1	1	2	0	13	12
0	1	6	0	1	15	15
0	1	15	8		15	18
1	3	1	12		11	5
1	1	0	1		13	10
2	3	0	0		17	18
1	2	2	2		16	14
0	1	10	0		19	14
		3	2		14	15
0	3	1	0		10	5
		1	0		10	14
0		5			15	

0	11
3	6
1	8
1	19
12	9
0	5
2	7
17	8
0	13

0

Pearlin3	SoS-gesamt1	SoS-gesamt2	SoS-gesamt3	IADL-1	IADL2	IADL3
11	17	23	23	5	6	3
15	17	16	20	3	1	1
15	13	18	18	2	4	5
15	20	22	17	5	5	5
15	14	17	16	2	2	2
12	18	21	19	5	6	5
13	17	19	18	7	7	8
14	15	18	23	7	8	8
18	14	17	16	2	4	3
19	23	23	23	6	8	7
20	23	22	22	8	8	8
5	18	20	17	8	7	0
12	18	22	21	8	8	7
11	18	21	19	6	1	1
8	19	20	17	6	3	1
6	16	19	16	1	1	1
15	22	22	22	8	8	8
17	16	18	18	5	1	1
13	16	17	18	7	4	4
7	17	19	17	1	1	1
15	20	22	22	5	6	7
18	23	25	25	7	8	8
15	18	19	20	4	6	6
19	15	22	22	7	6	6
13	11	16	17	7	7	6
20	19	21	22	8	8	8
13	17	19	21	4	3	4
17	18	18	14	6	8	8
15	17	18	18	0	2	3
10	15	16	16	2	6	1
10	13	21	20	5	8	8
16	20	21	22	6	7	7
12	18	20	22	6	8	6
13	18	21	18	5	7	1
13	21	21	22	1	2	1
19	19	21	21	6	7	7
12	18	21	21	2	3	3
12	14	17	17	6	6	7
20	19	21	20	2	6	6
10	18	22	23	4	6	3
19	18	21	20	4	5	6
15	19	21	22	5	8	8
13	11	15	16	6	7	7
15	16	22	22	8	8	8
10	18	19	22	1	2	2
	17	18		4	3	

	15	19		5	6	
	16	16		1	1	
	16	17	16	5	5	6
15	16	17	16	1	2	1
14	15	12	11	0	0	0
15	19	19	19	5	6	7
11	16	14	14	5	4	4
16	15	16	13	2	3	0
	24			2		
	12			3		
11	19	16	16	7	6	3
12	16	15	17	2	3	6
15	20	21	21	6	7	7
12	19	19	19	6	6	6
15	13	21	20	8	8	8
12	19	18	16	6	5	5
15	21	22	23	6	6	8
20	20	20	21	7	5	8
15	16	16	19	8	1	1
15	19	16	17	6	1	5
7	12	13	15	7	8	8
13	24	20	23	8	7	8
10	18	17	17	5	4	5
11	17	17	20	4	5	4
10	22	20	22	8	2	3
11	19	16	17	3	5	3
12	16	14	18	2	1	1
18	22	20	23	2	1	3
9	17	13	15	7	7	7
17	18	11	14	5	2	7
8	17	12	18	1	0	0
19	23	20	22	8	4	5
15	22	18	18	7	2	6
15	19	14	16	6	2	4
16	19	18	21	8	2	5
13	17	14	18	7	7	7
10	16	15	21	7	2	2
12	10	18	15	1	1	2
8	16	18	18	5	3	5
13	12	9	12	4	2	6
9	20	16	18	7	6	6
	18	16		2	2	
15	21	19	18	6	6	6
11	18	17	17	5	2	0
5	21	18	18	8	3	3
5	19	15	13	5	1	0
10	17	17	19	2	3	3
9	18	14	16	1	1	1
12	22	19	19	6	2	2
15	17	19	17	7	7	6

11	15	15	15	8	8	7
15	17	16	14	6	5	8
12	18	16	13	1	0	0
12	20	14	14	8	0	0
14	20	15	18	8	8	5
15	19	17	18	6	1	1
7	18	16	11	7	2	1
5	17	17	17	2	1	0
10	17	17	17	3	2	2
15	20	17	15	5	1	0
15	19	17	16	7	1	1
14	19	19	19	8	8	8
15	20	20	20	5	1	1
8	17	17	17	3	1	0
10	19	19	18	7	2	1
10	22	18	17	7	7	6
15	23	19	22	6	1	
5	12	13	11	6	5	2
10	18	15	15	6	5	2
10	22	19	19	7	6	1
12	16	14	18	5	2	1
13	15	20		1	3	1
13	14	16	19	8	1	2
17	17	18	18	4	6	5
13	13	12	15	2	1	1
13	15	13	13	2	1	0
16	21	20	19	2	1	1
9	18	17	15	7	1	1
14	21	14	16	8	0	0
6	21	20	18	3	2	1
11	20	22	19	5	3	3
11	20	16	15	2	1	1
11	19	18	18	4	0	0
14	17	17	17	4	4	2
13	20	16	17	6	5	3
12	14	17	18	4	0	1
15	17	20	20	6	2	2
14	21	17	14	8	7	6
9	17	17	16	4	3	2
12	11	18	16	6	1	1
	19	17		3	3	
	17	16		8	0	
	20	15		5	0	
	17	12		2	1	
	21	18		2	1	
	21	19		7	2	
	20	21		5	2	
	18	16		4	0	
	13	15		3	3	
	19			6		

14	5
18	1
13	1
21	2
18	4
19	5
15	4
13	1
20	7

12 7

CIRS 1	CIRS2	TimedUGTest 1	TimedUGTest 2	TimedUGTest 3	5chairrise	5chairrise2
17	11	18	12	14	14,2	10
12	12	15	13	10	17	15
12	6	20	11	15	16	11
19	17	15	11	19	21	15
18	15					
11	9	16	18	30	23	25
15	15	11	9	13	12	9
11	10	17	9	10	17	19
18	16	20	39	59	16	105
17	16	34	12	10	18	13
10	12	14	12	14	13	8
18	17	25	18		19	28
14	13	19	14	15	13	15
18	18					
15	10	17	11	54	13	11
20	17	27	37		28	25
12	10	15	4	6	8	9
16	10	23	19	23	17	26
22	17	19	35	15	21	59
17	17	26	29		37	58
22	19	26	14	16	17	14
11	9	21	6	11	20	10
16	14	16	16	18	10	14
14	8	26	10	11	10	10
21	17	11	13	17	22	20
9	7	10	10	11	12	12
10	8	15	12	22	20	18
17	14	18	16		12	11
13	12	31	32	34	30	19
19	21	25	14	19	18	22
16	14	14	10	13	23	20
11	9	8	10	11	15	13
22	15	28	28	13	50	28
16	12	25	26			30
17	15		21	23		22
18	11	32	14	26	22	16
10	9	11	9	10	12	10
13	11	20	8	7	11	13
12	13		16	18		13
11	10		17	17	25	33
16	13	14	10	16	17	13
14	13	23	15	13	14	14
16	15	16	14	19	18	20
14	12	22	17	7	21	28
17	15	14	23	22	21	23
24	24	14	14		27	30

22	21	18	15		19	20
21	18					
9	9	25	23		13	17
25	21					
13	15					
16	17	20	23	21	30	39
18	19	22	26	35	18	22
17	18	25	25	26	30	25
19						
16		45			29	
16	17	13	16	29	20	21
9	12	9	14	15	19	24
16	19	14	22	14	15	19
16	18	17	19	20	12	20
15	18	16	13	9	13	23
20	20		54			45
11	10	18	16	17	22	25
16	16	28	17	18	24	23
16	18					
14	17				31	
10	13	11	12	14	14	14
16	15	11	11	10	12	16
13	18	53	32	22		23
13	16	17	27	26	19	26
18	21	17	21	16	21	21
13	19	13	20	21	14	27
15	17	30	65	75	125	89
25	24	16		29	50	
16	18	11	17	19	25	17
17	18	11	11	9	19	15
17	21	15	23	25	29	43
19	19	11	24	17	12	17
21	27	14	12	18	13	13
18	21	28	43	13	19	32
15	19	24	19	10	17	15
17	17	29	34	29	23	34
15	17	15			11	
14	13	15	25		21	33
17	14	17	19	27	30	17
21	27	13	27	26	16	58
16	16	22	25	29	31	36
15	16	12	17		25	25
9	9	12	11	16	10	11
14	18	14	35	35	14	22
15	15	15	13	16	15	15
14	16	25	16		17	20
18	16	18	14	21	13	9
21	25	27	28		21	33
23	21					
13	14	26	19	22	29	12

15	15	20	21	22	14	16
10	11			23		
12	15	16			16	
26	26	28	23	22	20	19
13	12	23	15	22	16	19
20	22					
13	11	17	24	44	26	25
12	16	30	59	> 120 s	32	65
16	17	20	29	35	30	26
13	13	21	14	19	32	19
23	24	25	40	41	16	31
10	10	18	9	10	8	10
15	14	32	33	30	16	24
19	20					
20	20	24	21	29		23
14	13	8	11	13	8	10
23	22					
16	17	17	27	38	30	85
13	14	9	14	16	22	30
20	24	16	17	24	21	22
15	15	30	35			67
19	20					
14	13		24	24	60	22
14	12	8	9	10	7	14
16	17	29	27	30	15	20
10	12	25	26	27	35	36
17	16	12	15	18	33	
20	24	19			15	
13	16	18		20	28	
17	18	18	24	42	14	24
19	18	16	19	24	30	28
18	15	54	24	27	26	37
13	21					
17	22	18	22	28	16	28
15	17	22	12	13	17	22
16	17	15	28	32	23	35
20	21	26	32	29	31	34
19	20	18	22	32	22	28
17	17	17	15	18	19	27
17	18	20	36	42	27	43
22	25	12	18		17	25
13	18	18			17	
19	20	54			30	
20	20					
20	21					
19	20	22			16	
19	20					
14	18	48				
16	21	14	26		15	19
16		16			19	

14	14	12
23		
16		
16	15	16
25	32	41
20	19	17
17	18	51
25		
14	18	25

5chairrise3	HandkraftMax1	Handkraftmax2	HandkraftMax3	Waist-Hip-ratio1	Waist-Hip-ratio2	Waist-Hip-ratio3
13	50	80	58	1,01	1,01	1,01
16	50	50	80	0,91	1,04	1,08
11	75	90	90	0,9	0,97	0,92
18	55	60	54	0,86	0,9	0,95
	70	55	48	0,88	0,87	0,86
61	40	50	48	0,9	0,93	0,8
17	60	60	55	1	0,98	0,97
16	40	30	50	0,91	0,91	0,88
	60	55	44	0,78	0,78	0,81
10	48	50	52	0,93	0,91	0,99
11	45	50	54	0,95	0,83	0,89
	40	35	0	0,89	0,83	0,83
18	32	40	38	0,89	0,97	0,97
	35	20	16	0,92	0,87	0,95
	50	60	34	0,6	0,87	0,9
	35	25	18	0,9	0,93	0,95
15	14	35	16	0,97	0,89	0,97
23	40	75	60	0,92	0,86	0,85
24	65	52	70	0,8	0,96	0,92
	2	6	2	0,97	0,92	0,98
13	58	80	68	0,89	0,92	0,89
12	60	80	52	0,87	0,73	0,85
24	60	74	50	0,96	0,98	1,03
11	70	80	80	0,96	0,97	0,94
36	75	68	36	0,96	0,97	1,03
12	43	55	54	0,86	0,8	0,93
16	48	50	50	0,82	0,82	0,8
	75	58	48	0,76	0,71	0,83
16	42	90	48	0,98	0,87	0,94
27	60	52	52	1,01	1,02	0,97
21	40	54	46	0,88	0,9	0,88
13	60	64	62	0,95	1	1
25	30	32	38	0,93	0,96	
	38	42	40	0,9	0,92	
25	38	54	50	0,89	0,83	0,93
19	55	48	62	0,88	0,88	0,87
11	32	34	32	0,82	0,77	0,78
13	50	44	65	0,9	0,83	0,82
13	80	70	64	0,81	0,86	0,89
36	40	40	18	0,88	0,89	0,82
16	72	74	68	0,95	0,93	0,91
16	36	38	48	0,83	0,84	0,92
20	26	42	55	0,89	0,91	0,91
25	60	48	46	0,92	0,88	0,84
25	32	32	48	1,01	0,91	1,1
	50	45	0	0,89	0,94	

	40	70	0	1,02	0,97	
	50	20	0	1,05		
	40	45	0	0,89	0,9	
	10	35	4	0,78	0,86	0,94
	30	28	20	0,85	0,89	0,88
36	16	16	20	0,9	0,98	0,98
30	60	60	60	1		0,97
26	60	60	50	0,85	0,87	0,88
	70	0	0	0,92		
	16	0	0	0,85		
	50	50	45	0,83	0,88	0,9
19	50	45	50	0,82	0,8	0,78
17	35	60	60	0,88	0,93	0,88
18	65	75	72	0,87	0,87	0,93
14	90	80	100	0,82	0,87	0,94
	20	32	54	0,85	0,91	0,95
30	40	44	54	0,89	0,99	0,99
16	75	85	80	0,9	0,92	0,92
	45	38	16	0,87	0,88	0,88
	50	56	60		0,93	1,04
15	90	80	80	1,03	1	1,04
17	30	46	42	0,95	0,85	0,86
21	55	45	46	0,89	0,9	0,9
34	45	40	30	0,88	0,9	
22	60	72	68	0,86	0,88	0,83
48	50	55	70	0,89	1,08	0,9
51	20	40	18	0,91	0,92	
23	10	5	0	0,85	0,86	0,89
23	45	68	56	1,13	0,9	0,86
12	60	40	40	0,79	0,45	0,45?
26	40	30	46	0,96	0,98	1,02
16	90	90	92	0,93	1,05	0,99
17	48	42	50	0,86	0,84	0,86
16	88	60	62	0,96	0,95	1,04
15	54	50	54	0,87	0,84	0,85
33	54	35	40	0,89	0,9	0,88
	50	38	40	0,9	0,94	0,93
	20	20	14	1,02	1,02	
33	1	28	28	0,84	0,87	0,85
54	30	48	50	1,03	1,02	1,01
50	44	28	38	0,87	0,93	0,93
	40	20	0	0,86	0,61	
16	60	45	40	0,92	0,6	0,59
	45	45	28	0,98	0,88	0,94
15	50	60	45	0,89	0,86	0,86
	35	50	0	0,8	0,81	0,82
18	50	70	62	0,98	0,96	1,03
	40	45	48	0,98	0,89	0,95
	25	12	1	0,89	0,94	0,88
19	33	25	20	95,3	0,88	0,89

20	65	60	55	0,99			0,99
	65	60	50	0,77	0,78		0,81
	50	10	5	0,89	0,9		0,89
20	60	50	60	0,85	0,76		0,76
32	40	30	20	0,88	0,88		0,87
	10	34	28	0,84	0,88		0,93
30	45	30	15	1,01	0,86		0,86
90	50	35	10	0,94	1		0,99
34	50	50	50	0,91	0,84		0,82
25	40	45	40	0,93	0,9		0,89
38	25	4	2	1,07	0,94		0,93
12	70	60	50	0,89	0,88		0,88
26	40	40	40	0,92	0,91		0,92
	58	60	20	1,07	0,98		1,03
21	50	40	65	0,83	0,92		0,85
12	65	40	45	0,81	0,8		0,8
	40	40	40	1,02	0,91		
115	65	30	10	1,09	1,06		1,01
34	63	50	50	1,01			0,99
28	75	75	60	0,93	0,91		0,91
	42	25	20	0,87	0,88		0,89
	20	5	20	0,85	0,85		0,85
18	80	60	56	0,89	0,88		0,92
15	35	22	30	0,96	0,84		0,83
28	60	55	40	0,92	0,92	0.93	
38	27	25	20	0,84	0,85		0,86
	20	20	15	0,84	0,84		0,83
	65	45	30	0,81	0,92		0,91
30	55	30	60	0,83	0,82		0,82
60	30	20	10	0,95	0,96		0,96
35	2	18	15	0,89	0,84		0,85
45	48	62	50	0,92	0,93		0,93
	28	0	0	0,9			0,93
35	40	30	30	0,91	0,94		0,91
27	30	32	30	1,07	0,97		0,96
40	18	10	20	1	1,03		1,02
38	64	70	60	0,91	0,99		0,98
44	92	70	70	0,94	0,94		0,93
42	86	32	30	0,96	0,99		0,96
46	45	35	30				
	48	52	0	0,92	0,95		
	70	2	0	0,79	0,78		
	29	20	0	0,82	0,89		
	50	60	0	1,02	1,1		
	35	10	0	0,92	0,84		
	50	33	0	0,86	0,87		
	10	30	0	1	0,96		
	8	0	0	0,83	0,84		
	70	60	0	1,1	0,99		
	35		0	0,88			

70	0	0,86
10	0	1,06
40	0	0,93
70	0	0,97
40	0	0,94
40	0	0,81
48	0	0,89
12	0	0,93
30	0	0,96
	0	
	0	
	0	
	0	
	0	
	0	

12	0,97

Barthel1	Barthel1LOCF	Barthel2	Barthel2LOCF	Barthel3	Barthel3LOCF	Karnofsky1
85	85	95	95	85	85	60
75	75	75	75	95	95	55
65	65	95	95	95	95	55
70	70	90	90	80	80	80
30	30	30	30	40	40	40
90	90	55	55	85	85	60
85	85	100	100	100	100	60
90	90	90	90	95	95	80
65	65	75	75	80	80	50
60	60	90	90	95	95	50
90	90	100	100	100	100	60
75	75	95	95	5	5	60
95	95	90	90	95	95	65
40	40	25	25	30	30	35
90	90	100	100	85	85	40
85	85	75	75	35	35	40
90	90	95	95	95	95	70
65	65	80	80	60	60	40
80	80	85	85	95	95	50
75	75	85	85	35	35	35
95	95	95	95	95	95	50
95	95	100	100	95	95	50
80	80	90	90	90	90	35
100	100	90	90	90	90	60
65	65	100	100	90	90	40
85	85	95	95	100	100	50
80	80	95	95	80	80	50
85	85	100	100		100	55
65	65	70	70	80	80	50
65	65	65	65	80	80	45
80	80	95	95	95	95	55
95	95	100	100	95	95	55
85	85	95	95	85	85	50
75	75	65	65	40	40	50
80	80	70	70	80	80	40
90	90	85	85	85	85	55
70	70	90	90	90	90	40
70	70	75	75	90	90	50
70	70	70	70	65	65	50
90	90	80	80	70	70	55
80	80	80	80	75	75	55
85	85	95	95	90	90	55
85	85	90	90	90	90	51
85	85	90	90	85	85	50
85	85	90	90	80	80	60
80	80	75	75		75	40

85	85	75	75		75	50
75	75	55	55		55	40
85	85	85	85	70	70	75
35	35	30	30	35	35	35
60	60	20	20	15	15	40
85	85	85	85	85	85	55
85	85	70	70	70	70	60
75	75	80	80	75	75	55
20	20		20		20	35
85	85		85		85	55
90	90	80	80	85	85	60
95	95	85	85	90	90	70
85	85	90	90	90	90	60
95	95	65	65	70	70	70
100	100	90	90	95	95	80
55	55	60	60	55	55	50
95	95	95	95	100	100	80
70	70	95	95	95	95	80
55	55	40	40	40	40	40
65	65	55	55	70	70	40
100	100	100	100	95	95	70
90	90	95	95	100	100	55
65	65	60	60	75	75	50
90	90	75	75	90	90	60
85	85	90	90	100	100	60
75	75	85	85	90	90	55
75	75	45	45	65	65	45
70	70	55	55	75	75	55
95	95	90	90	90	90	60
90	90	90	90	95	95	55
85	85	45	45	75	75	50
95	95	95	95	100	100	65
90	90	75	75	55	55	60
70	70	60	60	75	75	60
85	85	80	80	75	75	75
75	75	75	75	85	85	50
65	65	20	20	40	40	40
75	75	70	70	30	30	45
85	85	95	95	85	85	60
100	100	80	80	90	90	60
85	85	80	80	100	100	50
85	85	80	80		80	60
95	95	90	90	85	85	80
85	85	80	80	35	35	75
60	60	70	70	60	60	55
75	75	85	85	0	0	60
90	90	85	85	85	85	45
55	55	30	30	30	30	40
5	5	10	10	20	20	35
85	85	85	85	85	85	70

85	85	90	90	85	85	55
25	25	65	65	75	75	41
55	55	30	30	35	35	60
80	80	80	80	80	80	60
75	75	8	8	100	100	50
65	65	50	50	5	5	50
70	70	60	60	55	55	50
85	85	30	30	30	30	40
90	90	75	75	50	50	60
65	65	75	75	60	60	40
85	85	60	60	60	60	70
95	95	100	100	100	100	80
65	65	60	60	70	70	60
50	50	30	30	35	35	50
60	60	55	55	55	55	60
100	100	95	95	90	90	70
30	30	35	35		35	45
80	80	60	60	55	55	55
95	95	85	85	75	75	60
90	90	85	85	55	55	55
80	80	75	75	45	45	55
35	35	25	25	25	25	30
80	80	65	65	70	70	70
75	75	90	90	90	90	60
85	85	70	70	60	60	50
65	65	65	65	65	65	50
85	85	80	80	65	65	50
90	90	35	35	30	30	55
85	85	45	45	60	60	60
70	70	55	55	35	35	50
85	85	80	80	65	65	55
20	20	85	85	65	65	20
40	40	10	10	15	15	50
60	60	65	65	50	50	40
90	90	75	75	70	70	60
65	65	40	40	55	55	40
55	55	65	65	65	65	40
85	85	80	80	75	75	70
90	90	70	70	60	60	60
85	85	70	70	60	60	60
85	85	65	65		65	30
85	85	5	5		5	55
55	55	25	25		25	50
5	5	15	15		15	40
35	35	15	15		15	30
70	70	40	40		40	55
70	70	40	40		40	60
60	60	0	0		0	50
75	75	90	90		90	60
75	75		75		75	50

95	95		95		95	60
60	60		60		60	35
25	25		25		25	40
80	80		80		80	50
70	70		70		70	50
80	80		80		80	40
80	80		80		80	55
15	15		15		15	35
85	85		85		85	60

Barthel1	Barthel1	Barthel2	Barthel2		
25	25	30	30		30
75	75	75	75		75
85	85	60	60		60
80	80	90	90		90
100	100	90	90		90
80	80	70	70		70
90	90	90	90		90
25	25	30	30		30
95	95	100	100		100
90	90	85	85		85
50	50	40	40		40
70	70	70	70		70
30	30	20	20		20
60	60	20	20	20	20
70	70	55	55	55	55
90	90	100	100	100	100
75	75	80	80	80	80
60	60	50	50	50	50
60	60	60	60		60
85	85	80	80		80
65	65	60	60		60
85	85	70	70	70	70
90	90	90	90	90	90
80	80	55	55	55	55
45	45	30	30	30	30
45	45	35	35	35	35
95	95	95	95	95	95
45	45	10	10	10	10
85	85	80	80	80	80
85	85	50	50	50	50
70	70	70	70	70	70
20	20	10	10	0	0
85	85	90	90		90

85	85	95	95		95
85	85		85		85
60	60	60	60		60
85	85	90	90		90
70	70	85	85		85
70	70	70	70		70
60	60		60		60
85	85		85		85
95	95	40	40		40
70	70	70	70		70
90	90	85	85		85
85	85	35	35		35
85	85	80	80		80
90	90	75	75		75
40	40	40	40		40
80	80	80	80		80
85	85	85	85		85
65	65	65	65		65
85	85	85	85		85
50	50	60	60		60
90	90	85	85		85
85	85	85	85		85
55	55	60	60		60
95	95	60	60		60
85	85	0	0		0
75	75	70	70		70
75	75	75	75		75
10	10	15	15		15
75	75	50	50		50
40	40	30	30		30
90	90	85	85		85
65	65	100	100	100	100
80	80	75	75		75
90	90	80	80		80
90	90	90	90		90
65	65	55	55		55
90	90	90	90		90
90	90	90	90		90
50	50	70	70		70
85	85	75	75		75
55	55	50	50		50
75	75	75	75		75
45	45		45		45
95	95	95	95		95
80	80	75	75		75
90	90		90		90
90	90		90		90
15	15		15		15
85	85		85		85
85	85		85		85

65	65		65	65
85	85		85	85
80	80	60	60	60
70	70	65	65	65
85	85	0	0	0
95	95	95	95	95
85	85	75	75	75
90	90	85	85	85

Karnofsky2	Karnofsky2L OCF	Karnofsky3	Karnofsky3L OCF
70	70	60	60
45	45		45
80	80	80	80
80	80	75	75
40	40		40
70	70		70
60	60		60
80	80		80
50	50		50
75	75		75
70	70		70
60	60	30	30
80	80		80
40	40		40
55	55	50	50
40	40		40
80	80	80	80
60	60		60
65	65		65
50	50		50
75	75	80	80
70	70		70
50	50		50
75	75	80	80
75	75	80	80
65	65		65
50	50	60	60
65	65		65
60	60	60	60
50	50	55	55
75	75	80	80
65	65	80	80
75	75	75	75
60	60	40	40
40	40	60	60
75	75	75	75
55	55	55	55
75	75	80	80
55	55	55	55
60	60	50	50
65	65	75	75
70	70	70	70
80	80	75	75
80	80	80	80
60	60	55	55
35	35		35

55	55		55
40	40		40
60	60		60
40	40		40
40	40	35	35
60	60	55	55
60	60	60	60
45	45	35	35
	35		35
	55		55
60	60		60
55	55		55
55	55		55
60	60		60
65	65		65
40	40		40
60	60		60
75	75		75
40	40		40
35	35		35
60	60		60
60	60	75	75
45	45	60	60
60	60	55	55
55	55	50	50
50	50		50
40	40	55	55
35	35	55	55
55	55	75	75
40	40	60	60
40	40	45	45
60	60	75	75
55	55		55
40	40	70	70
55	55	65	65
50	50	60	60
35	35	40	40
45	45	40	40
45	45	60	60
55	55	75	75
50	50	60	60
55	55		55
80	80	70	70
40	40	40	40
55	55		55
50	50	10	10
40	40		40
40	40	40	40
50	50	40	40
60	60	60	60

60	60	65	65
50	50	60	60
40	40	35	35
35	35	40	40
50	50	60	60
40	40	50	50
45	45	40	40
35	35	35	35
40	40	40	40
40	40	40	40
45	45	40	40
80	80	80	80
55	55	55	55
40	40	35	35
40	40		40
60	60	60	60
40	40	55	55
45	45	40	40
50	50	55	55
55	55	45	45
40	40	40	40
30	30	40	40
40	40	60	60
60	60	60	60
40	40	45	45
40	40	40	40
55	55	50	50
35	35	35	35
40	40	55	55
40	40	35	35
50	50	55	55
55	55	50	50
15	15	35	35
45	45	40	40
60	60	50	50
35	35	50	50
40	40	45	45
55	55	45	45
40	40	35	35
40	40	35	35
55	55		55
35	35		35
35	35		35
40	40		40
20	20		20
25	25		25
55	55		55
10	10		10
50	50		50
	50		50

60	60
35	35
40	40
50	50
50	50
40	40
55	55
35	35
60	60

Stürze1	MEL-life events1
0	82
0	98
1	221
1	121
0	142
0	160
1	127
0	246
0	179
0	53
0	53
0	53
0	152
0	53
1	77
1	53
1	53
0	92
1	160
0	159
1	53
0	197
0	214
0	153
0	97
0	53
0	77
0	71
0	53
0	53
0	82
1	71
0	277
0	134
0	53
1	96
1	135
0	214
0	116
0	53
0	134
0	72
0	172
0	153
0	92
1	114

0	139
0	72
0	90
0	90
0	53
1	203
0	196
0	114
1	160
0	71
0	73
0	152
1	116
0	196
0	71
0	77
1	134
1	116
0	53
0	97
1	153
0	116
0	289
0	133
1	116
1	90
0	197
0	77
0	106
0	163
1	219
1	77
0	90
0	97
0	97
0	97
0	71
1	73
1	158
0	253
0	131
0	108
1	155
0	136
0	53
0	53
0	281
1	53
1	90
1	53

0	171
0	127
0	53
0	53
0	53
1	53
0	53
1	53
0	53
0	129
1	53
1	115
0	112
0	153
1	53
0	53
0	72
1	242
0	53
0	53
0	140
0	127
0	97
1	53
0	115
0	123
1	91
0	77
1	53
0	108
0	139
0	116
0	53
1	53
0	53
0	90
0	82
0	116
0	234
0	53
0	245
0	53
1	97
0	130
0	90
0	53
0	72
0	216
0	77
0	71

1	169
0	108
0	228
0	116
0	153
1	245
0	194
1	174
1	53

Anzahl Tab Psych1	Antidepressiva?1	Seit wann AD?1	Psychopharmaka		Frailty-Index1
1,5	1	2	1		12+2=14
18	1	2	1		11+2=13
1,5	1	1	1		11+3=14
2	1	2	1		9+2=11
3	1	1	1		11+4=15
1	1	1	1		12+3=15
1	1	1	1		10+2=12
1	1	1	1		8+1=9
1	1	1	1		8+3=11
2	1	2	1		8+3=11
0,5	1	2	1		8+2=10
2	1	1	1		9+3=12
0	0	0	0		6+3=9
4	1	1	1		15+4=19
1	1	2	1		9+3=12
2	1	2	1		12+3=15
1	1	1	1		7+2=9
3	1	1	1		11+3=14
0	0	0	0		10+3=13
2	1	2	1		13+3=16
1,5	0	2	2		9+2=11
0	0	0	0		8+2=10
1,5	1	1	1		11+3=14
1,5	1	2	1		8+2=10
3	1	2	1		11+3=14
1	1	1	1		8+2=10
2	1	2	1		7+2=9
1	1	1	1		7+2=9
0	0	0	0		12+3=15
1	1	1	1		11+3=14
3	1	2	1		12+2=14
0	0	0	0		8+2=10
1	1	2	1		10+2=12
1	1	1	1		9+2=11
1	1	1	1		9+2=11
2	1	2	1		5+2=7
4	1	2	1		10+2=12
2	1	2	1		14+2=16
0	0	0	0		8+3=11
2	1	1	1		10+2=12
1	1	1	1		6+2=8
5	1	2	1		8+2=10
2	1	2	1		12+2=14
2	1	2	1		10+2=12
1	1	1	1		9+2=11
2	1	2	1		8+3=11

0	0	0	0	10+2=12
0	0	0	0	13+3=16
2	1	2	1	10+2=12
4	1	2	1	13+4=17
1	1	1	1	12+4=16
1	1	1	1	8+2=10
0	0	0	0	9+2=11
0	0	0	0	13+2=15
2	1	1	1	12+4=16
1	1	1	1	13+2=15
0	0	0	0	8+2=10
3	1	2	1	8+1=9
1	1	2	1	6+2=8
0	0	0	1	7+2=9
1	1	2	1	5+1=6
1	1	1	1	10+3=13
0	0	0	0	8+1=9
0	0	0	0	3+1=4
2	1	1	1	12+4=16
1,5	1	1	1	9+3=12
1	1	1	1	5+2=7
0,5	0	2	0	7+2=9
0	0	0	0	11+3=14
3,5	1	2	1	10+2=12
3	1	2	1	6+1=7
1	1	1	1	6+2=8
1	1	2	1	8+3=11
1	0	2	1	10+3=13
1	1	1	1	11+2=13
2	1	1	1	9+2=11
0	0	0	0	10+2=12
0	0	0	0	2+1=3
2	1	2	1	5+2=7
1	1	1	1	4+2=6
1	1	1	1	4+1=5
1,5	1	1	1	8+2=10
3	1	1	1	6+2=8
1,5	1	2	1	9+2=11
2,5	1	2	1	11+2=13
0	0	0	0	10+2=12
5	1	2	1	9+2=11
1	1	1	1	4+2=6
0	0	0	0	6+1=7
3	1	2	1	7+2=9
2	1	2	1	11+3=14
2	1	2	1	5+2=7
1	1	2	1	9+3=12
1	1	1	1	11+3=14
0	0	0	0	10+4=14
1	1	1	1	7+2=9

2	1	2	1	6+1=7
2	1	2	1	8+3=11
1,75	1	1	1	10+2=12
2	1	1	1	8+2=10
2	0	0	1	5+2=7
2	1	2	1	10+3=13
0	0	0	0	8+2=10
7	1	2	1	9+2=11
1	1	2	1	9+2=11
2	1	2	1	8+3=11
0	0	0	0	6+1=7
0	0	0	0	5+1=6
1	1	1	1	7+2=9
1	1	1	1	8+3=11
1	1	1	1	6+2=8
8	1	2	1	6+1=7
1	1	1	1	9+3=12
1	1	2	1	9+2=11
2	1	2	1	6+2=8
1	0	2	1	8+2=10
2	1	1	1	11+2=13
0	0	0	0	11+3=14
1	1	1	1	10+2=12
2	1	2	1	10+2=12
2	0	2	1	12+2=14
1	1	1	1	9+3=12
1	1	1	1	5+2=7
0	0	0	0	6+1=7
2	1	1	1	5+2=7
0,5	0	2	1	9+2=11
0	0	0	0	10+2=12
0	0	0	0	11+4=15
2	1	1	1	9+3=12
1	1	1	1	9+2=11
4,5	1	2	1	7+2=9
3,5	1	2	1	10+3=13
1	1	1	1	11+3=14
0	0	0	0	5+2=7
1	1	2	1	7+2=9
0,5	1	1	1	9+2=11
2	1	1	1	6+1=7
3	1	2	1	8+2=10
1,5	1	1	1	9+3=12
2	1	1	1	10+4=14
8	1	2	1	9+3=12
1	1	1	1	4+2=6
0	0	0	0	11+3=14
1	1	1	1	11+3=14
0	0	0	0	8+2=10
0	0	0	0	10+2=12

2	1	1	1	7+2=9
1	1	1	1	11+3=14
1	1	1	1	12+4=16
0	0	0	0	7+2=9
1	1	1	1	11+3=14
2	1	2	1	8+3=11
1	0	0	1	11+3=14
1	1	2	1	14+4=18
1,5	1	1	1	7+2=9

Frailtya1	Frailtyb1	Frailtyc1	Handkraft re1a	Handkraft re 1b
12	2	14	50	50
11	2	13	50	50
11	3	14	75	70
9	2	11	50	55
11	4	15	70	50
12	3	15	15	10
10	2	12	60	60
8	1	9	40	35
8	3	11	40	60
8	3	11	45	45
8	2	10	45	40
9	3	12	35	30
6	3	9	25	30
15	4	19	25	25
9	3	12	50	50
12	3	15	35	30
7	2	9	10	8
11	3	14	40	40
10	3	13	60	55
13	3	16	2	1
9	2	11	58	50
8	2	10	45	40
11	3	14	60	55
8	2	10	70	65
11	3	14	45	54
8	2	10	40	40
7	2	9	48	40
7	2	9	60	58
12	3	15	42	42
11	3	14	45	54
12	2	14	25	25
8	2	10	40	35
10	2	12	30	30
9	2	11	15	20
9	2	11	18	20
5	2	7	34	34
10	2	12	26	24
14	2	16	50	48
8	3	11	35	30
10	2	12	40	30
6	2	8	72	70
8	2	10	24	36
12	2	14	26	20
10	2	12	0	0
9	2	11	28	32
8	3	11	50	45

10	2	12	30	35
13	3	16	50	45
10	2	12	40	40
13	4	17	10	10
12	4	16	30	30
8	2	10	14	16
9	2	11	58	58
13	2	15	58	60
12	4	16	70	60
13	2	15	6	8
8	2	10	45	45
8	1	9	50	50
6	2	8	35	30
7	2	9	65	60
5	1	6	90	85
10	3	13	20	15
8	1	9	40	38
3	1	4	75	65
12	4	16	45	45
9	3	12	30	35
5	2	7	60	60
7	2	9	30	25
11	3	14	55	50
10	2	12	35	33
6	1	7	58	55
6	2	8	40	40
8	3	11	20	12
10	3	13	10	10
11	2	13	45	42
9	2	11	50	60
10	2	12	40	30
2	1	3	90	85
5	2	7	48	45
4	2	6	55	60
4	1	5	44	46
8	2	10	46	54
6	2	8	50	50
9	2	11	20	18
11	2	13	0	0
10	2	12	30	20
9	2	11	44	44
4	2	6	40	40
6	1	7	60	45
7	2	9	45	40
11	3	14	20	40
5	2	7	20	20
9	3	12	50	50
11	3	14	40	40
10	4	14	20	25
7	2	9	30	30

6	1	7	60	60
8	3	11	65	60
10	2	12	30	30
8	2	10	60	60
5	2	7	40	40
10	3	13	10	10
8	2	10	40	40
9	2	11	50	45
9	2	11	50	45
8	3	11	40	40
6	1	7	20	25
5	1	6	70	65
7	2	9	40	40
8	3	11	50	45
6	2	8	30	30
6	1	7	60	60
9	3	12	40	30
9	2	11	60	60
6	2	8	53	63
8	2	10	50	50
11	2	13	35	40
11	3	14	20	20
10	2	12	70	72
10	2	12	35	30
12	2	14	60	55
9	3	12	25	27
5	2	7	20	10
6	1	7	65	65
5	2	7	14	18
9	2	11	24	20
10	2	12	2	0
11	4	15	35	40
9	3	12	28	22
9	2	11	30	40
7	2	9	30	20
10	3	13	18	18
11	3	14	40	48
5	2	7	92	88
7	2	9	86	82
9	2	11	45	40
6	1	7	48	40
8	2	10	70	68
9	3	12	17	19
10	4	14	45	50
9	3	12	30	20
4	2	6	10	20
11	3	14	10	10
11	3	14	8	0
8	2	10	52	52
10	2	12	35	30

7	2	9	70	70
11	3	14	10	10
12	4	16	40	40
7	2	9	35	40
11	3	14	40	35
8	3	11	40	40
11	3	14	20	21
14	4	18	12	10
7	2	9	30	30

Handkraft li 1a	Handkraft li1b	HADS 2	HADS2-Angst
40	40	14	5
50	50	11	6
70	70	9	4
40	40	6	5
20	20	9	2
40	40	16	7
60	60	13	3
30	30	16	5
30	25	8	1
48	40	4	3
45	40	6	4
40	40	9	3
32	32	15	10
30	35	18	1
35	35	5	3
35	35	20	8
10	14	2	1
30	30	4	0
65	50	9	3
1	0	18	7
50	50	3	2
60	55	7	4
35	35	6	1
65	60	10	2
75	65	12	5
38	43	2	1
42	41	9	8
75	70	5	0
35	35	11	3
48	60	16	2
22	40	1	1
45	60	10	1
30	28	5	2
35	38	15	4
38	30	7	0
55	45	4	0
30	32	17	3
40	40	17	8
80	75	5	1
20	20	18	9
0	0	10	3
22	20	12	6
20	10	20	11
60	56	9	4
30	26	16	7
35	40	29	15

40	40	6	1
40	38	2	0
40	35	25	15
0	0	19	6
28	25	25	13
2	6	10	6
60	56	22	6
55	55	21	8
10	5		
16	12		
50	50	18	7
50	30	31	11
30	30	9	4
25	25	24	7
70	65	13	6
0	0	35	21
28	28	16	12
60	55	7	3
40	40	29	12
50	45	22	13
90	90	29	12
25	20	28	13
47	40	31	13
45	32	33	17
60	54	16	5
50	40	16	8
12	10	17	6
0	0	18	6
42	38	32	16
30	20	37	18
10	12	32	15
80	72	12	8
40	45	22	8
88	88	12	4
54	50	19	11
54	53	24	11
50	46	27	7
0	0	18	5
0	1	17	6
20	30	32	16
38	34	26	11
40	40	18	6
60	50	14	6
35	30	33	15
50	30	32	6
30	35	12	4
35	35	10	2
35	30	17	8
0	0	13	6
33	28	13	0

65	65	28	12
35	35	23	3
50	35	34	17
30	30	23	5
35	30	15	9
5	8	13	6
45	45	34	14
50	50	30	13
45	40	18	7
20	20	16	3
25	25	16	7
70	70	8	7
20	10	11	5
58	50	23	10
50	45	31	14
65	60	27	13
0	0	14	5
65	60	31	13
40	55	19	8
70	75	27	9
40	42	19	4
15	15	39	18
80	78	21	7
20	20	15	4
35	30	19	5
20	23	16	3
10	5	9	7
60	50	35	16
55	55	21	8
30	26	31	12
0	2	28	12
38	48	11	4
22	18	37	17
36	40	18	3
20	18	25	11
14	10	30	12
54	64	8	4
64	58	18	11
44	42	16	7
30	30	18	7
42	46	15	3
65	50	38	17
29	25	24	9
40	40	13	5
35	30	24	7
35	50	11	2
3	4	13	4
0	0	33	12
70	70	10	5
20	15		

65	60
10	10
0	0
60	70
15	15
32	40
48	47
2	1
20	25

HADS 2 gesamt je 0-3 Punkte wie an Rand	HADS2-Angst Summe Fragen 1,3,5,7,9,11,13
8	0
23	8
25	10
14	6
29	16
21	6
21	7
13	5
10	6
21	9
26	5
21	5
18	5
19	5
22	10
14	8
22	13
18	10
12	3
14	9
22	7
31	14
19	10
24	9
33	14
23	7
8	8
38	19
15	5
37	17
14	7
39	18
16	4

16	3
19	7
14	8
16	3
25	8
10	6
14	8
19	12
30	11
15	4
17	6
22	9
19	9
25	13
15	6
19	8
22	7
15	6
19	8
9	5
14	6
34	13
26	5
19	6
30	11
30	12
14	4
19	6
27	10
10	2
21	8
15	9
24	8
25	8
13	7
11	5
12	6
18	11
18	5
15	7
24	9

28	16
19	5
27	11
21	8
35	16
17	9

Responder	Remitter	WohnAnders?	Wohnsituation2	Pflegeheim2
1	0	0	1	0
1	1	0	1	0
1	1	0	1	0
1	1	0	1	0
1	1	0	4	1
1	0	0	2	0
0	0	0	2	0
1	0	0	1	0
0	1	1	4	1
1	1	0	2	0
1	1	0	2	0
0	1	0	2	0
0	1	0	2	0
0	0	1	4	1
1	1	0	1	0
0	0	0	2	0
1	1	0	2	0
1	1	0	1	0
0	1	0	1	0
1	0	0	1	0
1	1	0	2	0
1	1	0	1	0
0	1	0	1	0
1	0	1	1	0
1	0	0	2	0
1	1	0	1	0
1	0	0	4	1
0	1	0	1	0
1	0	0	1	0
1	0	0	4	1
1	1	0	2	0
1	0	0	1	0
1	1	0	5	0
0	0	0	1	0
1	1	0	1	0
0	1	0	2	0
0	0	0	1	0
0	0	0	1	0
0	1	0	2	0
1	0	0	1	0
0	1	0	1	0
0	0	0	1	0
1	0	0	1	0
1	0	0	1	0
0	0	0	2	0
0	0	0	3	0

1	1		0	2	0
1	1		1	4	1
0	0		0	2	0
0	0		0	2	0
1	0		1	4	1
1	0		0	2	0
0	0		0	1	0
0	0		1	4	1

0	0		0	1	0
0	0		0	1	0
0	1		0	1	0
0	0		0	1	0
0	1		1	4	1
0	0		0	1	0
1	1		0	1	0
0	1		0	1	0
0	0		1	4	1
0	0		0	1	0
0	0		0	1	0
0	0		0	2	0
0	0		0	1	0
0	0		0	1	0
0	0		0	1	0
0	0		0	1	0
0	0		1	2	0
0	0		0	1	0
0	0		0	1	0
0	0		0	4	1
0	0		0	1	0
0	0		0	1	0
0	0		0	1	0
0	0		1	4	1
0	0		1	4	1
0	0		0	1	0
0	0		0	2	0
0	0		0	4	1
1	0		1	4	1
0	0		0	1	0
1	0		0	2	0
0	0		0	4	1
1	0		0	1	0
0	0		0	1	0
0	0		0	1	0
0	0		1	4	1
0	0		0	4	1
0	0		0	1	0
0	1		0	1	0
1	0		0	2	0

0	0	0	2	0
0	0	0	1	0
0	0	0	2	0
0	0	1	4	1
0	0	0	2	0
0	0	0	4	1
0	0	0	1	0
0	0	0	0	0
0	0	0	1	0
1	0	0	2	0
0	0	0	2	0
0	0	0	1	0
0	0	1	4	1
0	0	0	1	0
0	0	0	1	0
0	0	0	4	1
0	0	0	1	0
0	0	0	2	0
0	0	0	1	0
0	0	0	2	0
0	0	0	1	0
0	0	0	2	0
0	0	1	4	1
1	0	0	1	0
0	0	0	4	1
0	0	0	4	1
0	0	0	1	0
0	0	1	4	1
0	0	1	4	1
0	0	0	1	0
0	0	0	1	0
0	1	1	4	1
0	0	0	2	0
0	0	0	2	0
0	0	0	2	0
0	0	0	1	0
1	0	1	4	1
0	0	0	4	1
0	0	0	1	0
0	0	1	4	1
0	0	0	1	0
0	0	0	1	0
0	0	0	1	0
0	0	0	4	1
0	0	0	4	1
0	0	0	2	0
0	0	0	1	0
0	0	0	1	0
0	0	0	1	0

	ResponderT2	RemitterT2
ı	mind	
3	4 Punkte weni	Gesamtpunkte<8
	in HADS, ja=1	in HADS, ja=1 nein=0
	1	0
	0	0
	0	0
	0	0
	0	0
	0	0
	0	0
	1	0
	0	0
	0	0
	0	0
	0	0
	0	0
	0	0
	1	0
	0	0
	0	0
	0	0
	0	0
	0	0
	0	0
	0	0
	0	0
	0	0
	0	0
	0	0
	0	0
	0	0
	0	0
	0	0
	0	0
	0	0
	0	0

0	0
0	0
0	0
0	0
0	0
0	0
0	0
0	0
0	0
0	0
0	0
0	0
0	0
0	0
0	0
0	0
0	0
0	0
1	0
0	0
0	0
0	0
0	0
1	0
0	0
0	0
0	0
0	0
1	0
0	0
0	0
0	0
0	0
0	0
0	0
1	0
0	0
1	0
0	0
0	0
0	0

0	0
0	0
0	0
0	0
0	0
0	0

Pflegestufe2	Ressource util2- Pat stationär	Ressource util2- Notaufnahme
1	0	0
1	1	0
0	1	0
0	1	0
3	1	0
2	1	0
0	1	0
0	1	0
1	1	0
1	0	0
0	0	0
	0	0
0	0	0
1	0	1
0	1	0
1	1	0
0	0	0
1	0	0
0	0	0
1	1	1
0	1	1
0	1	0
	1	0
0	0	0
1	0	0
0	1	0
1	0	0
0	0	0
1	0	0
	1	0
0	1	0
0	0	0
1	1	1
0	1	0
1	0	0
1	0	0
0	1	0
0	0	0
1	0	0
0	1	0
1	0	0
0	1	0
1	0	0
1	1	0
0	0	0
	1	0

1	1	0
1	1	0
0	0	0
2	1	0
2	0	0
1	0	1
1	0	0
0	0	0

0	0	0
0	0	1
1	0	0
0	0	0
0	0	0
1	0	0
1	0	0
0	1	0
2	0	0
1	0	0
0	1	0
0	1	0
1	1	1
0	1	1
0	1	1
1	1	0
2	0	0
1	0	
0	0	0
1	0	1
1	0	0
0	0	0
0	1	
1	1	1
1	1	0
0	0	0
0	0	1
1	0	0
0	1	0
1	0	0
0	1	0
1	0	1
0	0	0
1	1	1
1	0	0
1	0	1
0		
2	0	0
2	0	0
0	0	0

0	0	0
1	0	0
2	0	
1	0	0
1	1	0
2	1	1
1	0	0
1	0	0
1	0	0
1	1	0
1	1	1
0	0	0
1	0	0
2	0	0
2	0	0
	0	0
2	0	0
0	1	1
1	1	0
0	0	0
0	1	0
2	1	1
1	1	1
0	0	0
1	0	0
1	0	1
1	1	0
1	0	0
2	0	0
1	0	0
1	0	0
1	1	1
3	1	1
1	1	0
2	1	0
0	1	0
1	0	0
0	1	
1	0	0
1	1	0
1	0	0
3	1	0
1	1	0
3	1	1
2	1	0
1	0	0
	1	1
3	0	0
	1	0

Ressource util2- Anzahl Notaufnahme	Stürze2	Barthel2	Antidepressiva?2	Seit wann AD?2
0	1	95	1	2
0	0	75	1	2
0	1	95	1	1
0	1	90	1	1
0	1	30	0	0
0	1	55	1	1
0	1	100	1	1
0	1	90	0	0
0	0	75	1	1
0	0	90	1	2
0	1	100	0	0
0	1	95	1	1
0	1	90	0	0
1	1	25	1	1
0	1	100	1	2
0	1	75	1	2
0	1	95	0	0
0	0	80	1	1
0	1	85	0	0
1	0	85	1	2
1	0	95	1	3
0	1	100	0	0
0	1	90	1	1
0	0	90	1	2
0	1	100	1	2
0	0	95	1	1
0	1	95	1	2
0	1	100	1	1
0	0	70	0	0
0	1	65	1	1
0	0	95	1	2
0	0	100	0	0
1	0	95	1	2
0	0	65	0	0
0	1	70	1	1
0	1	85	1	2
0	1	90	1	2
0	0	75	1	2
0	0	70	0	0
0	0	80	1	1
0	0	80	1	1
0	1	95	1	2
0	0	90	1	2
0	1	90	1	2
0	1	90	1	1
0	1	75	1	2

0	1	75	1	3
0	1	55	0	0
0	1	85	1	2
0	0	30	1	2
0	1	20	1	1
1	1	85	1	1
0	1	70	0	0
0	0	80	0	0
0	0	80	0	0
16	1	85	0	0
0	0	90	1	2
0	0	65	0	0
0	0	90	1	2
0	0	60	0	0
0	1	95	0	0
0	0	95	0	0
0	1	40	1	1
0	0	55	0	0
0	0	100	1	3
0	1	95	1	3
1	1	60	0	0
3	1	75	1	2
1	1	90	1	2
0	1	85	1	1
0	1	45	1	2
1	0	55	0	0
0	0	90	1	1
1	0	90	1	1
0	1	45	1	3
0	1	95	0	0
0	0	75	1	1
3	0	60	1	1
0	0	80	0	0
0	1	75	1	1
1	0	20	1	1
0	1	70	1	2
0	1	95	1	2
0	1	80	0	0
0	1	80	1	3
1	1	80	1	3
0	1	90	0	0
3	0	80	1	2
0	0	70	1	2
1	0	85	0	0
	1	85	1	2
0	1	30	0	0
0	0	10	0	0
0	0	85	1	1

0	0	90	1	1
0	1	65	1	2
	0	30	1	1
0	1	80	0	1
0	1	8	0	0
1	0	50	1	2
0	0	60	1	3
0	1	30	1	2
0	1	75	1	2
0	1	75	1	2
1	1	60	0	0
0	1	100	0	0
0	0	60	1	1
0	0	30	1	1
0	1	55	1	1
0	1	95	1	2
0	1	35	1	3
4	1	60	1	1
0	1	85	1	2
0	0	85	0	2
0	0	75	1	3
1	0	25	0	0
2	0	65	1	3
0	0	90	0	2
0	0	70	0	2
1	1	65	0	0
0	1	80	1	1
0	0	35	0	0
0	1	45	1	1
0	1	55	0	2
0	1	80	0	0
1	0	85	0	0
3	0	10	1	3
0	1	65	1	1
0	0	75	1	2
0	1	40	1	2
0	1	65	1	1
	1	80	0	0
0	0	70	1	2
0	1	70	1	3
0	0	65	1	1
0	0	5	1	3
0	0	25	0	2
1	1	15	1	1
0	0	15	1	2
0	0	40	1	1
1	0	40	0	0
0	1	0	1	1
0	1	90	0	0

Barthel2

30
75
60
90
90
70
90
30
100
85
40
70
20
20
55
100
80
50
60
80
60
70
90
55
30
35
95
10
80
50
70
10
90

95
60
90
85
70
40
70
85
35
80
75
40
80
85
65
85
60
85
85
60
60
0
70
75
15
50
30
85
100
75
80
90
55
90
90
70
75
50
75
95
75

60
65
0
95
75
85

Psychopharm aka	MEL-life events2	PSP2	Frailty-Index2
1	0	61	5+2=7
1	84	60	9+3=12
1	37	70	2+1=3
1	53	80	4+2=6
1	53	30	5+4=9
1	53	70	5+2=7
1	82	50	5+2=7
0	53	70	5+1=6
1	147	61	5+3=8
1	97	71	4+1=5
1	53	80	5+1=6
1	53	70	5+2=7
0	97	70	4+2=6
1	134	30	11+3=14
1	53	50	4+3=7
1	53	30	9+3=12
1	53	71	4+1=5
1	53	50	4+3=7
0	209	60	4+2=6
1	53	51	10+3=13
1	147	70	4+2=6
0	44	70	4+1=5
1	53	50	5+3=8
1	173	70	5+2=7
1	115	80	5+2=7
1	53	80	4+2=6
1	53	41	2+2=4
1	53	60	7+2=9
0	172	50	8+2=10
1	72	50	11+3=14
1	118	70	3+2=5
0	72	60	5+2=7
1	234	80	7+2=9
0	97	60	9+2=11
1	96	30	7+2=9
1	72	61	4+2=6
1	53	50	7+1=8
1	53	70	12+2=14
0	53	51	6+3=9
1	53	71	8+2=10
1	53	70	6+2=8
1	53	70	8+2=10
1	72	70	8+2=10
1	90	80	4+1=5
1	53	41	8+2=10
1	53	21	9+2=11

1	53	60	6+2=8
0	134	51	7+3=10
1	97	41	9+2=11
1	53	30	10+3=13
1	111	30	12+3=15
1	72	71	6+2=8
0	71	70	9+2=11
0	209	31	11+3=14

0	96	51	9+2=11
1	90	51	10+2=12
1	37	51	6+2=8
0	77	61	7+2=9
1	73	71	5+2=7
0	111	41	10+3=13
0	130	61	8+1=9
0	134	61	6+2=8
1	197	21	14+4
1	96	21	11+3=14
1	115	41	8+2=10
1	134	60	9+2=11
0	95	30	13+3=16
1	127	51	11+2=13
1	121	51	7+2=9
1	53	41	11+2=13
1	243	30	12+3=15
0	53	31	10+3=13
1	100	41	10+2=12
1	189	21	11+2=13
1	53	20	14+3=17
0	53	60	4+1=5
1	96	50	9+3=12
1	234	41	9+3=12
1	287	61	12+2=14
1	116	50	10+2=12
1	130	21	8+3=11
1	53	31	10+3=13
1	141	41	10+2=12
0	225	41	11+2=13
1	77	51	10+2=12
1	63	51	8+2=10
0	116	51	7+2=9
1	136	21	11+3=14
1	53	50	11+3=14
1	122	41	11+3=14
1	0	30	10+3=13
0	82	21	13+3=16
0	76	21	10+4=14
1	90	51	3+2=5

1	139	21	6+2=8
1	53	50	9+3=12
1	77	21	15+3=18
1	186	21	10+3=13
0	82	41	6+2=8
1	147	21	11+3=14
1	141	30	10+3=13
1	114	20	12+3=15
1	114	30	9+2=11
1	136	30	9+3=12
0	53	41	10+3=13
0	44	80	4+1=5
1	171	51	6+2=8
1	109	21	10+4=14
1	134	21	10+3=13
1	53	61	7+2=9
1	53	30	10+3=13
1	172	20	11+3=14
1	90	51	9+2=11
1	71	41	8+2=10
1	173	30	12+2=14
0	127	21	13+3=16
1	172	30	10+3=13
1	71	60	7+2=9
1	144	41	12+3=15
0	53	21	12+3=15
1	77	50	4+2=6
0	234	21	13+3=16
1	190	30	13+3=16
1	90	40	11+3=14
0	72	31	10+2=12
0	205	50	8+3=11
1	114	21	12+4=16
1	96	41	10+2=12
1	72	51	10+2=12
1	53	21	13+3=16
1	215	31	11+3=14
0	72	51	9+2=11
1	71	40	10+2=12
1	190	21	11+2=13
1	182	61	7+2=9
1	145	21	12+4=16
1	116	30	12+4=16
1	158	30	10+4=14
1	53	21	12+4=16
1	96	20	10+3=13
0	53	50	13+3=16
1	127	10	14+4=18
0	77	40	9+2=11

Frailtya2	Frailtyb2	Frailtyc2	Tinnetti2	Handkraft re2a
5	2	7	24	70
9	3	12	22	50
2	1	3	22	90
4	2	6	28	50
5	4	9	1	50
5	2	7	13	45
5	2	7	26	60
5	1	6	28	25
5	3	8	13	45
4	1	5	26	50
5	1	6	27	45
5	2	7	27	35
4	2	6	25	20
11	3	14	13	20
4	3	7	22	55
9	3	12	14	22
4	1	5	27	35
4	3	7	22	55
4	2	6	23	50
10	3	13	16	2
4	2	6	27	54
4	1	5	24	50
5	3	8	23	74
5	2	7	25	80
5	2	7	23	46
4	2	6	28	55
2	2	4	23	50
7	2	9	23	58
8	2	10	20	60
11	3	14	9	52
3	2	5	23	40
5	2	7	28	55
7	2	9	16	30
9	2	11	20	42
7	2	9	13	54
4	2	6	23	32
7	1	8	28	22
12	2	14	28	44
6	3	9	13	50
8	2	10	18	40
6	2	8	27	74
8	2	10	21	36
8	2	10	15	42
4	1	5	26	0
8	2	10	20	30
9	2	11	11	45

6	2	8	24	70
7	3	10	5	20
9	2	11	16	45
10	3	13	1	35
12	3	15	3	28
6	2	8	19	16
9	2	11	20	60
11	3	14	17	60
		0		
		0		
9	2	11	16	50
10	2	12	27	45
6	2	8	18	50
7	2	9	9	70
5	2	7	20	75
10	3	13	4	32
8	1	9	24	44
6	2	8	18	85
14	4	18	1	38
11	3	14	4	40
8	2	10	25	60
9	2	11	15	46
13	3	16	15	40
11	2	13	12	35
7	2	9	13	60
11	2	13	14	50
12	3	15	8	40
10	3	13	12	5
10	2	12	18	68
11	2	13	27	40
14	3	17	16	30
4	1	5	24	86
9	3	12	21	20
9	3	12	12	50
12	2	14	14	28
10	2	12	7	30
8	3	11	1	35
10	3	13	12	16
10	2	12	19	28
11	2	13	12	48
10	2	12	12	26
8	2	10	21	15
7	2	9	19	40
11	3	14	10	45
11	3	14	17	48
11	3	14	6	40
10	3	13	20	60
13	3	16	11	45
10	4	14	1	12
3	2	5	15	25

6	2	8	17	60
9	3	12	3	60
15	3	18	3	10
10	3	13	10	50
6	2	8	16	30
11	3	14	8	34
10	3	13	11	30
12	3	15	10	30
9	2	11	8	50
9	3	12	12	45
10	3	13	17	4
4	1	5	26	30
6	2	8	16	40
10	4	14	0	50
10	3	13	14	40
7	2	9	24	40
10	3	13	3	40
11	3	14	15	10
9	2	11	18	50
8	2	10	20	45
12	2	14	12	15
13	3	16	0	5
10	3	13	10	60
7	2	9	19	20
12	3	15	13	55
12	3	15	13	25
4	2	6	13	20
13	3	16	4	32
13	3	16	3	5
11	3	14	16	20
10	2	12	12	18
8	3	11	10	62
12	4	16	0	0
10	2	12	10	10
10	2	12	22	30
13	3	16	10	10
11	3	14	18	68
9	2	11	20	70
10	2	12	20	32
11	2	13	11	35
7	2	9	9	40
12	4	16	0	2
12	4	16	1	20
10	4	14	0	60
12	4	16	5	2
10	3	13	3	15
13	3	16	4	20
14	4	18	0	0
9	2	11	18	58

Handkraft re 2b	Handkraft li 2a	Handkraft li2b	HADS 3
80	65	60	12
40	45	43	14
80	65	60	4
60	50	45	17
55	20	25	10
45	50	45	16
60	40	40	14
30	15	30	19
55	15	10	7
50	40	40	4
50	45	45	4
30	30	30	31
20	40	40	18
10	20	10	12
50	60	55	25
25	5	10	19
20	28	24	7
75	50	45	3
52	50	52	9
4	4	6	26
54	74	80	1
60	80	70	5
70	48	48	5
75	78	76	4
54	65	68	9
55	50	50	4
50	45	45	5
58	48	58	3
52	70	90	26
40	42	48	10
38	54	54	
64	40	60	12
32	30	32	9
38	30	42	17
50	40	46	14
30	48	44	5
22	30	34	19
38	34	36	19
48	70	66	7
30	28	18	30
74	6	2	8
38	18	18	2
42	24	26	19
0	48	38	10
30	32	32	11
40	35	35	

70	45	40	
18	10	20	
40	40	40	26
35	0	0	18
28	20	18	38
10	6	2	14
60	60	60	25
50	50	40	29
50	45	45	17
40	30	30	17
60	40	40	7
75	30	35	22
80	70	70	17
28	28	22	30
40	44	34	14
80	75	70	4
35	30	25	7
40	56	50	16
75	80	75	21
42	30	34	17
45	38	36	27
40	28	35	15
60	72	68	
55	50	50	15
40	25	20	12
0	0	0	2
66	56	56	30
40	20	20	8
20	10	5	19
90	72	68	4
20	40	42	23
50	60	50	7
30	50	48	6
30	30	35	14
34	38	36	17
20	8	10	21
26	16	14	30
45	28	20	32
28	22	20	8
20	5	8	
40	35	45	18
45	45	45	26
55	60	60	36
50	50	50	34
60	70	65	14
45	45	45	18
10	0	0	18
20	15	18	20

55	60	55	24
60	30	30	18
5	5	5	33
50	25	15	24
30	4	4	14
34	22	20	11
28	18	20	33
30	35	30	37
45	35	25	21
40	20	20	15
4	2	0	11
60	60	50	8
30	20	10	8
46	60	60	28
35	35	30	17
40	40	30	22
40	18	22	9
14	30	26	35
50	40	45	25
65	65	75	18
14	25	24	12
0	0	0	26
56	50	54	14
20	22	22	14
55	30	30	19
25	20	20	17
20	10	10	12
30	45	35	37
3	30	20	16
15	20	20	37
10	2	4	20
60	60	60	18
0	0	0	17
10	30	30	24
28	32	26	21
10	5	0	15
66	50	70	9
65	50	50	21
18	30	20	25
30	30	20	22
40	52	50	
0	0	0	
16	4	2	
60	45	48	
0	10	10	
10	33	33	
20	30	30	
0	0	0	
50	60	58	

HADS 3 gesamt je 0-3 Punkte wie an Rand
12
23
26
31
27
22
15
30
21
25
32
11
21
13
12
40
25
21
21
11
18
21
14

19
13
14
36

12
22
13

13
15
22
23
18
13
13
15

15
4
13
34
28
31
28
21
15
25

10

35

26
11

30
18
24

15

19
27
14
33
15

HADS3-Depr	HADS3-Angst	HAMD 3-gesamt	Responder3	Remitter3	WohnAnders 3
8	4	10	1	0	0
6	8	10	1	0	0
3	1	3	1	1	0
9	8	12	0	0	0
9	1	4	1	0	0
8	8	7	0	0	0
10	4	9	0	0	0
12	7	10	0	0	0
5	2	5	1	1	0
2	2	11	1	1	0
2	2	5	1	1	0
20	11	23	0	0	0
7	11	12	0	0	0
10	2	5	0	0	0
14	11	27	1	0	0
14	5	17	0	0	0
2	5	11	1	1	0
3	0	4	1	1	0
4	5	7	1	0	0
15	11	26	0	0	0
1	0	3	1	1	0
2	3	4	1	1	0
3	2	8	1	1	0
4	0	8	1	1	0
6	3	12	0	0	0
1	3	1	1	1	0
1	4	4	1	1	0
3	0	7	1	1	0
12	14	15	0	0	0
10	0	7	1	0	1
			1	1	0
8	4	4	0	0	0
6	3	9	1	0	0
8	9	9	1	0	0
10	4	6	0	0	0
3	2	3	1	1	0
14	5	14		0	0
10	9	12		0	0
3	4	5		1	0
16	14	24		0	0
7	1	5		0	0
1	1	1	1	1	0
12	7	16	0	0	0
8	2	12		0	0
6	5	10		0	0
			1	1	

				1	
			1	1	
12	14	28	0	0	0
13	5	10	0	0	0
21	17	30		0	0
3	9	14		0	0
16	9	17	0	0	0
17	12	25	0	0	1
				1	
			1	1	
7	10	17	0	0	1
10	7	21	1	0	0
5	2	5	1	1	0
14	8	15	0	0	0
6	11	11	1	0	0
14	16	30	0	0	0
7	7	14	1	0	0
1	3	2	1	1	0
6	1	5	1	1	1
7	9	10	0	0	0
8	13	13	1	0	0
9	8	10	1	0	0
14	13	23	0	0	0
8	7	15		0	0
				1	0
6	9	13	0	0	0
6	6	11		0	1
2	0	2	1	1	0
15	15	23		0	0
5	3	10		0	0
14	5	11		0	0
0	4	3	1	1	0
14	9	18	0	0	0
7	0	2	1	1	0
3	3	3		1	0
13	1	8	1	0	0
14	3	12	1	0	1
11	10	17	0	0	0
14	16	17		0	0
20	12	18		0	0
5	3	14	1	0	0
			1	1	
6	12	17	0	0	0
19	7	23	0	0	1
20	16	38	0	0	0
21	13	32	0	0	0
8	6	18	0	0	0
10	8	26	0	0	0
11	7	25	0	0	0
16	4	20	0	0	0

18	6	27	0	0	0
9	9	10	0	0	0
20	13	31	0	0	1
19	5	21	0	0	0
10	4	12	1	0	0
7	4	18	1	0	1
20	13	31	0	0	0
21	16	37	0	0	0
13	8	18	0	0	0
12	3	15	0	0	0
7	4	17	0	0	1
1	7	13	1	0	0
4	4	8	0	0	0
17	11	25	0	0	0
14	3	15	0	0	0
15	7	19	0	0	0
6	3	2	1	0	0
20	15	35	0	0	0
11	14	23	0	0	0
14	4	21	0	0	0
8	4	12	0	0	1
16	10	31	1	0	1
12	2	12	0	0	0
6	8	15	1	0	0
14	5	18	0	0	0
13	4	19	1	0	0
4	8	14	1	0	0
20	17	31	0	0	0
10	6	21	0	0	0
21	16	34	0	0	0
13	7	24	1	0	0
13	5	16	1	0	0
10	7	16	0	0	0
18	6	27	0	0	0
12	9	23	0	0	0
9	6	13	0	0	0
5	4	14	0	0	0
10	11	18	0	0	0
15	10	24	0	0	0
13	9	22	0	0	0
				1	
			1	1	
				1	
			1	1	
			1	1	
			1	1	
			1	1	
			1	1	
			1	1	
			1	1	

			1	1
			1	1
			1	1
			1	1
			1	1
			1	1
			1	1
			1	1
			0	1

HADS3-Depr Summe Frage 2,4,6,8,10,12,	HADS3-Angst Summe Fragen 1,3,5,7,9,11,13	HAMD3-gesar	ResponderT3 mind 4 Punkte weni in HADS, ja=1	RemitterT3 Gesamtpunkte<8 in HADS, ja=1
10	2	10	0	0
16	7	27	0	0
15	11	20	0	0
15	16	28	0	0
16	11	16	0	0
16	6	21	0	0
8	7	16	0	0
16	14	29	0	0
13	8	22	0	0
16	9	33	0	0
20	10	28	0	0
6	5	16	0	0
11	10	28	0	0
7	6	19	1	0
9	3	14	0	0
21	19	32	0	0
18	7	26	0	0
10	11	21	0	0
11	10	17	0	0
4	7	15	0	0
11	7	18	0	0
14	7	18	0	0
8	6	14	0	0

12	7	24	0	0	
7	6	12	0	0	
9	5	16	0	0	
21	15	27	0	0	
5	7	17	0	0	
13	9	14	0	0	
6	7	13	1	0	
10	3	11	0	0	
10	5	13	0	0	
13	9	22	0	0	
13	10	19	0	0	
8	10	15	1	0	
8	5	12	0	0	
7	6	14	1	0	
11	4	14	1	0	
9	6	11	1	0	
1	3	5	1	1	
8	5	16	0	0	
21	13	41	0	0	
21	7	26	0	0	
18	13	24	0	0	
17	11	29	0	0	
12	9	16	1	0	
11	4	24	0	0	
17	8	27	0	0	
					0
8	2	12	0	0	
20	15	35	0	0	
18	8	21	0	0	
5	6	16	0	0	
18	12	23	0	0	
7	11	19	0	0	
15	10	19	0	0	
8	7	14	0	0	

5	14	19	0	0
17	10	27	0	0
8	6	20	1	0
19	14	27	0	0
7	8	10	0	0

Ressource util3- Pat stationär	Ressource util3- Notaufnahme
0	1
1	0
0	0
1	1
1	1
0	0
0	0
0	0
1	1
1	0
1	1
1	0
0	0
0	1
0	1
1	0
1	1
0	0
0	0
1	1
1	0
0	0
1	1
1	0
0	0
1	0
1	0
0	0
0	0
0	0
0	0
1	0
0	0
1	0
0	0
0	0
0	0
0	0
0	0
0	0
0	0
0	0
0	0
0	0
0	0

0	0
1	0
0	0
0	1
1	1
0	0

1	1
1	1
0	0
0	0
0	0
0	1
0	0
0	0
1	0
0	1
0	0
1	1
1	0
0	0
1	0
1	0
0	0
0	1
0	0
0	0
1	0
0	0
0	1
1	1
0	0
1	0
1	0
1	0
1	1
1	0
1	0

0	0
0	0
0	0
0	1
1	1
1	1
0	1
1	0

0	0
0	0
0	0
0	0
1	0
0	1
0	0
0	0
0	0
0	0
1	1
1	1
0	0
1	1
0	0
1	1
0	0
1	1
1	0
0	
0	0
0	0
1	0
0	0
0	0
0	0
0	1
0	0
0	0
0	0
0	0
0	1
1	0
0	0
0	0
0	0
0	0
1	1
1	1
1	0

0 0

Ressource util3- Anzahl Notaufnahme	Ressource util3- Art Pflegedienst	Schmerz3	Stürze3	Zahl Sturz3
1	1,2	1	0	3
0	1,2	1	0	1
0	1	1	1	0
0,5	1,2	0	1	0
0,5	6	1	1	0
0	1,2,5	0	0	1
0		1	0	1
0		1	0	2
0,5	6			
0	2	0	0	5
1		1	0	1
0		1	1	0
0	1	0	0	1
0,5	6	1	1	0
0,5	7	1	0	4
0	1	0	1	0
0,5	1	0	1	0
0		0	0	1
0	7	1	1	0
1	1,2	0	0	4
0		0	1	0
0		1	1	0
0,5	1,2,3	1	1	0
0		1	1	0
0	1	1	0	1
0		1	1	0
0	6	1	0	1
0	2	1	0	3
0		0	1	0
0	6	1	0	2
0		1	0	4
0		1	0	1
0	1,2	0	0	1
	1,3,	0	0	3
0	7	0	1	0
0	1,2,	0		
0	1,2	1	1	0
0	1	1	0	8
0	1	0	1	0
0		0	1	0
0	2	1	0	2
0	1,2,	1	1	0
0	1	0	0	6
0	1	0	1	0
0	1,2	0	0	1

0			1	0
0	1,7	1	1	0
0	6	0	0	1
0,5	1,2	0	1	3
0,5	2.3,	0	0	1
0	6	0	1	0
0,5	6	0	0	2
4		0	1	0
0	1,2	1	1	0
0	1	0	0	3
0		0	0	3
1	2	0	0	3
0	2	1	1	0
0	2	1	1	0
0	6	1	1	0
1		0	0	3
0		1	0	1
0,5		1		
0	7	1	1	0
0	7	0	1	0
0		0	1	0
0	1,2	0	0	1
0		0	0	3
0,5	1,2,	0	0	8
0	1,2	0	0	1
0	6	1	1	0
0		0	1	0
0	2	1	1	0
0,5	3	0	0	8
1	6	1	0	3
0	6	0	0	1
0	2,8,	1	1	0
0	6	0	0	2
0	6	0	1	0
1	1,2,3	0	1	0
0		1	0	2
0	1,2,	0	0	1
0	1,2	1	1	0
0	7	0	0	1
0	1,2	0	0	3
0,5	6	0	0	1
0,5	1	0	0	1
1,5	1	0	0	3
0,5	7	1	0	1
0	1,2,3,5	0	0	1

0		1	1	0
0	1	0	0	1
0	6	0	0	3
0	6	1	1	0
0	1,2	0	0	1
0,5	1	0	1	0
0		0	1	0
0	7	0	1	0
0	2	0	0	1
0	6	1	0	1
0,5	6	0	1	0
1x	0	0	1	0
0	6	1	0	1
0,5	1	1	0	22 (?)
0	1	1	0	2
	6	0	1	0
0		1	1	0
2x	1,2	0	0	
0	2	0	1	0
	1	1	0	3
0	6	0		
0	6	0	1	0
0	6	1	0	4
0	2,7	0	1	0
0	6	0	1	0
0	6	1	0	1
2x	2,3	0	0	2
0	6	0	0	2
0	6	1	1	0
0	1,2	0	1	0
0	2	0	1	0
0,5	6	0	0	3
0	1	1	1	0
0	1,2,3	0	1	0
0	1,2,4	1	1	0
0	1,2	0	1	0
0	6	0	1	0
1	1,2	1	0	6
0,5	7	0	1	0
0	6	0	0	1

0	0	0	1	0

Tinnetti3	Handkraft re3a	Handkraft re 3b	Handkraft li 3a	Handkraft li3b
	48	48	58	58
	60	70	80	70
	90	70	50	35
22	54	50	44	34
	46	48	14	16
	20	20	42	48
	50	55	50	48
	50	48	40	40
	44	40	30	26
	52	44	32	32
	48	54	48	46
1				
	26	30	38	38
	16	14	12	10
	34	16	4	8
	18	18	2	2
	4	2	16	8
	60	60	34	30
	70	68	64	62
	2	0	0	0
26	52	64	68	68
	42	40	52	52
	50	50	28	18
28	80	75	74	68
	34	36	14	14
	50	54	46	50
18	50	50	50	50
	42	48	38	38
	40	40	40	48
	38	30	52	46
21	36	40	46	38
	60	62	62	60
	30	38	30	34
	20	18	40	36
	48	40	50	50
	45	42	60	62
	30	32	24	32
	65	60	40	30
	60	62	64	62
	18	6	8	4
	68	60	0	0
	48	46	20	24
	53	55	40	45
	2	0	42	46
	45	48	38	34

		4	2	0	0
	1	15	20	15	10
	20	20	20	10	5
	18	60	50	50	40
	15	50	50	50	40
		30	30	45	45
		50	50	50	45
		50	50	60	58
		70	72	48	52
		90	100	84	84
		50	54	30	20
		54	50	34	24
		80	70	68	64
		14	16	4	2
		12	20	60	55
	14	60	80	80	70
	28	42	36	20	24
		46	46	34	36
	13	30	30	30	20
		68	62	66	64
	16	50	35	50	70
		4	2	18	16
		0	0	0	0
		56	48	28	32
28?		40	35	25	25
		46	40	20	18
		90	92	80	74
		10	10	50	50
		48	50	60	62
		40	48	50	54
	9	40	40	30	35
	2	40	35	40	35
		4	2	14	12
		28	26	26	26
	12	50	50	30	20
		38	35	30	27
	19	30	30	40	30
	10	28	25	22	14
		40	45	35	40
	0	0	0	0	0
		60	62	50	58
		42	48	36	38
		1	0	0	0
		20	20	15	15

14	50	55	55	50
	50	50	35	20
2	5	0	5	0
10	60	50	30	20
	20	20	4	2
	14	28	4	0
	15	10	2	0
4	10	5	5	5
7	50	40	40	20
10	40	30	20	10
16	2	2	2	0
26	50	50	50	45
18	40	30	30	20
1	20	18	15	15
	40	52	65	62
23	45	30	30	30
3	38	40	24	22
10	10	0	10	10
17	50	35	35	30
17	60	50	60	50
	8	5	20	15
1	20	15	15	10
	48	56	46	48
18	30	20	20	15
12	40	40	25	20
12	20	20	20	15
11	10	15	10	0
4	30	20	30	20
22	20	10	60	50
6	10	5	10	5
	15	10	0	2
8	50	35	40	35
	0		0	
8	10	10	30	25
20	30	20	30	20
11	20	20	10	10
17	60	60	50	50
17	70	60	60	50
16	30	20	20	20
9	30	30	30	20

30	20	80	80

BMI3	Waist-Hip-ratio3	Barthel3	Name	Code	Verlauf
19,9	1,01	85	Rossmanith	S01	abgeschlossen
22,7	1,08	95	Uhse	S02	abgeschlossen
26,4	0,92	95	Ziegler	S04	abgeschlossen
29,6	0,95	80	Maisch	S05	abgeschlossen
27,9	0,86	40	Fuchs	S06	abgeschlossen
32,1	0,8	85	Jägers	S07	abgeschlossen
22,2	0,97	100	Reis, Erich	S08	abgeschlossen
21,6	0,88	95	Zuber	S09	abgeschlossen
29,8	0,81	80	Imdahl	S10	abgeschlossen
29,3	0,99	95	Dischka	S13	abgeschlossen
26,1	0,89	100	Wurst	S15	abgeschlossen
14,1	0,83	5	Rupp	S16	abgeschlossen
43,1	0,97	95	Müller	S17	abgeschlossen
30,8	0,95	30	Hördt	S19	abgeschlossen
18,75	0,9	85	Hess	S20	abgeschlossen
32,5	0,95	35	Eurich	S21	abgeschlossen
34	0,97	95	Fast	S22	abgeschlossen
28,7	0,85	60	Frölich	S23	abgeschlossen
26,7	0,92	95	Sandritter	S24	abgeschlossen
35,7	0,98	35	Funk	S25	abgeschlossen
24,4	0,89	95	Kleiner	S27	abgeschlossen
23,1	0,85	95	Keuthen	S28	abgeschlossen
35	1,03	90	Mink	S29	abgeschlossen
23,2	0,94	90	Kraus	S30	abgeschlossen
25	1,03	90	Werny	S31	Ther beendet
25,3	0,93	100	Mühlbauer	S32	abgeschlossen
36,1	0,8	80	Saynisch	S33	Ther beendet
24,2	0,83		Lehre	S34	Ther beendet
27,8	0,94	80	Kaiser	S35	Ther beendet
28,5	0,97	80	Hohr	S37	Ther beendet
33,1	0,88	95	Kramer	S38	Ther beendet
21	1	95	Bender	S39	Ther beendet
30,4		85	Zeller	S40	Ther beendet
39,5		40	Friedrich	S41	Ther beendet
27,7	0,93	80	Tritz	S42	Ther beendet
29	0,87	85	Sturm	S45	Ther beendet
16	0,78	90	Aumiller	S46	Ther beendet
20,7	0,82	90	Fuhrmann	S47	Ther beendet
46,7	0,89	65	Koch	S49	Ther beendet
34,6	0,82	70	Exel	S50	Ther beendet
25,9	0,91	75	Juckenath	S51	Ther beendet
22,9	0,92	90	Erbe	S53	Ther beendet
30,5	0,91	90	Göhlich	S54	Ther beendet
30,8	0,84	85	Neidig	S55	Ther beendet
19,8	1,1	80	Lechner	S56	Ther beendet
			Borlein	S03	dropout nach

			Kessler	S12	dropout nach
			Barth	S36	Ther beendet
		70	Reis, Herta	S11	dropout
17,5	0,94	35	Fleck	S18	dropout
28,3	0,88	35	Mayrl	S26	dropout
35,2	0,98	15	Dordel	S43	dropout
25,4	0,97	85	Tetzlaff	S44	dropout
23,1	0,88	70	Klimek	S48	dropout
		75	Nohe	S14	dropout
			Schieck	S52	dropout
24,7	0,9	85	Kornmann	K04	abgeschlossen
17,1	0,78	90	Buchholz	K13	abgeschlossen
30,5	0,88	90	Schmidle	K15	abgeschlossen
34,7	0,93	70	Ludebühl	K16	abgeschlossen
23,8	0,94	95	Mederacke-M	K21	abgeschlossen
29,3	0,95	55	Ehwald	K24	abgeschlossen
20	0,99	100	Sanz	K25	abgeschlossen
39,9	0,92	95	Münd	K29	abgeschlossen
30,8	0,88	40	Stoiber	K33	abgeschlossen
24,4	1,04	70	Fleck, Ludwig	K36	abgeschlossen
25,1	1,04	95	Zimmermann,	K37	abgeschlossen
21,3	0,86	85	Teutsch	K49	dropout
36,9	0,9	75	Deyerler	K51	Ther beendet
27,6		90	Heger	K53	Ther beendet
32,3	0,83	100	Becker, Kathai	K55	Ther beendet
31,5	0,9	90	Mussler	K58	abgeschlossen
29,9		65	Kazda	K60	Ther beendet
35,7	0,89	75	Pabst	K67	Ther beendet
26,8	0,86	90	Pfeifer	K68	abgeschlossen
16,8 0,45?		95	Brandt-Resch	K73	Ther beendet
37,6	1,02	75	Schneckenber	K75	Ther beendet
25,7	0,99	100	Stutz	K78	Ther beendet
33,3	0,86	55	Boch	K79	Ther beendet
29,4	1,04	75	Herr Frets	K82	Ther beendet
20,5	0,85	75	Breitenbach	K88	Ther beendet
22,3	0,88	85	Heyne	K89	
26,1	0,93	40	Paschen	K90	Ther beendet
23,2		30	Walter	K97	Ther beendet
20,8	0,85	85	Wesch	K38	Ther beendet?
30,3	1,01	90	Gärtner	K70	?
20	0,93	100	Schork	K86	Ther
			Kusi	K07	dropout
20,7	0,59	85	Scheffner	K02	dropout
17,8	0,94	35	Simon	K05	dropout
21,5	0,86	60	Baumann	K10	dropout
14	0,82	0	Dersch	K11	dropout
43,1	1,03	85	Of	K14	dropout
32,4	0,95	30	Lang	K17	dropout
20,9	0,88	20	Kulla	K20	dropout
33,3	0,89	85	Hülzenbecher	K22	dropout

21,1		0,99	85	Rückert	K23	dropout
26		0,81	75	Hill-Weidner	K26	dropout
17,9		0,89	35	Zanier	K27	dropout
18,3		0,76	80	Wagner,A.	K28	dropout
24,2		0,87	100	Bartsch-Steger	K30	dropout
32,3		0,93	5	Wiest	K31	dropout
32,1		0,86	55	Jörder	K32	dropout
28,1		0,99	30	Emmerich	K34	dropout
22,9		0,82	50	Bischoff	K35	dropout
30,9		0,89	60	Hitzler	K39	dropout
27,3		0,93	60	Messerschmic	K40	dropout
29,7		0,88	100	Kleehaupt	K42	dropout
23,8		0,92	70	Möll	K44	dropout
24,4		1,03	35	Beisel	K45	dropout
26,7		0,85	55	Kern, Anna	K46	dropout
24,5		0,8	90	Pfandke	K47	dropout
39,5			45			
32,4		1,01	55	Bürkli	K50	dropout
23		0,99	75	Faller, renate	K52	dropout
23,2		0,91	55	Hauser	K54	dropout
28,1		0,89	45	Miltner	K56	dropout
17,4		0,85	25	Doll	K57	dropout
29,9		0,92	70	Bambach	K61	dropout
21,6		0,83	90	Michel	K63	dropout
20,4	0.93		60	Schröder	K65	dropout
23,6		0,86	65	Zimmermann,	K66	dropout
36,9		0,83	65	Franz	K74	dropout
29,4		0,91	30	Langosch	K76	dropout
24		0,82	60	Kreis	K77	dropout
18,4		0,96	35	Dentz	K80	dropout
19,9		0,85	65	Preissig	K81	dropout
17,7		0,93	65	Trunk	K83	dropout
22,6		0,93	15	Druckmann	K84	dropout
23,1		0,91	50	Beute	K87	dropout
30,1		0,96	70	Sauer	K91	dropout
33,4		1,02	55	Kress	K92	dropout
24		0,98	65	Correll	K93	dropout
25,6		0,93	75	Josse	K95	dropout
26,5		0,96	60	sperka	K98	dropout
24,5			60	Forck	K99	dropout
				Bohn	K94	?
				Wellensiek	K41	dropout
				Zepnik	K62	?
				Winterberg	K03	dropout
				Kuhlmann	K08	dropout
				Sätzler	K09	dropout
				Kirschenlohr	K12	dropout
				Heinzmann	K59	dropout
				Hambrecht	K64	dropout
				Stegmüller	K01	dropout

Wagner,Elfrie	K06	dropout
Rensch	K18	dropout
Gänzler	K19	dropout
Zehrer	K43	dropout
Kalus	K69	dropout
Fedrich	K71	dropout
Ronellenfitsch	K72	dropout
kancsar	K85	dropout
Dachner	K96	dropout

Nicolic	B01	fertig
Sauer	B05	fertig
Heiss	B06	fertig
Hitschler	B10	fertig
Tomasetti	B14	fertig
Weber	B16	fertig
Matheis	B17	fertig
Wipfler	B18	fertig
Rösch	B20	fertig
Witmaier	B23	fertig
Knauber	B24	fertig
Gehr	B25	fertig
Bitter	B28	fertig
Otterbacher	B30	fertig
Faller,Rosa	B31	fertig
Zimmermann,	B33	fertig
Sommer, Fried	B35	fertig
Kerber	B38	fertig
Scholl, Emma		
Kassebart,Lotte		
Stein, Rudolf		
Weisbrod	B04	(fertig)
Ostwald	B09	(fertig)
Heger	B13	(fertig)
Haas	B19	(fertig)
Daum	B22	(fertig)
Götzmann	B29	(fertig)
Sessler	B32	(fertig)
Bickel	B34	(fertig)
Mock	B36 / S20	(fertig)
Krahforst	B37	(fertig)
Ricker	B26	fertigdropout
Buchauer	B40	

	Syrer		
	Wagenhals		
	Uhrich		
	Rinder		
	Schöneich		
	Lunzer		
	Schorr		dropout
	Endler		
	Volkwein		
	Klavinskis		
	Zimmermann, Luise		
	Schmid, Liesel		
	zahn		
	Manger		
	weiss, hannelore		
	kirchner,heimo		
	wauer		
	kirsch, berta		
	bernschneider, hertha		
	niedermeier,august		
	unseld,otto		
	wolf,waltraud		
	funk,frieda		
	Müller,Gustav Walter		
	Singer,Johann		
	Pfeiffer,Ernst-Günter		
	Becht,Norbert		
	Schwab, Bernhard		
	Lang, Herbert		
	Fleischer,Susanne		
	Busch,Herta		
0,97	Schmidt,Karl Hermann		
	Walther,Eva-Isolde		
	Barth, Dr Eva-Maria		
	Specht, Elisabeth		
	Beck,Irene		
	Heinrich,Lotte		
	Abraham, Rose		
	Wittmer,Georg		
	Frank,Helga		
	Fischer,Rosemarie		
	Loew, Hans		
	Schweizer,Dieter		
	Gayer,Harald		
	Ullrich, Ingrid		
	Schubert	B07	dropout
	Bauer	B08	dropout
	Schmitt,W	B11	dropout
	Brandel	B15	dropout
	Füsers	B21	dropout

Beck B

Grund dropout	dropSitzungzahl	dropVerlauf	Gesamtzahl Sitzungen	Entlasstag	Aufenthaltsdauer	Geschlecht
			16	29.01.2010	19	0
			14	25.01.2010	23	1
			15	27.01.2010	23	0
			16	22.01.2010	16	1
			14	18.02.2010	24	1
			17	15.03.2010	57	1
			12	17.02.2010	10	0
			13	05.03.2010	5	1
			17	19.03.2010	11	1
			16	20.04.2010	35	1
			16	05.07.2010	36	1
			17	24.05.2010	22	1
			16	25.05.2010	15	1
			16	24.06.2010	21	1
			15	09.07.2010	44	1
			16	07.07.2010	17	1
			15	15.07.2010	9	1
			11	29.09.2010	62	1
			15	04.08.2010	35	1
			15	20.08.2010	21	1
			6	01.09.2010	16	1
			17	15.09.2010	22	1
			17	03.09.2010	23	0
			17	03.11.2010	15	0
			17	09.12.2010	17	1
			17	13.12.2010	15	1
			2	14.01.2011	32	1
			15	18.01.2011	47	1
			15	01.02.2011	36	0
			17	03.02.2011	21	0
			17	26.01.2011	15	1
			17	23.03.2011	49	0
			13	23.03.2011	6	1
			15	16.02.2011		1
			15	20.05.2011	32	1
			16	09.06.2011	41	1
			8	21.06.2011	30	1
			15	20.06.2011	22	1
			11	28.06.2011	36	1
			17	07.06.2011	8	1
			15	27.06.2011	13	0
			16	18.07.2011	14	1
			15	13.07.2011	12	1
			10	26.07.2011	7	1
			13	23.09.2011	23	1
Ther beendet		gestorben nac	10	01.03.2010	53	1

Ther beendet		gestorben vor	16	7.4.	20	0
			10	08.02.2011	41	1
Partnerschaft	4	abgeschlosser	5	31.03.2010	5	1
Transport	1	abgeschlosser	3	10.06.2010	45	1
Transport	7		7	21.08.2010	32	1
zu anstrengen	6		7	27.05.2011	18	1
möchte nicht ı	2		3	09.06.2011	10	0
Antrieb	7		7	14.05.2011	27	1
AZ	3	gestorben nac	3	14.6.	25	0
gestorben vor	3	x	6	.08.06.2011	45	1
ı			16	08.02.2010	29	1
ı			17	19.03.2010	10	1
ı			16	13.04.2010	21	1
ı			17	21.04.2010	23	1
ı			15	16.03.2010	31	1
ı			10	11.08.2010	37	1
ı			18	17.09.2010	61	1
ı			15	02.09.2010	34	0
ı			16	05.07.2010	36	1
ı			16	30.09.2010	30	0
ı			16	30.09.2010	15	0
Schwäche	3		6	15.11.2010	22	1
			17	24.11.2010	28	1
			14	07.12.2010	20	1
			7	15.12.2010	22	1
ı			10	02.02.2010	36	1
			14	12.01.2011	34	1
				31.01.2011	21	1
ı			15	31.01.2011	20	1
			11	08.03.2011	15	1
			6	07.03.2011	34	0
			15	01.04.2011	33	0
			16	31.03.2011	10	1
			7	18.04.2011	61	0
				13.05.2011	29	1
				16.05.2011	54	1
			10	03.06.2011	24	1
			12	24.06.2011	45	0
zu anstrengen	4		6	04.10.2010	33	1
				25.03.2011	38	1
				21.03.2011	25	1
Ther beendet	15	gestorben vor	16	08.03.2010	28	1
keine Gruppe	0	abgeschlosser	0	25.01.2010	4	1
keine Gruppe	0	abgeschlosser	3	12.02.2010	12	1
kranke Leute i	5	abgeschlosser	5	12.03.2010	24	1
Tk zu anstreng	1	abgeschlosser	1	12.03.2010	33	1
Antrieb	0	abgeschlosser	0	23.03.2010	8	1
Transport	1	abgeschlosser	2	22.04.2010	22	1
möchte nur zu	0	abgeschlosser	2	28.05.2010	74	1
fühlt sich gut	1	abgeschlosser	1	21.06.2010	7	1

keine Gruppe	0		1	22.06.2010	23	0
möchte keine	0		2	05.07.2011	22	1
AZ, veriwrrt	1		1	12.06.2010	17	1
möchte nur st	1		2	02.08.2010	36	1
möchte neu b	0		0	06.07.2010	35	1
geht besser	0		0	23.08.2010	25	1
Antrieb	2	abgeschlosser	2	11.08.2010	38	1
Antrieb	6	abgeschlosser	7	08.09.2010	38	1
Antrieb, Fußl	2		2	23.07.2011	39	1
Kognition, zu v	1	abgeschlosser	1	24.08.2010	58	1
Familie dageg	2		2	05.10.2010	15	1
nicht nötig	1		1	08.10.2010	5	1
besucht Tk im	2		2	13.11.2010	32	1
Antrieb	1		1	02.11.2010	30	0
Antrieb, habe	0	abgeschlosser	0	08.07.2010	22	1
	1		0	13.11.2010	19	1
Antrieb	4		6	19.11.2010	12	1
Erschöpfung	0		1	09.12.2010	21	1
möchte nicht	0		0	02.08.2010	21	1
möchte nicht	0		1	16.12.2010	33	1
AZ	1		2	06.09.2010	28	1
nichts mehr zu	1		1	17.01.2011	22	0
keine Zeit	0		1	21.01.2011	15	1
Morgentief	1		1	26.01.2011	37	1
soz Rückzug	1		1	25.01.2011	35	1
fühlt sich bess	2		2	10.03.2011	15	1
möchte krank	3		3	14.03.2011	54	1
Belastbarkeit,	1		1	29.03.2011	14	1
antrieb abbau			1	06.04.2011	21	0
zu anstrengen	2		2	22.02.2011	33	1
geht besser	0		0	21.04.2011	49	1
AZ			2	29.04.2011	31	0
möchte nur st	2		2	13.05.2011	30	1
			4	27.05.2011	11	1
Antrieb	1		1	30.05.2011	16	1
inkontinenz			2	03.06.2011	37	1
			1	11.06.2011	34	1
apoplex,hypak	3		7	01.07.2011	5	0
hypakusis, angebote im heim gut			6	01.07.2011	52	1
				14.06.2011	64	0
Antrieb	2		2	06.10.2010	56	1
				18.01.2011	21	1
Transport	0	gestorben vor	0	20.01.2010	16	0
AZ, Antrieb	0	gestorben vor	2	04.02.2010	24	1
keine Hoffnun	0	gestorben vor	6	04.03.2010	49	1
Transport	0	abgeschlosser	5	17.04.2010	47	1
gestorben nac	0	x	0	07.01.2011	29	1
AZ	4	gestorben vor	7	07.12.2010	10	0
gestorben vor	0	x	0	22.01.2010	11	1

gestorben vor	0 x	2	02.03.2010	22	1
gestorben vor	0 x	0	06.05.2010	30	0
gestorben nac	0 x	7	20.06.2010	55	0
gestorebn vor	1 x	1	20. Okt .15		0
gestorben vor	1 x	1	11.02.2011	22	1
gestorben vor	2 x	2	02.03.2011	30	1
gestorben vor	1 x	1	01.03.2011	16	1
gestorben vor	1 x	1	06.05.2011	29	1
gestorben		3	24.06.2011	22	1

		25.1.	1
		19.2.	0
		22.2.	1
		17.3.	0
		6.4.	1
		9.4.	1
		25.3.	0
		14.4.	1
		26.4.	1
		7.5.	1
		14.5.	1
		14.5.	1
		7.6.	1
		11.6.	1
		21.5.	1
		29.6.	1
		5.8.	1
		10.8.	1

		15.2.	0
		16.3.	1
		26.3.	1
		25.4.	0
		28.4.	1
		27.5.	1
		8.6.	0
		2.7.	1
AZ	1	9.6.	1
		28.7.	1
gestorben		8.7.	0
		24.8.	1

		10.9.		1
		16.9.		1
		22.10.		1
		30.9.		1
		6.10.		1
		8.10.		1
gestorben	0	18.10.		1
		24.10.		1
		25.10.		1
		2.12.		0
		9.12.		1
		09.02.2011	15	0
gestorben	0	5.3.		0
gestorben	0	9.3.		0
gestorben	0	22.3.		0
gestorben	0	7.4.		1
gestorben	0	27.4.		0

fühlt sich zu al	2	8.11.	1

Jahrgang	HADS 3	HADS3-Schweregrad	HADS3-Depr	HADS3-Deprscore	HADS3-Angst	HADS3-Angststrg
1925	12	2	8	1	4	1
1931	14	2	6	1	8	1
1934	4	0	3	0	1	0
1922	17	3	9	1	8	1
1939	10	1	9	1	1	0
1934	16	3	8	1	8	1
1927	14	2	10	1	4	1
1931	19	3	12	1	7	1
1924	7	0	5	1	2	0
1932	4	0	2	0	2	0
1920	4	0	2	0	2	0
1939	31	4	20	1	11	1
1929	18	3	7	1	11	1
1930	12	2	10	1	2	0
1927	25	4	14	1	11	1
1926	19	3	14	1	5	1
1938	7	0	2	0	5	1
1943	3	0	3	0	0	0
1929	9	1	4	1	5	1
1925	26	4	15	1	11	1
1924	1	0	1	0	0	0
1932	5	0	2	0	3	0
1926	5	0	3	0	2	0
1922	4	0	4	1	0	0
1941	9	1	6	1	3	0
1929	4	0	1	0	3	0
1936	5	0	1	0	4	1
1924	3	0	3	0	0	0
1924	26	4	12	1	14	1
1917	10	1	10	1	0	0
1939				0		0
1928	12	2	8	1	4	1
1932	9	1	6	1	3	0
1928	17	3	8	1	9	1
1928	14	2	10	1	4	1
1932	5	0	3	0	2	0
1916	19	3	14	1	5	1
1930	19	3	10	1	9	1
1928	7	0	3	0	4	1
1929	30	4	16	1	14	1
1931	8	1	7	1	1	0
1936	2	0	1	0	1	0
1939	19	3	12	1	7	1
1929	10	1	8	1	2	0
1924	11	2	6	1	5	1
1925				0		0

1931				0		0
1927				0		0
1928	26	4	12	1	14	1
1928	18	3	13	1	5	1
1926	38	4	21	1	17	1
1935	14	2	3	0	9	1
1917	25	4	16	1	9	1
1927	29	4	17	1	12	1
1926				0		0
1919				0		0
1928	17	3	7	1	10	1
1928	17	3	10	1	7	1
1925	7	0	5	1	2	0
1928	22	4	14	1	8	1
1934	17	3	6	1	11	1
1926	30	4	14	1	16	1
1928	14	2	7	1	7	1
1934	4	0	1	0	3	0
1924	7	0	6	1	1	0
1926	16	3	7	1	9	1
1932	21	3	8	1	13	1
1936	17	3	9	1	8	1
1925	27	4	14	1	13	1
1927	15	3	8	1	7	1
1933				0		0
1932	15	3	6	1	9	1
1933	12	2	6	1	6	1
1925	2	0	2	0	0	0
1931	30	4	15	1	15	1
1922	8	1	5	1	3	0
1933	19	3	14	1	5	1
1920	4	0	0	0	4	1
1929	23	4	14	1	9	1
1928	7	0	7	1	0	0
1927	6	0	3	0	3	0
1933	14	2	13	1	1	0
1932	17	3	14	1	3	0
1942	21	4	11	1	10	1
1931	30	4	14	1	16	1
1925	32	4	20	1	12	1
1938	8	1	5	1	3	0
1926				0		0
1918	18	3	6	1	12	1
1922	26	4	19	1	7	1
1931	36	4	20	1	16	1
1920	34	4	21	1	13	1
1939	14	2	8	1	6	1
1925	18	3	10	1	8	1
1925	18	3	11	1	7	1
1935	20	3	16	1	4	1

1924	24	4	18	1	6	1
1931	18	3	9	1	9	1
1920	33	4	20	1	13	1
1922	24	4	19	1	5	1
1928	14	2	10	1	4	1
1929	11	2	7	1	4	1
1925	33	4	20	1	13	1
1930	37	4	21	1	16	1
1923	21	3	13	1	8	1
1928	15	3	12	1	3	0
1925	11	2	7	1	4	1
1945	8	1	1	0	7	1
1936	8	1	4	1	4	1
1931	28	4	17	1	11	1
1928	17	3	14	1	3	0
1926	22	4	15	1	7	1
1928	35	4	20	1	15	1
1940	25	4	11	1	14	1
1922	18	3	14	1	4	1
1922	12	2	8	1	4	1
1926	26	4	16	1	10	1
1926	14	2	12	1	2	0
1934	14	2	6	1	8	1
1925	19	3	14	1	5	1
1924	17	3	13	1	4	1
1928	12	2	4	1	8	1
1925	37	4	20	1	17	1
1942	16	3	10	1	6	1
1922	37	4	21	1	16	1
1930	20	3	13	1	7	1
1921	18	3	13	1	5	1
1930	17	3	10	1	7	1
1920	24	4	18	1	6	1
1936	21	3	12	1	9	1
1928	15	3	9	1	6	1
1928	9	1	5	1	4	1
1936	21	3	10	1	11	1
1929	25	4	15	1	10	1
1922	22	4	13	1	9	1
1948				0		0
1930				0		0
1931				0		0
1923				0		0
1927				0		0
1930				0		0
1938				0		0
1921				0		0
1933				0		0
1930				0		0

1923		0	0
1933		0	0
1922		0	0
1931		0	0
1920		0	0
1940		0	0
1928		0	0
1929		0	0
1931		0	0

1924		0	0
1925		0	0
1923		0	0
1923		0	0
1928		0	0
1929		0	0
1926		0	0
1930		0	0
1926		0	0
1922		0	0
1943		0	0
1923		0	0
1923		0	0
1934	19	0	0
1929	22	0	0
1927	14	0	0
1931	22	0	0
1922	18	0	0
		0	0
		0	0
		0	0
1920	31	0	0
1925	19	0	0
1928	24	0	0
1934	33	0	0
1926	23	0	0
1929	8	0	0
1923	38	0	0
1928	15	0	0
1925	37	0	0
1924	14	0	0
1921	39	0	0
1932		0	0

1926				0		0
1921				0		0
1929				0		0
1938				0		0
1921				0		0
1935				0		0
1917				0		0
1932				0		0
1936				0		0
1921				0		0
1935				0		0
				0		0
				0		0
				0		0
				0		0
				0		0
				0		0
				0		0
				0		0
				0		0
				0		0
				0		0
				0		0
				0		0
				0		0
				0		0
				0		0
				0		0
				0		0
				0		0
				0		0
1930		27	17	1	10	1
				0		0
				0		0
				0		0
				0		0
				0		0
				0		0
				0		0
				0		0
				0		0
				0		0
				0		0
				0		0
				0		0
1934	x			0		0
1928	x			0		0
1931	x			0		0
1925	x			0		0
1928	x			0		0

1921	0	0

HAMD 3-gesamt	HAMD3-Schweregrad	Responder	Remitter	MMSE 3	WohnAnders?	Wohnsituation3
10	1	1	0	26	0	1
10	1	1	0	30	0	1
3	0	1	1	26	0	1
12	2	0	0	30	0	1
4	0	1	0	30	0	4
7	0	0	0	30	0	2
9	1	0	0	25	0	2
10	1	0	0	30	0	1
5	0	1	1	29	0	4
11	2	1	1	30	0	2
5	0	1	1	30	0	2
23	3	0	0		0	2
12	2	0	0	29	0	2
5	0	0	0	23	0	1
27	4	1	0		0	1
17	3	0	0	17	0	2
11	2	1	1	30	0	2
4	0	1	1	24	0	1
7	0	1	0	30	0	1
26	4	0	0		0	1
3		1	1	30	0	2
4	0	1	1	30	0	1
8	1	1	1	30	0	1
8	1	1	1	30	0	1
12	2	0	0	30	0	2
1	0	1	1	29	0	1
4		1	1	29	0	4
7		1	1	24	0	1
15	3	0	0	30	0	1
7		1	0	21	1	4
		1	1		0	2
4		0	0	30	0	1
9	1	1	0	26	0	5
9	1	1	0	30	0	1
6	0	0	0	30	0	1
3	0	1	1	29	0	2
14	2		0	29	0	1
12	2		0	27	0	1
5			1	29	0	2
24	3		0	29	0	1
5			0	30	0	1
1	0	1	1	30	0	1
16	3	0	0	26	0	1
12	2		0	26	0	1
10	1		0	30	0	2
		1	1			

			1			
		1	1			
28	4	0	0		0	2
10	1	0	0	20	0	2
30	4		0	20	0	4
14	2		0	28	0	2
17	3	0	0	27	0	1
25	4	0	0	14	1	4
			1			
		1	1			
17	3	0	0	28	1	4
21	3	1	0	28	0	1
5	0	1	1	30	0	1
15	3	0	0	30	0	1
11	2	1	0	30	0	1
30	4	0	0		0	1
14	2	1	0	30	0	1
2	0	1	1	30	0	1
5	0	1	1	29	1	4
10	1	0	0	29	0	1
13	2	1	0	20	0	1
10	1	1	0	30	0	2
23	3	0	0	29	0	1
15	3		0	27	0	1
			1		0	1
13	2	0	0	28	0	1
11	2		0	25	1	2
2	0	1	1	25	0	2
23	3		0	23	0	1
10	1		0	25	0	4
11	2		0	25	0	1
3	0	1	1	29	0	1
18	3	0	0	21	0	1
2	0	1	1	30	0	4
3			1	25	0	4
8	1	1	0	27	0	1
12	2	1	0	29	1	4
17	3	0	0	30	0	4
17	3		0	29	0	4
18	3		0	30	0	1
14	2	1	0		0	2
		1	1			
17	3	0	0	19	0	1
23	3	0	0	10	1	1
38	4	0	0	27	0	1
32	4	0	0		0	1
18	3	0	0	21	0	4
26	4	0	0	24	0	1
25	4	0	0	24	0	1
20	3	0	0	30	0	2

27	4	0	0		0	2
10	1	0	0	30	0	1
31	4	0	0	15	1	4
21	3	0	0	27	0	4
12	2	1	0	26	0	2
18	3	1	0	29	1	1
31	4	0	0	27	0	1
37	4	0	0	23	0	1
18	3	0	0	28	0	1
15	3	0	0	12	0	4
17	3	0	0	28	1	4
13	2	1	0	30	0	1
8	1	0	0	26	0	4
25	4	0	0	22	0	1
15	3	0	0	21	0	1
19	3	0	0	25	0	4
35	4	0	0	23	0	2
23	3	0	0	27	0	1
21	3	0	0	23	0	2
12	2	0	0		1	4
31	4	1	0	23	1	4
12	2	0	0	30	0	4
15	3	1	0	29	0	1
18	3	0	0	19	0	4
19	3	1	0	25	0	4
14	2	1	0	25	0	1
31	4	0	0	23	0	4
21	3	0	0	23	0	4
34	4	0	0	27	0	1
24	3	1	0	24	0	1
16	3	1	0	24	0	4
16	3	0	0	22	0	2
27	4	0	0	27	0	2
23	3	0	0	18	0	1
13	2	0	0	24	0	1
14	2	0	0	23	0	4
18	3	0	0	27	0	4
24	3	0	0	26	0	1
22	3	0	0	27	0	4
			1			
		1	1			
			1			
		1	1			
		1	1			
		1	1			
		1	1			
		1	1			
		1	1			
		1	1			

	1	1		
	1	1		
	1	1		
	1	1		
	1	1		
	1	1		
	1	1		
	1	1		
	0	1		

	0	1		
	0	1		
	0	1		
	0	1		
	0	1		
	0	1		
	0	1		
	0	1		
	0	1		
	0	1		
	0	1		
	0	1		
	0	1		
26	0	0	x	
18	0	0		29
13	0	0		28
27	0	0		24
23	0	0		27
	0	1		
	0	1		
	0	1		
24	0	0	x	
20	0	0	x	
19	0	0	x	
25	0	0		11
18	0	0	x	
18	0	0	x	
40	0	0		
14	0	0	x	
33	0	0	x	
10	0	0	x	
36	0	0		0
	0	1		

			0	1				
			0	1				
			0	1				
			0	1				
			0	1				
			0	1				
			0	1				
			0	1				
			0	1				
			0	1				
			0	1				
			0	1				
			0	1				
			0	1				
			0	1				
			0	1				
			0	1				
			0	1				
			0	1				
			0	1				
			0	1				
			0	1				
			0	1				
			0	1				
			0	1				
			0	1				
			0	1				
			0	1				
			0	1				
			0	1				
			0	1				
	27	4	0	0		25	0	2
			0	1				
			0	1				
			0	1				
			0	1				
			0	1				
			0	1				
			0	1				
			0	1				
			0	1				
			0	1				
			0	1				
			0	1				
			0	1				
x				0	x			
x				0	x			
x				0	x			
x				0	x			
x				0	x			

0 1

Zusammenleben3	Alleinlebend?3	SoS-gesamt3	Sos-Kontakte3	SoS-Aktivitäten3	SoS-Wohn3	SoS-Geld3
2	0	23	6	4	11	2
2	0	20	5	4	9	2
2	0	18	6	3	7	2
1	1	17	2	2	10	3
1	0	16	5	2	7	2
1	1	19	6	3	10	0
2	0	18	4	1	10	3
1	1	23	6	5	9	3
6	0	16	6	1	9	0
1	1	23	6	4	10	3
1	1	22	6	3	10	3
2	0	17	6	0	9	2
1	1	21	5	4	11	1
1	1	19	6	2	9	2
1	1	17	6	1	8	2
2	0	16	6	0	9	1
1	1	22	6	4	10	2
2	0	18	6	4	7	1
2	0	18	5	3	7	3
2	0	17	6	0	9	2
1	1	22	6	4	10	2
1	1	25	6	5	11	3
1	1	20	5	3	9	3
3	0	22	6	4	9	3
1	1	17	5	3	8	1
1	1	22	6	4	10	2
6	0	21	6	5	7	3
1	1	14	2	1	8	3
1	1	18	4	4	8	2
1	0	16	5	1	9	1
1	1	20	5	5	9	1
1	1	22	6	3	11	2
1	1	22	5	4	10	3
2	0	18	5	2	9	2
1	1	22	6	4	10	2
1	1	21	6	4	9	2
1	1	21	6	3	10	2
1	1	17	5	2	8	2
3	0	20	6	3	9	2
1	1	23	6	5	9	3
2	0	20	6	3	8	3
1	1	22	4	5	10	3
1	1	16	6	4	5	1
1	1	22	6	3	10	3
1	1	22	6	4	10	2

2	0	16	5	0	10	1
1	1	16	4	2	9	1
1	1	11	2	0	7	2
1	1	19	5	2	10	2
1	1	14	1	1	9	3
6	0	13	2	0	9	2

5	0	16	4	2	9	1
2	0	17	4	2	9	2
3	0	21	6	3	9	3
1	1	19	4	3	9	3
1	1	20	4	4	9	3
2	0	16	2	2	9	3
2	0	23	5	4	11	3
1	1	21	5	5	8	3
1	0	19	5	2	10	2
1	1	17	4	2	9	2
1	1	15	1	3	8	3
1	1	23	6	4	10	3
1	1	17	4	1	9	3
1	1	20	6	2	9	3
2	0	22	6	4	10	2
3	0	17	5	1	9	2
3	0	18	6	1	10	1
4	0	23	6	5	10	2
1	1	15	2	3	7	3
6	0	14	2	4	8	0
2	0	18	6	4	6	2
2	0	22	6	4	9	2
2	0	18	6	0	10	2
2	0	16	3	2	8	3
6	0	21	5	4	10	2
2	0	18	4	2	9	3
6	1	21	6	3	10	2
6	1	15	5	1	8	1
6	1	18	5	3	8	2
1	1	12	4	1	4	3
1	1	18	5	3	9	1

1	1	18	3	3	9	3
7	0	17	5	0	10	2
2	0	18	5	0	10	3
1	1	13	3	0	8	2
1	0	19	5	3	10	1
3	0	16	6	3	5	2
2	0	19	6	1	9	3
1	1	17	6	1	9	1

1	1	15	1	1	10	3
1	1	14	2	1	8	3
6	1	13	3	1	8	1
6	0	14	5	0	7	2
1	1	18	3	2	10	3
2	0	18	6	0	10	2
1	1	11	4	0	7	0
1	1	17	6	1	8	2
3	0	17	6	1	8	2
6	1	15	6	0	9	0
6	0	16	6	2	8	0
2	0	19	4	4	8	3
1	1	20	6	4	10	0
2	0	17	6	0	9	2
2	0	18	5	2	9	2
1	1	17	4	1	10	2

1	1	11	2	0	8	1
2	0	15	5	0	8	2
1	1	19	6	1	10	2
1	0	18	6	2	8	2
6	0		4	1	9	
1	1	19	5	3	9	2
7	1	18	4	4	8	2
6	1	15	5	2	8	0
6	0	13	1	1	10	1
3	0	19	5	3	9	2
		15	3	0	10	2
6	0	16	4	3	7	2
2	0	18	5	0	10	3
1	1	19	3	2	11	3
6	0	15	4	1	8	2
2	0	18	6	0	10	2
1	1	17	5	0	9	3
2	0	17	3	2	10	2
1	1	18	6	3	7	2
6	0	20	6	4	8	2
1	1	14	2	1	8	3
1	1	16	3	1	9	3
6	0	16	4	1	10	1

1	1	2	1	10

Frequency going out3	SF-12-3-gesamt	SF-12-3-phys	SF12-3-psych	Ressource util3-Gesamtscore	Ressource util3- Pat stationär	Ressource util3- Anzahl stationär
	34	15	19		0	0
	30	13	17		1	9
	42	17	25		0	0
	23	9	14		1	4,38
	29	10	19		1	4,63
	24	8	16		0	0
	33	13	20		0	0
	24	10	14		0	0
	34	8	26		1	4,13
	38	12	26		1	3,88
	44	16	28		1	1
	23	10	13		1	12,5
	30	12	18		0	0
	33	11	22		0	0
	24	11	13		0	0
	21	7	14		1	3,38
	27	9	18		1	4,25
	33	8	25		0	0
	30	10	20		0	0
	20	6	14		1	1,5
	37	12	25		1	1,5
	40	17	23		0	0
	40	15	25		1	1,13
	41	17	24		1	1,38
	33	14	19		0	0
	46	18	28		1	3,63
	34	11	23		1	0,88
	28	11	17		0	0
	29	8	21		0	0
	26	11	15		0	0
	28	14	14		0	0
	43	17	26		1	0,5
	28	9	19		0	0
	22	9	13		1	4,88
	21	7	14		0	0
	33	10	23		0	0
	28	12	16		0	0
	30	12	18		0	0
	34	8	26		0	0
	19	8	11		0	0
	34	12	22		0	0
	41	15	26		0	0
	23	8	15		0	0
	27	10	17		0	0
	25	8	17		0	0

19	8	11	0	0
32	12	20	1	1,88
14	6	8	0	0
27	9	18	0	0
17	6	11	1	2,75
20	6	14	0	0
23	8	15	1	8,75
28	11	17	1	3,5
34	12	22	0	0
26	7	19	0	0
31	13	18	0	0
16	6	10	0	0
34	13	21	0	0
45	18	27	0	0
33	12	21	1	3,88
30	8	22	0	0
41	18	23	0	0
34	13	21	1	5
22	10	12	1	1,88
26	9	17	0	0
24	11	13	1	5,63
31	13	18	1	5
26	9	17	0	0
34	10	24	0	0
19	8	11	0	0
37	15	22	0	0
25	7	18	1	0,25
43	16	27	0	0
23	9	14	0	0
28	10	18	1	4,13
26	9	17	0	0
26	12	14	1	2,63
22	8	14	1	3,38
22	8	14	1	5,5
19	8	11	1	2,38
20	9	11	1	2,38
34	11	23	1	3,625
27	11	16	0	0
20	8	12	0	0
14	6	8	0	0
15	8	7	0	0
25	7	18	1	0,38
23	8	15	1	2,25
25	12	13	0	0
20	7	13	1	1,88

21	11	10	0	0
23	8	15	0	0
18	8	10	0	0
24	11	13	0	0
23	10	13	1	4,88
28	10	18	0	0
17	6	11	0	0
14	6	8	0	0
22	7	15	0	0
33	12	21	0	0
27	9	18	1	1,25
30	12	18	1	0,25
37	14	23	0	0
19	9	10	1	5,88
26	11	15	0	0
21	7	14	1	
12	6	6	1	2,63
20	7	13	1	2,63
25	9	16	0	0
27	8	19	0	0
19	8	11	0	0
32	12	20	1	
25	8	17	0	0
23	10	13	0	0
24	11	13	0	0
28	9	19	0	0
20	7	13	0	0
27	10	17	0	0
14	6	8	0	0
15	6	9	0	0
17	7	10	0	0
26	11	15	1	4,13
16	6	10	0	0
29	14	15	0	0
25	8	17	0	0
32	11	21	0	0
26	11	15	1	1,13
16	6	10	1	5,63
16	6	10	1	0,75

8	9	0	0

Ressource util3- Diagnose	Ressource util3- Abteilung	Ressource util3- Notaufnahme	Ressource util3- Anzahl Notaufnahme	Ressource util3- Behandlung Heilberuf	Ressource util3- Art Behandlung	Ressource util3- Pflegedienst
		1	1	1	1,3,5,10	1
Kyphoplastie b	1,3,	0	0	1	1,5,10	1
		0	0	1	1,3,8	1
kardiopulmon	3	1	0,5	1	1,5,8,10	1
starke Zunahn	5	1	0,5	1	1,5,10	1
		0	0	1	1,5,10	1
		0	0	1	1,10,	0
		0	0	1	1,10,	0
progrediente I	1	1	0,5			1
Entfernung de	4,5,	0	0	1	1,3,5,9,10	1
sonstige Vergi	3	1	1	1	1,5,9,10,11,12	0
Parkinson-Syn	1,3,9	0	0	1	1	0
		0	0	1	1,5,9	1
		1	0,5	1	1,3,5,10	1
		1	0,5	1	1,5,10	1
kardiale Dekoı	3	0	0	1	1,10,	1
paroxysmale A	2,3,	1	0,5	1	1,3,5,9,10	1
		0	0	1	1,5,9,10	0
		0	0	1	1,9,10,12	1
Lumboischialg	5	1	1	1	1,5	1
Beginnende ka	3	0	0	1	1	0
		0	0	1	1,5,9,10	0
instabile Angir	3	1	0,5	1	1,5,10	1
Lichenoides El	9	0	0	1	10,1	0
		0	0	1	1,3	1
Reha nach Rel	3	0	0	1	1,9,10	0
Z.n. Knie-TEPr	5	0	0	1	1,4,5,7	1
0	0	0	0	1	1	1
		0	0	1	1,5,10	0
	1	0	0	1	1	1
		0	0	11	1,5,8	0
MI	3	0	0	1	1	0
		0	0	1	1,5,12	1
Stolpersturz m	1,5,	0		1	1,4,	1
		0	0	1	1,5,9,10,11	1
		0	0	1	1,3,	1
		0	0	1	1,11	1
		0	0	1	1,5,12	1
		0	0	1	1	1
		0	0	1	1,12	0
		0	0	1	10,1	1
		0	0	1	1,3,	1
		0	0	1	1	1
		0	0	1	1,4,6,9	1
		0	0	1	1,5	1

		0	0	0		0
kardiale Dekoı	3	0	0	1	1,5,6	1
		0	0	1	1	1
0	0	1	0,5	1	1	1
Schenkelhalsfı	1	1	0,5	1	1,5,	1
		0	0	1	1	1
Angina pector	1,3,4	1	0,5	1	1,5	1
Unterbauchbe	1	1	4	1	1,8,10	0
		0	0	1	1,5,8,10	1
		0	0	1	1,8,10	1
		0	0	1	1,5,8,10	0
		1	1	1	1,5,9,10,12	1
		0	0	1	1,8	1
		0	0	1	1,5,8,10	1
instabile Angiı	1	0	0	1	1,5,10	1
		1	1	1	1,8,10,12	0
		0	0	1	1,8,12	0
Apoplex, MI	3,6,	1	0,5	1	1,11,10	0
Reha zur Verb	1,4,	0	0	1	1,5,12	1
		0	0	1	10,1	1
Pneumonie re	1,6,2,4	0	0	1	1,4,5,8,10	0
kardiale Dekoı	1,3,	0	0	1	1,5,10	1
		0	0	1	1,5	0
		1	0,5	1	1,8,	1
		0	0	1	1,5,8	1
		0	0	1	1,8,	1
Gastritis	7	0	0	1	1,5,12	0
		0	0	1	1,3,8,10,12	1
		1	0,5	1	1,5,8,12	1
Z.n. pertrocha	1,5,	1	1	1	1,5,10,12	1
		0	0	1	1,5,8	1
Medulla-obloı	6	0	0	1	1,5,	1
Z.n. Sturz mit	1	0	0	1	1,12,	1
acute on chroı	9	0	0	1	1,5,	1
Spondylitis DD	7	1	1	1	1,5,10	1
LA Cataracta c	3,9,	0	0	1	1,12	0
Wundheilungs	4	0	0	1	1,5,8,10	1
		0	0	1	1	1
		0	0	1	1,5	1
		0	0	1	1,4,5,10	1
		1	0,5	1	1	1
traumatische	5	1	0,5	1	1,5,10	1
Anämie unter	3	1	1,5	1	1,5,10	1
		1	0,5	1	1,5,10	1
Knie-TEP re be	4	0	0	1	1,5,9,10	1

			0	0	1	1	0
			0	0	1	1	1
			0	0	1	1	1
			0	0	1	1,5	1
1-Kammer-Sch	3,7,		0	0	1	1,9	1
0			1	0,5	1	1,5,12	1
			0	0	1	1,5,10	0
			0	0	1	1,?	1
			0	0	1	1,5	1
			0	0	1	1	1
Multifaktorell		8	1	0,5	1	1,5,10	1
Lokalreaktion		9	1	1x	1	1,1	0
			0	0	1	1	1
Atherosklerot		3	1	0,5	1	1,5	1
			0	0	1	1,5,10	1
"Depression"		2	1		1	1,4,10	1
Desmopressin		1	1	2x	1	1	1
			0	0	1	1,5,9,10,11	1
					1	1	1
			0	0	1	1,5	1
			0	0	1	1,5	1
s. Akte	2,3,		0	0	1	1	1
			0	0	1	1,4,8	1
			0	0	1	1	1
			0	0	1	1,5,9	1
			1	2x	1	1,3,4,5,9,10	1
			0	0	1	1	1
			0	0	1	1,3,5,6,11	1
			0	0	1	1	1
			0	0	1	1,5,9,12	1
			1	0,5	1	1,5	1
Urothel-Ca pT		9	0	0	1	1,3,5,10	1
			0	0	1	1,9	1
			0	0	1	1,6,5	1
			0	0	1	1,4	1
			0	0	1	1,5,9	1
V.a. symptom	7,8,		1	1	1	10,1	1
Apoplex	3,6,		1	0,5	1	1,3,11	1
akutes Nieren		9	0	0	1	1,4,5,9	1

0	0	0	0	1	1,5,10	0

Ressource util3- Art Pflegedienst	HRA-O-3	Körperlich Aktiv3	Aktivität3	Art der Aktivität3	Weniger aktiv3	Gerne mehr3
1,2		1	2	1,3	0	1
1,2		1	5,5	2,3	0	1
1		1	42	1,3	0	1
1,2		1	35	1,2	0	1
6		1	1	2	0	1
1,2,5		1	25	1,2,3	0	1
		1	14	1,3	0	1
		1	28	1,3	0	1
6		1	21	1,3	0	1
2		1	21	1,2,3	0	1
		1	56	1,2,3	1	0
		0	0		0	1
1		1	35	1,3	0	1
6		0	3	2	0	1
7		1	1	2,3	0	1
1		0	0		0	1
1		1	49	1,3	1	0
		1	8	1,2,3	0	1
7		1	21	1,3	0	1
1,2		1	0,67	3	0	1
		1	21	1,2,3	0	0
		1	42	1,3	0	1
1,2,3		1	14	2,3	0	1
		1	14	1,3	0	0
1		1	42	1,3	0	1
		1	49	1,3	1	0
6		1	12,5	2,3	0	1
2		1	14	3	0	1
		1	5	2,3	0	1
6		1	2	2	0	1
		1	17,5	1	0	1
		1	70	1,3	0	1
1,2		1	35	1,3	0	1
1,3,		1	2,25	1	0	1
7		1	10,5	3	0	1
1,2,		1	35	1,3,	0	1
1,2		1	34,5	1,3	0	0
1		1	70	1,2	1	1
1		1	21	1,3	0	0
		0	0		0	1
2		1	14	1,2	0	1
1,2,		1	21	1,2,3	1	0
1		1	35	1	0	1
1		1	29	1,2,3	0	1
1,2		1	4	2,3	0	1

	0	0		0	1
1,7	0	0		0	1
6	0	0		0	1
1,2	1	21	1,3	0	1
2.3,	0	0		0	1
6	0	0		0	1
6	1	3	2,3	0	1
	1	7	1,3	0	1
1,2	1	15	1,2,3	0	0
1	1	14	1,3	0	1
	1	52	1,2,3	1	0
2	1	15	1,2	0	1
2	1	42	1,2,3	0	0
2	1	28	1,3	0	0
6	1	1,5	2	0	1
	1	14	1	0	1
	1	21	1,3	0	0
	1	21	1,3	0	1
7	1	14	3	0	1
7	1	7	2	0	1
	1	7	3	0	1
1,2	1	14	2,3	0	1
	1	5	1,2	0	1
1,2,	1	10,5	3	0	1
1,2	1	21	1	0	1
6	1	23	1,2,3	1	0
	1	5	2,3	0	0
2	1	28	3	1	0
3	1	3,5	3	0	1
6	1	3,5	2	0	1
6	1	28	1,2,3	0	1
2,8,	1	18	1,2,3	0	1
6	1	1	2	0	1
6	1	2	2	0	1
1,2,3	1	3,5	3	0	1
	0	0		0	1
1,2,	1	30	2,3,	0	0
1,2	1	49	1,2	0	1
7	0	0		0	1
1,2	0	0		0	1
6	0	0		0	1
1	1	4	1,3	0	1
1	0	0		0	1
7	0	0		0	1
1,2,3,5	0	0		0	1

		0	0		0	0
	1	0	0		0	1
	6	0	0		0	1
	6	0	0		0	0
	1,2	0	0		0	1
	1	0	0		0	1
		0	0		0	1
	7	0	0		0	1
	2	0	0		0	1
	6	0	0		0	0
	6	1	1	2	0	1
	0	0	0		0	1
	6	1	7	3	0	0
	1	0	0		0	1
	1	1	4,9	3	0	1
	6	1	3,5	3	0	1
	1,2	0	0		0	0
	2	0			0	1
	1	1	1,5	3	0	0
	6	0	0		0	1
	6	0	0		0	1
	6	1	7	2,3	0	1
	2,7	1	3,5	3	0	1
	6	1	3,5	3	0	1
	6	0	0		0	0
	2,3	1	2,5	3	0	1
	6	0	0		0	1
	6	0	0		0	1
	1,2	0	0		0	1
	2	1	1,5	2	0	1
	6	1	3	2	0	1
	1	0	0		0	1
1,2,3		0	0		0	1
1,2,4		0	0		0	0
	1,2	0	0		0	1
	6	1	7	1,3	0	1
	1,2	0	0		0	1
	7	0	0		0	1
	6	1	1	2,3	0	1

0	1	14	3	0	1

Wasser tags3	Wasser nachts3	RR Kontrolle3	RR hoch3	Gyn/Uro3	FettKontrolle 3	Fett hoch3
1	1	1	0	0	1	1
0	1	1	1	0	1	1
1	1	1	1	0	1	0
0	0	1	0	0	1	1
0	0	1	0	0	1	1
0	0	1	0	0	0	1
1	1	1	0	0	1	0
1	0	1	0	1	0	0
0	0		1			1
1	1	1	0	0	1	1
1	0	1	0	0	1	1
1	1	1	0	0	0	0
0	1	1	0	0	1	1
0	0	1	0		1	1
1	0	1	1	0	1	1
0	0	1	0	0	0	0
1	1	1	0	0	1	1
0	0	1	1	0	1	1
1	1	1	1	0	0	0
0	0	1	0	1	0	1
1	0	1	0	0	1	0
0	0	1	1	0	1	1
1	1	1	1	0	1	1
1	0	1	0	1	0	1
0	1	1	0	1	0	0
0	0	1	1	0	1	1
1	1	1	0	0	0	0
1	1	1	1	0	0	1
0	0	1	1	0	1	1
0	0	1	0	0	0	1
0	0	1	0	1	1	0
0	1	1	0	0	1	
1	0	1	0	0	1	1
1	1	1	0	0	1	0
0	0	1	0	0	1	1
0	0	1	0	0	1	1
1	1	1	0	0	0	0
0	0	1	1	1	1	1
1	1	1	0	0	1	0
0	0	1	1	1	1	1
0	0	1	0	1	1	1
1	1	1	0	0	1	1
0	1	1	0	0	1	0
1	1	1	0	0	1	0
0	0	1	0	0	0	0
			0			0

			0			0
			0			0
			1			1
1	1	1	0	0	0	1
0	0	1	1	0	0	1
0	0	1	0	0	0	0
0	0	1	0	0	0	0
1	1	1	0	0	0	1
			0			0
			0			1
0	0	1	0	1	1	1
1	1	1	0	1	1	0
0	1	1	0	1	1	0
1	1	1	0	0	1	0
0	1	1	1	1	1	1
0	0	1	0	0	1	0
0	0	1	1	0	1	1
0	0	1	0	0	1	1
0	0	1	0	0	1	0
0	0	1	0	0	1	1
0	0	1	0	0	1	0
1	1	1	0	0	1	1
0	0	1	0	0	1	0
1	1	1	0	0	0	1
1	0	1	0	0	1	1
0	0	1	0	0	1	0
0	0	1	1	1	1	1
0	0	1	0	0	1	1
0	0	1	0	1	1	0
1	1	1	0	0	0	1
1	0	1	0	0	0	0
1	0	1	1	0	1	1
0	0	1	0	1	1	1
0	0	1	0	0	0	0
0	0	1	0	0	0	0
1	1	1	1	0	1	0
0	0	1	1	0	0	1
0	0	1	0	0	1	1
1	0	1	0	0	1	1
0	0	1	0	1	1	1
1	0	1	1	0	0	0
			1			1
1	1	1	1	0	0	1
		1	1	0	0	1
0	0	1	1	1	1	0
0	0	1	0	0	0	1
0	0	1	0	0	1	0
0	0	1	1	0	1	1
1	1	1	0	0	0	0
0	0	1	1	0	1	1

1	1	0	1	0	0	1
0	1	1	1	0	0	1
0	1	1	0	0	0	0
1	1	1	0	0	0	1
1	1	1	1	0	0	1
0	0	1	0	0	0	1
0	0	1	0	0	0	1
1	1	1	0	0	0	1
1	0	1	0	0	0	0
1	0	1	0	0	0	0
1	0	1	0	0	1	1
0	0	1	0	0	0	1
0	0	1	0	0	0	1
1	1	1	0	0	1	0
0	0	1	0	1	1	0
1	1	1	0	0	0	0

1	0	1	0	0	1	0
1	1	1	1	1	0	0
0	1	1	0	0	0	0
1	1	1	0	0	0	1
0	0	1	0	0	0	0
0	0	1	1	0	0	0
1	1	1	1	0	0	1
1	0	1	1	0	0	1
1	0	1	1	0	0	1
0	0	1	0	0	0	1
1	0	1	0	0	1	0
1	0	1	0	0	0	0
0	0	1	1	0	0	1
1	0	1	0	0	0	1
1	0	1	0	0	0	1
0	0	1	1	1	0	1
1	1	1	1	0	0	1
1	1	1	0	0	0	0
0	0	1	0	0	0	0
0	0	1	1	0	0	0
1	0	1	1	0	0	1
0	0	1	1	0	1	0
1	1	1	1	0	0	1
			0			0
			1			1
			1			0
			1			1
			0			1
			1			1
			0			1
			1			1
			1			1
			0			1

1	0
0	1
0	0
1	1
1	0
0	1
1	0
0	0
1	1

1	1	1	0	0	0	1

Stuhl Kontrolle3	BZ Kontrolle3	Grippeimpfung3	Pneumoimpf 3	GebissoB3	GebissKontrolle3	Schmerz3
1	1	1	1	0	1	1
1	1	1	0	0	0	1
0	1	0	0	0	0	1
0	1	1	0	0	0	0
0	1	0	0	1	0	1
0	1	0	0	1	1	0
0	1	1	1	1	1	1
0	1	1	0	0	0	1
				1		
0	1	1	1	1	1	0
0	1	1	0	0	1	1
0	1			1	0	1
0	1	1	1	1	0	0
0	1	1	0	1	1	1
1	1	1	0	1	0	1
0	1	0	0	0	0	0
0	1	0	0	1	1	0
0	1	0	0	0	1	0
0	1	1	1	1	1	1
0	1	0	0	1	0	0
0	1	1	1	1	1	0
0	1	1	0	1	1	1
0	1	1	0	0	1	1
0	1	0	0	1	1	1
0	1	1	1	0	1	1
0	1	1	0	1	1	1
0	1	0	0	0	0	1
0	1	1	0	1	0	1
0	1	1	0	1	0	0
0	1	0	0	1	0	1
0	1	1	0	0	0	1
0	1	0	0	1	0	1
0	1	1	1	1	1	0
1	0	0	0	1	1	0
0	1	1	0	1	0	0
0	1	0	0	1	0	0
0	0	0	0	0	0	1
1	1	1	1	0	0	1
0	1	1	0	1	0	0
0	1	1	1	0	0	0
0	1	0	0	1	1	1
0	1	1	1	1	1	1
1	1	1	0	0	0	0
1	1	1	0	1	1	0
0	1	1	0	1	0	0
				1		

				1		
				1		
				1	0	
0	0	0	0	1	0	1
0	1	0	0	1	0	0
0	1	0	0	1	0	0
0	1	0	0	1	0	0
0	1	0	0	1	0	0
				0		
				1		
1	1	0	0	1	0	0
1	1	0	0	0	1	0
0	1	1	0	1	1	1
0	1	1	1	1	0	0
1	1	0	0	0	0	0
0	1	1	0	1	0	0
0	1	1	1	1	0	1
0	1	0	0	1	1	1
0	1	0	0	1	1	1
0	1	1	0	1	0	0
0	1	1	1	1	0	1
0	1	1	0	1	1	1
0	1	0	0	1	0	1
1	1	1	1	1	0	0
0	1	0	1	1	0	0
1	1	1	0	1	0	0
1	1	1	1	1	1	0
1	1	0	0	1	0	0
0	1	1	1	1	1	0
0	1	0	0	0	0	1
1	1	0	0	1	1	0
0	1	0	0	1	1	1
0	1	0	0	0	1	0
0	1	0	0	1	0	1
0	1	0	0	1	1	0
0	1	1	0	1	0	1
0	1	0	0	1	0	0
1	1	1	0	1	1	0
	1	1	1	1	0	0
0	1	0	0	1	1	1
0	1	1	0	1	1	0
				0		
0	0	0	0	1	0	1
0	1	0	0	0	0	0
0	1	1	0	1	1	0
0	1	0	0	1	0	0
0	1	1	0	1	1	0
0	1	0	0	1	0	0
0	1	1	0	1	0	1
0	1	0	0	1	0	0

0	0	0	0	1	0	1
0	1	0	0	0	1	0
0	1	0	0	1	0	0
0	0	0	0	1	0	1
0	1	0	0	1	0	0
0	1	1	0	1	1	0
0	1	0	0	0	0	0
0	0	0	0	0	0	0
0	1	0	0	1	0	0
0	1	1	0	1	0	1
1	1	0	0	1	0	0
0	0	0	0	0	1	0
0	1	0	0	1	0	1
0	1	0	0	0	0	1
0	1	0	0	1	1	1
0	0	0	0	1	0	0

0	0	0	0	1	0	0
0	1	0	1	1	0	0
0	1	0	0	1	0	1
0	1	0	0	1	0	0
0	1	0	0	0	0	0
0	1	0	0	1	0	1
0	0	0	0	1	0	0
0	1	0	0	1	0	0
0	1	0	0	1	0	1
0	1	0	0	1	0	0
0	1	0	0	1	0	0
0	1	0	0	0	0	1
0	1	0	1	0	0	0
0	1	0	0	1	0	0
0	1	0	0	0	0	0
1	1	0	0	0	1	1
0	1	0	0	1	0	0
0	1	0	0	1	0	1
0	1	0	0	1	0	0
0	1	0	0	0	0	0
1	1	0	0	0	0	1
0	1	0	0	1	0	0
0	1	0	0	1	0	0
				1		
				1		
				0		
				1		
				1		
				1		
				1		
				1		
				1		
				1		

1
0
1
1
1
0
0
1
1

0	0	0	0	1	0	0

Stürze3	Zahl Sturz3	Ursache Sturz3	Sturzangst3	Nikotin3	Pack years3	Wunsch abgewöhnen 3
0	3	4	1	2	0	999
0	1	4	0	1	137,5	999
1	0		1	2	0	999
1	0		1	2	0	999
1	0		1	2	0	999
0	1	4	0	2	0	999
0	1	3	0	2	0	999
0	2	4	1	2	0	999
				2	0	999
0	5	2,4,5	0	1	2,5	999
0	1	5	1	2	0	999
1	0		1	2	0	999
0	1	3	1	2	0	999
1	0		0	2	0	999
0	4	2,3	0	2	0	999
1	0		0	2	0	999
1	0		1	2	0	999
0	1	4	1	2	0	999
1	0		1	1	8	999
0	4	1,2,3	0	2	0	999
1	0		1	2	0	999
1	0		0	2	0	999
1	0		1	1	45	999
1	0		1	1	20	999
0	1	1	1	2	0	999
1	0		1	1	0,75	999
0	1	4	0	2	0	999
0	3	3	1	2	0	999
1	0		0	1	3	999
0	2	1	0	2	0	999
0	4		0	2	0	999
0	1	4	1	1	15	999
0	1	4	1	2	0	999
0	3	2,4,	0	2	0	999
1	0		0	2	0	999
				2	0	999
1	0		1	2	0	999
0	8	2,3,4	1	2	0	999
1	0		1	1	14	999
1	0		0	2	0	999
0	2	4	1	1	8,8	999
1	0		0	2	0	999
0	6	5	0	2	0	999
1	0		1	2	0	999
0	1	3	0	2	0	999
				2	0	999

				1	12	999
				2	0	999
1	0		0	2	0	999
1	0		1	2	0	999
0	1	2	0	2	0	999
1	3	3,5	0	2	0	999
0	1	5	0	1	20	999
1	0		1	1	78	999
				1	8	999
				2	0	999
0	2	1,4	0	2	0	999
1	0		0	2	0	999
1	0		0	2	0	999
0	3	3,4	0	1	7,5	999
0	3	2	1	1	5	999
0	3	2,4	0	0	30	0
1	0		0	1	10	999
1	0		1	2	0	999
1	0		1	2	0	999
0	3	2	0	1	7	999
0	1	4	0	2	0	999
			1	1	35	999
1	0		0	2	0	999
1	0		1	0	12	0
1	0		0	2	0	999
0	1	3	0	2	0	999
0	3	2,5	0	2	0	999
0	8	1	1	2	0	999
0	1	4	0	2	0	999
1	0		0	2	0	999
1	0		1	2	0	999
1	0		1	1	20	999
0	8	1	0	2	0	999
0	3	2,3,	0	1	1,65	999
0	1	5	1	2	0	999
1	0		0	2	0	999
0	2	1,2,	0	2	0	999
1	0		1	2	0	999
1	0		1	2	0	999
0	2	1,5	0	2	0	999
0	1		0	2	0	999
				2	0	999
1	0		1	2	0	999
0	1	4	0	2	0	999
0	3	1	0	2	0	999
0	1	5	0	2	0	999
0	1	4	0	2	0	999
0	3	2	0	2	0	999
0	1	2	0	2	0	999
0	1	2	0	2	0	999

1	0		0	2	0	999
0	1	4	0	1	18	999
0	3		0	2	0	999
1	0		1	2	0	999
0	1	4	1	2	0	999
1	0		1	2	0	999
1	0		0	2	0	999
1	0		0	2	0	999
0	1	2	0	1	18	999
0	1	4	1	1	2	999
1	0		0	2	0	999
1	0		1	2	0	999
0	1		1	2	0	999
0	22 (?)		1	2	0	999
0	2	1,2	1	2	0	999
1	0		1	2	0	999
0		3	0	2	0	999
1	0		1	2	0	999
0	3	3	0	2	0	999
				1	26	999
1	0		0	2	0	999
0	4	2	1	2	0	999
1	0		0	2	0	999
1	0		1	2	0	999
0	1		0	2	0	999
0	2	4	1	2	0	999
0	2	3	0	1	6	999
1	0		0	1	0,75	999
1	0		0	2	0	999
1	0		0	1	35	999
0	3	1,2	0	2	0	999
1	0		1	1	5	999
1	0		0	1	35	999
1	0		0	2	0	999
1	0		0	2	0	999
1	0		1	2	0	999
0	6	2,3	0	2	0	999
1	0		0	2	0	999
0	1	3	0	2	0	999
				2	0	999
				2	0	999
				2	0	999
				2	0	999
				1	25	999
				1	7,5	999
				2	0	999
				2	0	999
				1	28	999
				2	0	999

1	7,5	999
2	0	999
1	5	999
2	0	999
1	15	999
2	0	999
2	0	999
2	0	999
1	5	999

1	0	0	1	1	2,8	0

Augen gut3	Augen Kontrolle3	Ohren gut3	Ohren Kontrolle3	Anschnallen3	Essen fett3	Essen Körner3
1	1	0	0	1	1	0
1	0	1	1	1	1	0
0	0	2	0	1	1	0
1	0	2	0	1	1	0
1	0	2	0	1	1	0
1	0	2	0	1	1	0
0	0	1	0	1	1	0
0	1	0	1	1	1	0
0		2		1		
1	0	2	1	1	1	0
1	1	1	1	1	1	0
1	0	0	0	1	1	0
1	0	2	0	1	1	0
1	1	1	0	1	1	0
2	1	2	0	1	1	0
0	1	0	0	1	0	0
1	1	0	1	1	1	0
1	0	2	0	1	1	0
2	0	2	0	1	1	0
0	0	0	0	1	1	0
0	1	0	1	1	1	0
1	1	2	0	1	1	0
0	1	0	0	1	1	0
1	0	2	0	1	1	0
0	0	2	1	1	1	1
1	1	0	1	1	1	0
1	0	0	0	1	1	0
0	0	0	0	1	1	0
1	0	0	0	1	1	0
0	0	2	0	1	1	0
1	0	0	0	1	1	0
0	0	1	0	1	1	0
1	0	2	1	1	1	0
2	0	2	1	1	1	0
0	0	2	0	1	1	0
1	0	1	1	1	1	0
0	1	0	1	1	1	0
1	0	1	1	1	1	0
0	0	2	0	1	0	0
0	1	0	0	1	1	0
1	1	1	0	1	1	0
1	1	0	1	1	1	0
1	1	0	0	1	1	0
0	0	2	0	1	1	0
1	1	2	0	1	1	0
1		2		1		

1		2		1		
0		1		1		
1	0	0	0	1		
1	0	1	0	1	1	0
1	0	2	0	1	1	0
0	1	2	0	1	1	0
1	0	2	0	1	1	0
0	0	1	0	1	1	0
1		0		1		
0		0		1		
1	0	1	0	1	1	0
0	1	2	1	1	1	0
0	0	0	1	1	1	0
1	1	2	0	1	1	0
1	0	2	1	1	1	0
1	0	2	0	1	1	0
1	0	1	0	1	1	0
1	0	2	0	1	0	0
1	0	0	0	1	1	0
1	1	1	0	1	0	0
1	0	1	0	1	1	0
1	0	2	0	0	1	0
2	0	0	0	1	1	0
1	0	1	0	1	1	0
0	1	1	0	1	1	0
0	1	1	1	1	1	0
0	0	0	1	1	1	0
1	0	2	0	1	1	0
0	1	0	1	1	1	0
1	0	2	0	1	1	0
1	0	0	1	0	0	0
2	0	2	1	1	1	0
0	1	2	0	1	1	0
0	0	2	0	1	1	0
1	1	0	0	1	1	0
0	0	2	0	1	1	0
1	1	2	0	1	1	0
2	0	2	0	1	1	0
0	0	0	0	1	1	0
1	1	0	0	1	1	0
1	1	2	0	1	1	0
0		1		1		
2	0	0	0	1	0	0
1	0	2	0	1	1	0
0	1	0	0	1	1	0
1	0	2	0	1	1	0
0	1	0	1	1	1	0
1	0	1	0	1	0	0
0	1	2	0	1	1	0
1	1	2	0	1	1	0

2	0	2	0	1	1	0
0	0	2	0	1	1	0
0	0	0	0	1	1	0
1	0	0	0		1	0
1	0	2	0	1	1	0
1	0	2	0	1	1	0
0	0	2	0	1	1	0
1	0	1	0	1	1	0
0	0	0	0	1	1	0
0	0	2	0	1	1	0
1	0	1	0	1	1	0
0	0	2	0	1	1	0
1	0	2	0	1	1	0
0	0	2	0	1	1	0
0	1	2	0		1	0
1	0	2	0	1	1	0
1	0	1	0	1	1	0
1	0	2	0	1	1	0
1	0	1	0	1	1	0
0	0	2	0	1	1	0
	0	1	0	1	1	0
0	0	0	0	1	1	0
1	0	1	0	1	1	0
1	0	0	0	1	1	0
2	0	0	0	1	1	0
2	0	2	0	1	0	0
0	0	2	0	1	1	0
0	0	2	0	1	1	0
1	0	1	0	1	1	0
0	0	1	0	1	1	0
1	0	0	0	1	1	0
1	0	2	0	1	1	0
1	0	2	0	1	1	0
1	0	1	0	1	1	0
1	0	2	0	1	1	0
1	0	0	0	0	1	0
0	0	2	0	1	1	0
0	0	0	0	1	1	0
0	1	0	0	1	1	0
2		2		1		
0		2		1		
1		0		1		
2		2		1		
1		2		1		
1		2		1		
0		0		1		
1		2		1		
2		2		1		
1		1		1		

0	1	1
0	2	1
1	2	1
0	2	1
0	1	1
1	2	1
1	1	1
0	0	1
1	2	1

0	1	0	0	1	1	0

Essen ändern3	MAPE3-Alkohol	MAPE3-Medis	MAPE3-Schlafmittel	MAPE3-Beruhigung	MAPE3-Opioid	MAPE3-Abführ
0	6	0	0	0	0	0
0	0	12	4	4	0	4
0	7	4	4	0	0	0
0	0	4	4	0	0	0
0	6	7	4	0	0	3
0	3	7	0	0	4	3
0	9	0	0	0	0	0
0	0	0	0	0	0	0
	0	1	0	0	1	0
0	9	8	0	0	4	4
0	4	0	0	0	0	0
0	0	4	0	4	0	0
0	0	0	0	0	0	0
0	6	12	4	4	0	4
0	0	7	4	0	0	3
0	0	4	0	4	0	0
1	0	7	0	4	0	3
0	3	0	0	0	0	0
0	6	0	0	0	0	0
0	0	4	0	0	4	0
0	0	0	0	0	0	0
0	3	4	0	0	0	4
0	7	8	4	0	0	4
0	7	0	0	0	0	0
0	0	0	0	0	0	0
0	9	0	0	0	0	0
0	0	8	4	0	4	0
0	0	0	0	0	0	0
0	7	3	0	0	0	3
0	7	3	3	0	0	0
0	2	8	4	4	0	0
0	10	0	0	0	0	0
0	0	4	4	0	0	0
0	3	0	0	0	0	0
0	0	8	0	0	4	4
0	0	5	4	0	0	1
0	3	4	4	0	0	0
0	0	7	4	0	0	3
0	0	4	0	0	4	0
0	0	9	4	0	2	3
0	8	3	0	0	0	3
0	0	4	0	4	0	0
0	4	4	0	0	0	4
0	3	4	4	0	0	0
0	0	0	0	0	0	0

	0	11	4	4	0	3
0	3	6	3	0	0	3
0	0	8	0	0	4	4
0	0	0	0	0	0	0
0	0	3	3	0	0	0
0	7	7	4	0	0	3
0	0	7	0	0	4	3
0	0	11	4	4	0	3
0	3	4	0	0	4	0
0	3	3	0	0	0	3
0	6	4	0	0	0	4
0	0	4	2	0	2	0
0	3	0	0	0	0	0
0	10	0	0	0	0	0
0	3	8	4	0	0	4
1	6	7	4	0	0	3
0	8	0	0	0	0	0
0	4	3	3	0	0	0
0	0	0	0	0	0	0
0	0	4	4	0	0	0
0	0	6	2	4	0	0
0	6	6	0	0	2	4
0	0	8	0	0	4	4
0	7	0	0	0	0	0
0	6	10	4	0	3	3
1	0	4	4	0	0	0
0	5	7	0	4	0	3
0	10	3	0	0	0	3
0	0	3	0	0	0	3
0	6	3	0	0	0	3
0	0	0	0	0	0	0
0	3	0	0	0	0	0
0	3	8	4	0	4	0
0	3	4	0	0	4	0
0	3	1	0	0	1	0
0	3	1	1	0	0	0
0	0	4	4	0	0	0
0	9	4	1	0	0	3
0	0	13	4	4	4	1
0	0	8	4	0	4	0
0	0	4	4	0	0	0
0	0	7	0	4	0	3
0	0	0	0	0	0	0
0	9	0	0	0	0	0
0	7	7	0	0	4	3

0	10	8	4	0	0	4
0	7	3	0	0	0	3
0	0	10	4	3	0	3
0	0	7	4	0	0	3
0	1	0	0	0	0	0
0	0	4	0	0	0	4
0	3	8	4	0	4	0
0	0	9	4	4	0	1
0	3	3	0	0	0	3
0	0	4	4	0	0	0
0	0	4	0	0	4	0
0	3	1	1	0	0	0
0	0	7	3	0	0	4
0	3	3	0	0	0	3
0	0	0	0	0	0	0
0	5	11	4	4	0	3

0	0	10	3	0	4	3
0	9	11	4		4	3
0	0	7	4	0	0	3
0	0	11	4	0	4	3
0	0	9	4	0	2	3
0	0	6	0	0	2	4
0	8	10	0	4	3	3
0	3	12	4	4	0	4
0	1	9	3	0	3	3
0	2	7	0	3	4	0
0	0	7	3	0	4	0
0	1	7	4	0	0	3
0	11	7	4	0	0	3
0	0	4	0	0	4	0
0	0	12	4	1	4	3
0	0	4	0	0	0	4
0	3	8	4	0	0	4
0	0	12	4	4	0	4
0	6	4	4	0	0	0
0	3	7	3	0	0	4
0	0	7	0	0	4	3
0	10	0	0	0	0	0
0	0	12	4	4	0	4

0	8	7	4	3	0	0

AnzahlTabl.ges3	Anzahl Medikamente3	Antidepressiva?3	Seit wann AD?3	SSRI/SNRI3
11,5	9			
21	11			
14	9			
19	13			
14,5	10			
19,5	9			
5,5	5			
9,5	7			
19,75	14			
12,5	12			
4	4			
24	12			
6	6			
27,5	16			
10	10			
17	12			
9	8			
15	10			
7	7			
16	12			
20,5	12			
16	8			
22	15			
4	5			
9,5	9			
4	4			
16	8			
6,5	5			
15,5	14			
6,5	6			
15,5	12			
13	9			
15	21			
18,25	12			
8,5	8			
12	11			
19	9			
10,5	9			
5	6			
11	12			
12	11			
5	5			
9	9			

4	3
6,5	7
14,5	12
8,5	6
13	7
26	15
7,5	6
13	9
10	8
7	6
7	7
5	4
8	8
19	12
6	8
20,5	14
6,5	6
4	3
16,5	10
14	11
15	12
18	12
12	8
6,5	7
9	10
10,5	10
6,5	5
19	5
5	5
19	15
11	10
14,5	10
12,5	12
4,5	4
14	10
16,5	11
5	4
15,5	12
10	7
4	3
10	9

19	11
7	5
7,75	10
	5
14	8
12	7
15	11
14	9
	12
24	16
24	13
1	3
12,5	8
18	12
7	7

11,5	10
12	
6	5
11,5	7
2	2
11	8
10	8
15,5	11
9	6
24,5	15
14	9
12	8
22,5	15
10,5	11
10,5	9
10	9
14	9
17,5	10

5,5	5	0

Mirtazapin3	Trizyklika3	Benzos/3Z3	PflanzlichLithium3	Typische Neuroleptika 3	Atypische Neuroleptika 3	CIRS3

Pearlin3	MEL-life events3	Karnofsky3	PSP3	Frailty-Index3	TimedUGTest 3	GehUZT3-4m
11		60			14	4
15					10	5
15		80			15	4
15		75			19	9
15						
12					30	10
13					13	7
14					10	4
18					59	40
19					10	5
20					14	7
5		30	31			
12					15	7
11						
8		50	41		54	24
6						
15		80			6	3
17					23	8
13					15	7
7						
15		80	80		16	7
18					11	5
15					18	6
19		80	80		11	5
13		80	80		17	9
20					11	5
13		60	60		22	11
17						30
15		60	60		34	17
10		55	51		19	7
10		80	70		13	5
16		80	80		11	4
12		75	70		13	8
13		40	41			
13		60	61		23	11
19		75	70		26	11
12		55	61		10	4
12		80	70		7	4
20		55	61		18	9
10		50	41		17	5
19		75	70		16	6
15		70	70		13	5
13		75	70		19	7
15		80	70		7	5
10		55	61		22	7

15					
14	145	35	30		
15		55	61	21	10
11		60	50	35	12
16		35	11	26	13
11				29	7
12				15	6
15				14	6
12				20	7
15				9	5
12					
15				17	6
20				18	6
15					
15					
7				14	6
13		75	71	10	3
10		60	51	22	6
11		55	61	26	12
10		50	40	16	6
11				21	8
12		55	60	75	20
18		55	60	29	8
9		75	50	19	6
17		60	70	9	5
8		45	30	25	19
19		75	80	17	5
15			41	18	5
15		70	80	13	4
16		65	71	10	6
13		60	61	29	19
10		40	30		
12		40	41		
8		60	61	27	9
13		75	70	26	12
9		60	61	29	10
15		70	51	16	8
11		40	21	35	12
5				16	15
5		10	1		
10				21	7
9		40	21		
12		40	31		
15		60	61	22	8

11	153	65	41		22	9
15		60	50		23	14
12	296	35	21			
12	145	40	31		22	12
14		60	50		22	13
15		50	50			
7		40	21		44	12
5	90	35	21	> 120 s		35
10	90	40	30		35	13
15		40	21		19	7
15		40	41		41	14
14	97	80	80		10	4
15		55	60		30	11
8		35	30			
10					29	9
10	53	60	61		13	7
5	178	40	11		38	25
10		55	50		16	8
10		45	50		24	10
12	190	40	41			
13	190	40	30			
13		60	70		24	12
17		60	60		10	7
13		45	31		30	16
13		40	21		27	15
16	97	50	41		18	18
9	189	35	30			18
14		55	50		20	10
6		35	21		42	31
11	100	55	30		24	13
11		50	41		27	15
11		35				
14		40	31		28	20
13		50	41		13	7
12		50	41		32	13
15		45	40		29	18
14		45	41		32	12
9	96	35	30		18	11
12	90	35	21		42	18

12 23 14

GehUZT3-plusZähl	GehUZT3-Zahlrichtig	GehUZT3-Zahl falsch	Tinnetti3	5chairrise3	Handkraft re3a	Handkraft re 3b
11	4	1		13	48	48
5	5	0		16	60	70
10	3	1		11	90	70
10	7	0	22	18	54	50
					46	48
11	7	0		61	20	20
27	6	0		17	50	55
14	5	0		16	50	48
43	7	0			44	40
8	7	0		10	52	44
7	7	0		11	48	54
			1			
9	3	1		18	26	30
					16	14
34	0	5			34	16
					18	18
4	4	0		15	4	2
9	2	0		23	60	60
14	5	0		24	70	68
					2	0
12			26	13	52	64
15	4	0		12	42	40
18	5	1		24	50	50
6			28	11	80	75
13				36	34	36
7	6	0		12	50	54
12			18	16	50	50
30					42	48
21				16	40	40
11				27	38	30
9			21	21	36	40
8				13	60	62
31				25	30	38
					20	18
16	3	0		25	48	40
13	6	0		19	45	42
8				11	30	32
5				13	65	60
11				13	60	62
8				36	18	6
5				16	68	60
11	7	0		16	48	46
9	9	0		20	53	55
10				25	2	0
14				25	45	48

					4	2
			1		15	20
14			20	36	20	20
20	2	0	18	30	60	50
15			15	26	50	50
					30	30
8	3	0		19	50	50
8	5	0		17	50	50
7	6	1		18	70	72
6	7	0		14	90	100
					50	54
14	5	1		30	54	50
11	4	1		16	80	70
					14	16
					12	20
			14	15	60	80
6			28	17	42	36
8	5	1		21	46	46
14			13	34	30	30
12				22	68	62
13	4	1	16	48	50	35
29				51	4	2
19	4	1		23	0	0
10				23	56	48
5		28?		12	40	35
				26	46	40
7				16	90	92
8				17	10	10
6	5	0		16	48	50
11				15	40	48
26	2	0	9	33	40	40
			2		40	35
					4	2
17				33	28	26
15			12	54	50	50
12	5	0		50	38	35
12	2	0	19	16	30	30
			10		28	25
10	5	0		15	40	45
			0		0	0
11	3	0		18	60	62
					42	48
					1	0
10	3	0		19	20	20

13			14	20	50	55
					50	50
			2		5	0
13			10	20	60	50
19	1	0		32	20	20
					14	28
21	0	8		30	15	10
87			4	90	10	5
17			7	34	50	40
25			10	25	40	30
19			16	38	2	2
11			26	12	50	50
15	3	0	18	26	40	30
			1		20	18
13	1	1		21	40	52
7			23	12	45	30
			10	115	10	0
12	2	0	17	34	50	35
12	2	0	17	28	60	50
					8	5
			1		20	15
13				18	48	56
8			18	15	30	20
42	2	0	12	28	40	40
23			12	38	20	20
25			11		10	15
19			4		30	20
18			22	30	20	10
40			6	60	10	5
22				35	15	10
23			8	45	50	35
					0	
22			8	35	10	10
11			20	27	30	20
21			11	40	20	20
24			17	38	60	60
17			17	44	70	60
14			16	42	30	20
21			9	46	30	30

26	2	0	12	30	20

Handkraft li 3a	Handkraft li3b	BMI3	RRmittelsyst 3	RRmitteldiast 3	RRstehsyst3
58	58	19,9	130	80	130
80	70	22,7	100	60	100
50	35	26,4	130	80	120
44	34	29,6	150	80	170
14	16	27,9	130	80	
42	48	32,1	110	80	110
50	48	22,2	150	80	130
40	40	21,6	110	80	120
30	26	29,8			
32	32	29,3			
48	46	26,1	115	75	120
		14,1			
38	38	43,1	125	65	110
12	10	30,8	130	90	
4	8	18,75			
2	2	32,5			
16	8	34	140	70	120
34	30	28,7			
64	62	26,7	130	80	130
0	0	35,7			
68	68	24,4	110	70	120
52	52	23,1			
28	18	35	130	70	100
74	68	23,2			
14	14	25	110	70	110
46	50	25,3			
50	50	36,1	140	80	150
38	38	24,2	130	90	130
40	48	27,8	140	70	140
52	46	28,5	130	90	130
46	38	33,1			
62	60	21	130	80	140
30	34	30,4	130	80	120
40	36	39,5	130	80	140
50	50	27,7	130	80	140
60	62	29			
24	32	16			
40	30	20,7	140	80	140
64	62	46,7	120	80	150
8	4	34,6	140	80	150
0	0	25,9	140	80	140
20	24	22,9			
40	45	30,5	140	80	140
42	46	30,8	150	80	130
38	34	19,8			

0	0	17,5			
15	10	28,3	110	60	
10	5	35,2	150	80	160
50	40	25,4	140	80	130
50	40	23,1	120	70	110
45	45	24,7	160	60	
50	45	17,1	150	80	140
60	58	30,5	120	70	110
48	52	34,7	130	90	130
84	84	23,8	160	90	150
30	20	29,3			
34	24	20			
68	64	39,9	140	80	
4	2	30,8	160	70	
60	55	24,4			
80	70	25,1			
20	24	21,3	110	70	100
34	36	36,9	110	70	100
30	20	27,6	100	60	100
66	64	32,3	140	80	150
50	70	31,5	100	60	80
18	16	29,9	110	80	120
0	0	35,7	110	60	110
28	32	26,8	130	70	140
25	25	16,8	120	60	110
20	18	37,6	140	90	150
80	74	25,7	140	80	150
50	50	33,3			
60	62	29,4	130	90	140
50	54	20,5	110	60	120
30	35	22,3	120	60	110
40	35	26,1	100	60	
14	12	23,2			
26	26	20,8			
30	20	30,3	130	70	140
30	27	20	12	60	110
40	30	20,7	120	70	110
22	14	17,8			
35	40	21,5	110	70	100
0	0	14			
50	58	43,1	110	60	120
36	38	32,4			
0	0	20,9	120	80	
15	15	33,3	120	80	130

55	50	21,1	110	60	110
35	20	26	130	70	140
5	0	17,9	110	60	
30	20	18,3	110	60	130
4	2	24,2	110	60	110
4	0	32,3	110	70	
2	0	32,1			
5	5	28,1	130	80	140
40	20	22,9	120	60	110
20	10	30,9	140	60	150
2	0	27,3	130	60	140
50	45	29,7	150	70	130
30	20	23,8	120	60	130
15	15	24,4	110	70	
65	62	26,7	110	70	130
30	30	24,5	110	60	110
10	10	32,4	100	60	110
35	30	23	120	60	110
60	50	23,2	140	80	150
20	15	28,1	110	70	
15	10	17,4	110	70	90
46	48	29,9	130	90	140
20	15	21,6	130	70	110
25	20	20,4	110	60	110
20	15	23,6	110	60	100
10	0	36,9	110	60	100
30	20	29,4	110	60	
60	50	24	120	60	110
10	5	18,4	110	60	110
0	2	19,9	110	60	110
40	35	17,7	110	60	110
0		22,6	110	70	
30	25	23,1	110	70	130
30	20	30,1	120	60	130
10	10	33,4	110	60	110
50	50	24	120	60	110
60	50	25,6	110	50	100
20	20	26,5	140	80	140
30	20	24,5	110	50	100

80	80	110	80	110

RRstehdiast3	Puls3
80	56
70	72
80	72
80	72
	72
80	
70	68
80	100
80	64
60	51
	68
60	56
80	76
70	68
60	68
60	72
70	76
80	68
80	
80	72
90	68
70	64
90	72
90	68
90	76
100	68
70	64
80	76
70	72
90	68

	72
80	68
90	76
70	72
90	60
60	64
90	64
80	72
	56
60	64
70	72
50	64
90	76
60	80
80	76
55	76
80	72
70	72
80	64
90	80
80	64
80	80
60	72
	68
60	72
60	64
70	64
60	72
90	84
	64
90	60

70	72
70	72
	68
60	68
60	68
	76
90	64
50	60
60	72
60	68
80	60
70	88
	72
90	56
60	68
50	84
50	72
90	60
	64
60	
80	76
60	88
50	68
60	72
60	68
	68
60	72
50	72
60	68
50	68
	76
80	68
60	72
60	72
50	68
50	68
70	72
50	68

80 68
