# Supplementary material for: AIDElong—acute illness and depression in elderly: sustained improvement after group psychotherapy in geriatric patients, a follow-up of longterm effects in a randomized controlled trial
Source: BMC Geriatr. 2026 Feb 7;26:329. doi: 10.1186/s12877-026-06983-0 (PMC12983662; doi:10.1186/s12877-026-06983-0)
Supplement: Supplementary file 2 — Supplementary Material 2. [file 12877_2026_6983_MOESM2_ESM.pdf]

CONSORT Flow Diagram

Flow diagram of the progress through the phases of a randomised trial of two groups (that is, enrolment, intervention allocation, follow-up, and data analysis)

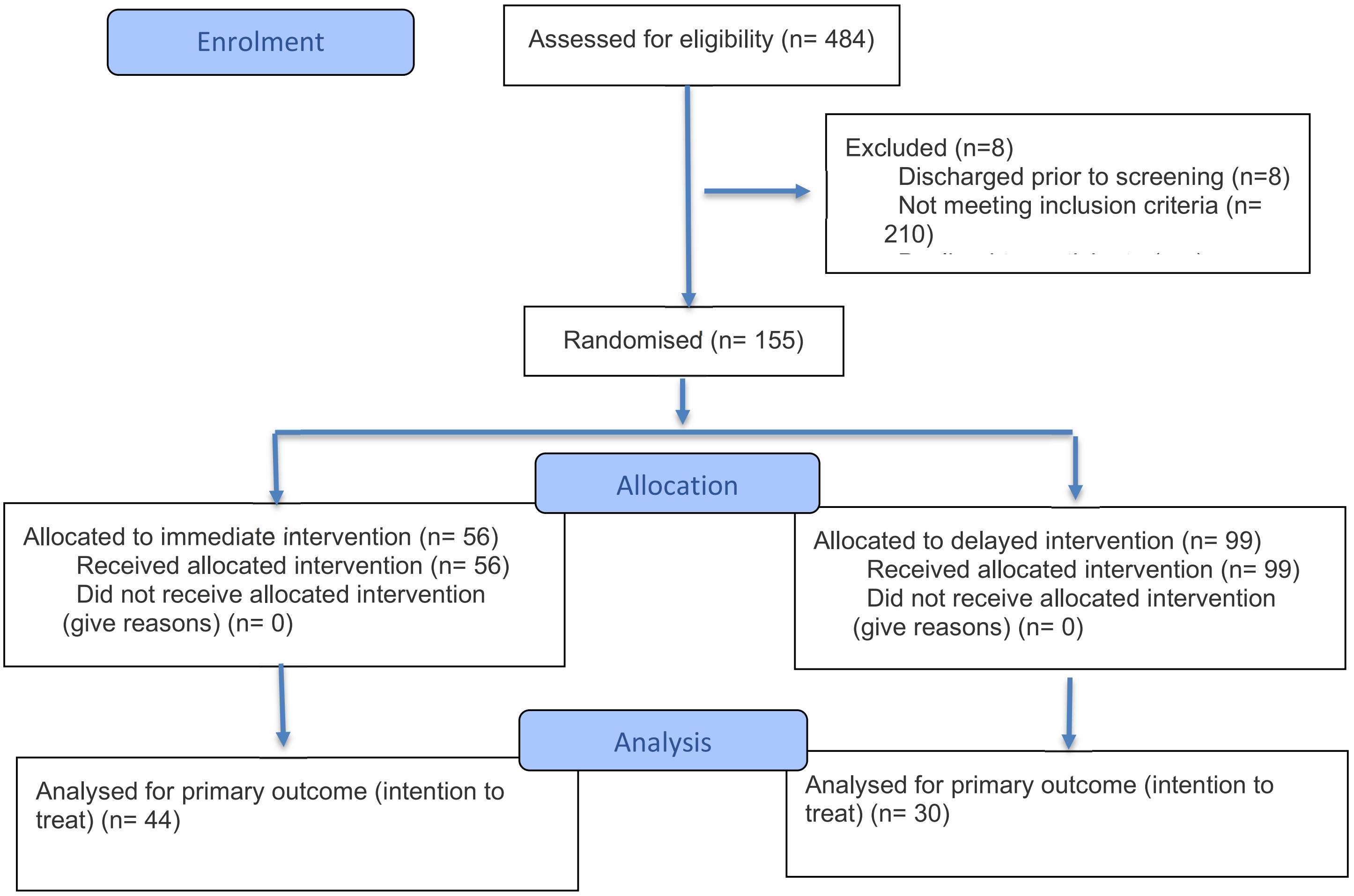

Citation: Hopewell S, Chan AW, Collins GS, Hróbjartsson A, Moher D, Schulz KF, et al. CONSORT 2025 Statement: updated guideline for reporting randomised trials. BMJ. 2025; 388:e081123. https://dx.doi.org/10.1136/bmj-2024-081123

© 2025 Hopewell et al. This is an Open Access article distributed under the terms of the Creative Commons Attribution License (https://creativecommons.org/licenses/by/4.0/), which permits unrestricted use, distribution, and reproduction in any medium, provided the original work is properly cited.
